# NIR-II cell endocytosis-activated fluorescent probes for *in vivo* high-contrast bioimaging diagnostics†

**DOI:** 10.1039/d1sc02763h

**Published:** 2021-06-30

**Authors:** Yue He, Shangfeng Wang, Peng Yu, Kui Yan, Jiang Ming, Chenzhi Yao, Zuyang He, Ahmed Mohamed El-Toni, Aslam Khan, Xinyan Zhu, Caixia Sun, Zuhai Lei, Fan Zhang

**Affiliations:** Department of Chemistry, State Key Laboratory of Molecular Engineering of Polymers and iChem, Shanghai Key Laboratory of Molecular Catalysis and Innovative Materials, Fudan University Shanghai 200433 China sfwang@fudan.edu.cn zhang_fan@fudan.edu.cn; King Abdullah Institute for Nanotechnology, King Saud University Riyadh 11451 Saudi Arabia

## Abstract

Fluorescence probes have great potential to empower bioimaging, precision clinical diagnostics and surgery. However, current probes are limited to *in vivo* high-contrast diagnostics, due to the substantial background interference from tissue scattering and nonspecific activation in blood and normal tissues. Here, we developed a kind of cell endocytosis-activated fluorescence (CEAF) probe, which consists of a hydrophilic polymer unit and an acid pH-sensitive small-molecule fluorescent moiety that operates in the “tissue-transparent” second near-infrared (NIR-II) window. The CEAF probe stably presents in the form of quenched nanoaggregates in water and blood, and can be selectively activated and retained in lysosomes through cell endocytosis, driven by a synergetic mechanism of disaggregation and protonation. *In vivo* imaging of tumor and inflammation with a passive-targeting and affinity-tagged CEAF probe, respectively, yields highly specific signals with target-to-background ratios over 15 and prolonged observation time up to 35 hours, enabling positive implications for surgical, diagnostic and fundamental biomedical studies.

## Abstract 2

A Cell Endocytosis-Activated Fluorescent (CEAF) probe triggered by disaggregation and protonation is designed for high contrast *in vivo* bioimaging and diagnostics in the second near-infrared window (1000–1700 nm).

## Graphical abstract

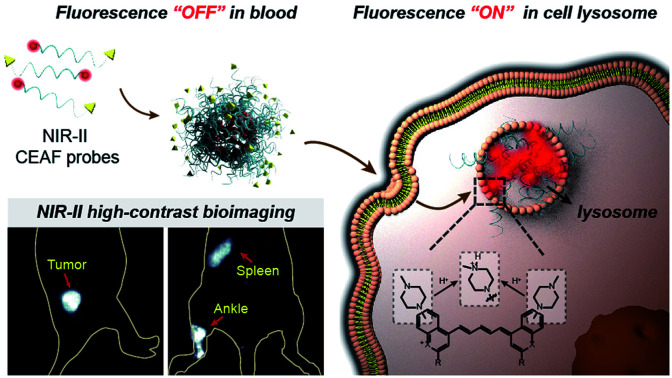

## Introduction

Fluorescence imaging has emerged as a powerful tool for preclinical *in vivo* imaging and as a promising clinical technology, particularly for surgical guidance.^1–3^ Systemically delivered fluorescence probes have the potential to broadly highlight disease sites without any previous knowledge of disease location. Several FDA-approved dyes such as indocyanine green (ICG) tagged affinity agents have afforded substantial clinical benefits,^4,5^ but the main limitation is their overall non-specific fluorescence background that results in insufficient sensitivity. Comparing with “always-on” fluorescence,^6^ activatable fluorescence probes that specifically respond to biomarkers in disease progress such as reactive oxygen species^7^ and proteases^8^ can reduce off-target background signals, which leads to further increased imaging contrast.^9–14^ However, this original intention is usually compromised and limited in its ability to diagnose diverse diseases due to the complex and heterogeneous physiological environment *in vivo*.^15,16^ In particular, most activatable probes tend to nonspecifically bind with the ubiquitous biomacromolecules (*e.g.*, serum albumins, lipoproteins) in blood, which may trigger unwanted fluorescence and complicate the pharmacokinetics, resulting in short-term retention in lesions and low target-to-background ratio (TBR).^17–19^ More importantly, currently available activatable probes mostly emit visible (VIS; 400–700 nm) and near-infrared (NIR-I; 700–900 nm) signals that could be significantly altered by tissue heterogeneities and depth location, leading to limited TBR *in vivo*.^20,21^

**Scheme 1:**
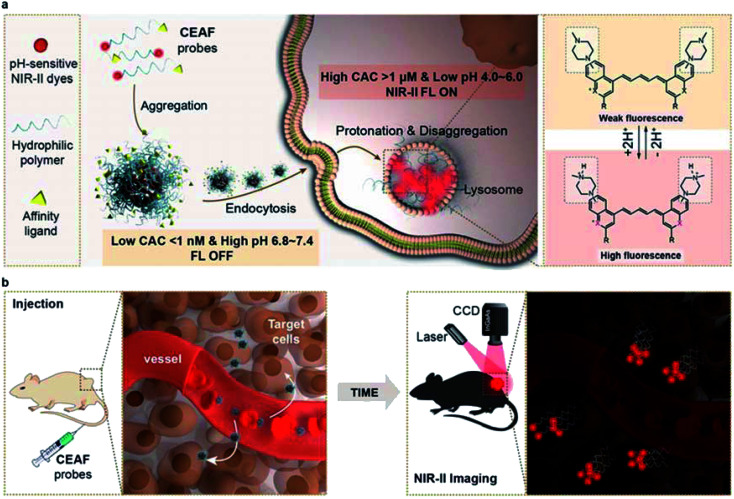
Schematic diagram of the CEAF mechanism. (a) The CEAF probes consist of a functionalized hydrophilic polymer unit and a pH-sensitive small-molecule fluorescent moiety with NIR-II emission, and stably present in the form of quenched nanoaggregates in water and blood due to an ultralow critical aggregate concentration (CAC). After endocytosis by target cells, the nanoaggregates disaggregate into protonated fluorescent monomers and are retained in the cell lysosome due to the significantly increased CAC. (b) *In vivo* imaging with CEAF probes allows low fluorescence background in blood, improving the sensitivity and contrast for cell-specific diagnostics.

In this work, we develop a series of Cell Endocytosis-Activated Fluorescent (CEAF) probes that operate in the “tissue-transparent” NIR-II (1000–1700 nm) window to minimize the background signal and maximize the TBR. We rationally design and optimize an acidic pH-sensitive NIR-II fluorophore and by further conjugating a functionalized polyethylene glycol unit, we synthesize the amphiphilic CEAF probes which exhibit unexpectedly ultralow critical aggregation concentration (CAC) below 1 nM in water. This characteristic allows the probes to be stably present in the form of quenched nanoaggregates in blood (Scheme 1), while disaggregation and fluorescence activation (up to 72-fold) selectively occur in cell endocytosis, driven by the increased CAC value in lysosomes due to the enhanced noncovalent interaction and acid strength. Notably, protonation of fluorophores therein further produces approximately 1.5-fold fluorescence enhancement, enabling amplified fluorescence activation inside cells (up to 108-fold) and long-term intracellular retention. We demonstrate the image-guided surgery application of a passive-targeting probe CEAF-OMe for precisely locating tumors, enabling clear resection margins with no risk of fluorescence contamination from intraoperative bleeding. In addition, by using the probe CEAF-RGD with specific affinity for M1 macrophage, we realize cell-specific diagnosis of traumatic arthritis *in vivo* with high TBR over 15.

## Results and discussion

Lysosomes are the acidic organelles at the end of the endocytic pathway, in which substances are surrounded by an area of lipid membrane and frequently interact with the densely distributed acid hydrolases and lysosomal catabolites, such as free fatty acids and lipids.^22–25^ We reasoned that this unique microenvironment distinct from the extracellular matrix and blood could be explored as a trigger for fluorescence activation.^26–29^

### Preparation and characterization of pH-sensitive and lysosomal-targeting NIR lyso dyes

**Fig. 1:**
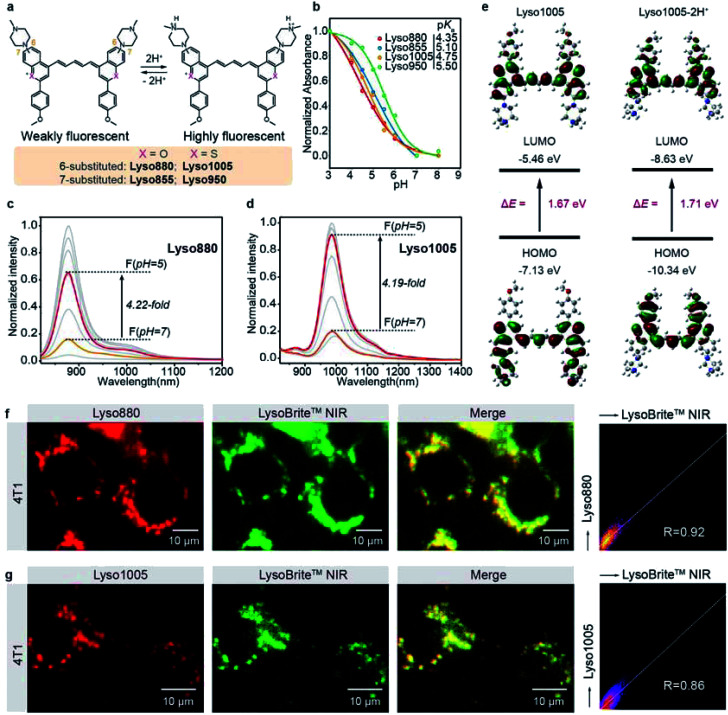
(a) Chemical structures of the pH-sensitive NIR-II dyes (Lyso880, Lyso1005, Lyso855, Lyso950), with the corresponding protonation process. (b) pH-dependent changes in the absorbance of these dyes, with their respective p*K*_a_ values. Normalized fluorescence spectra of Lyso880 (c) and Lyso1005 (d) in the pH range of 3–8 under 808 nm excitation, showing the fluorescence enhancement factors from pH = 7 to pH = 5. Solvent: MeCN/PBS = 1 : 1(v/v). (e) Comparison of the HOMO and LUMO energy levels, S_0_–S_1_ excitation energies for Lyso1005 and Lyso1005-2H^+^. (f) Epifluorescence images of 4T1 cells, co-stained with Lyso880 (red, *λ*_ex_/*λ*_em_ = 808/850–1700 nm, 10 μM) and a commercial lysosome tracking dye LysoBrite™ NIR (green, *λ*_ex_/*λ*_em_ = 655/750–1000 nm, 50 nM). Co-localization results show the high Pearson coefficients of 0.92. (g) Epifluorescence images of 4T1 cells, co-stained with Lyso1005 (red, *λ*_ex_/*λ*_em_ = 808/850–1700 nm, 10 μM) and LysoBrite™ NIR (green, *λ*_ex_/*λ*_em_ = 655/750–1000 nm, 50 nM). Co-localization results show a high Pearson coefficient of 0.86.

To this end, we first developed a series of acidic pH-sensitive fluorescence probes for lysosome tracking. We created four dyes, Lyso855/Lyso880 and Lyso950/Lyso1005, by introducing the lysosome-targeting piperazine moieties^30,31^ at the C_7_/C_6_ position of the NIR-II dye skeletons (Flav^32^ and BTC^33^) (Fig. 1a; synthetic methods are shown in the ESI†). Their spectra–structure relationship is predictable (Fig. S1–4†), that is, the sulfur heteroatom and C_6_ electron-donating substitution afford a longer wavelength due to enhanced delocalization and intramolecular charge transfer (ICT) effect, respectively.^33,34^ All dyes show excellent photo-stability and chemical stability under physiological conditions (Fig. S15–19†). To screen the optimal lysosomal pH sensitive dye, we measured the absorption and fluorescence spectra of the four dyes in the pH range of 3.0–8.0. Fig. 1b shows their p*K*_a_ values centered around 5 based on the absorption changes (Fig. S1–4†), suitable for sensing the acidic environment (pH = 5.0–6.0) of lysosomes. Owing to the protonation of the piperazine groups, enhanced fluorescence of these dyes was observed at lower pH. In particular, Lyso880 and Lyso1005 with piperazine moieties substituted at the C_6_ position exhibited more than 4-fold fluorescence enhancement when the pH decreased from 7.0 to 5.0 (Fig. 1c and d).

To further elucidate the pH sensing mechanism, TF-DFT calculations were performed for all dyes at the B3LYP/6-311 G (d, p) level after geometry optimization (Fig. 1e). For Lyso1005, the π-electron distribution of HOMO on the piperazine groups is significantly delocalized onto the *p*-methoxy phenyl groups of LUMO upon photoexcitation. In comparison, protonated Lyso1005 (Lyso1005-2H^+^) shows little difference between the HOMO and LUMO, indicating that protonation of piperazine moieties suppresses intramolecular charge transfer.^34^ This results in enhanced fluorescence, especially in a polar solvent, as confirmed by a 3.5-fold enhancement of quantum yield (QY) in ethanol after adding trifluoroacetic acid (Table S1†). Similar results were obtained for C_6_ substituted Lyso880, but not significantly for C_7_ substituted Lyso855 and Lyso950 (Fig. S6–8 and Table S1†), consistent with their pH-sensitive fluorescence properties. Furthermore, the S_0_–S_1_ excitation energies slightly increased from Lyso1005 (1.67 eV) to Lyso1005-2H^+^ (1.71 eV), corresponding to the blue-shift spectra of Lyso1005-2H^+^ (Fig. 1e and Table S1†). Therefore, we reasoned that weakening the electron-donating ability of piperazine moieties by the negative inductive effect of protonated nitrogen is the main reason for pH sensitivity. To confirm this conclusion, we synthesized another model compound *boc*-Lyso1005 by replacing the methyl groups of piperazine with the electron-withdrawing *t*-butyloxy carbonyl groups (synthetic methods shown in the ESI†). The higher QY (0.13%) and blue-shifted absorption of *boc*-Lyso1005 compared with Lyso1005 further verify that enhancing the negative inductive effect of the piperazine aliphatic amine contributes positively to the photophysical properties of dyes (Table S1†).

We next co-stained A549 and 4T1 cells with these dyes and the commercial lysosomal tracker (LysoBrite™ NIR), respectively. In addition to no obvious cytotoxicity observed (Fig. S10†), the merged fluorescence images and co-localization results show overlapped dotted signals inside cells with high Pearson coefficient over 0.80 (Fig. 1f, g and S11–14†), suggesting that almost all dyes are capable of lysosomal tracking. Lyso1005 with the optimal fluorescence enhancement and longest wavelength was thus chosen for the following experiments.

### Preparation and characterization of the NIR-II cell endocytosis-activated fluorescence probe (CEAF-OMe)

**Fig. 2:**
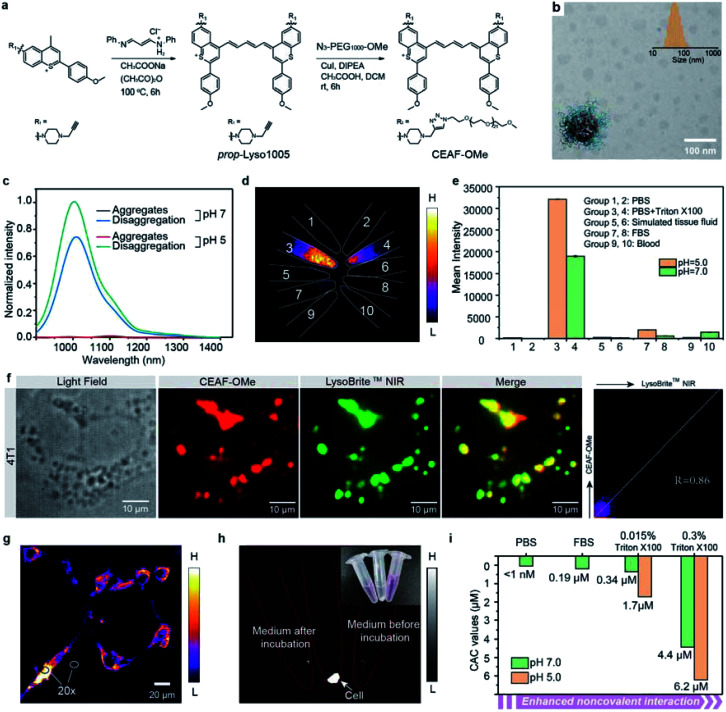
(a) The synthetic route of CEAF-OMe. (b) The TEM images of CEAF-OMe nanoaggregates. Inset shows the size distribution determined by dynamic light scattering. (c) Fluorescence spectra of CEAF-OMe at 808 nm excitation under various conditions. Black: pH 7.0 phosphate buffer; red: pH 5.0 phosphate buffer; blue: pH 7.0 phosphate buffer with the addition of 0.015 wt% Triton X-100; green: pH 5.0 phosphate buffer with the addition of 0.015 wt% Triton X-100. Triton X-100 was used to stimulate strong noncovalent interaction. (d) Fluorescence images of CEAF-OMe nanoaggregates (10 μL, 0.5 mg mL^−1^) added in 500 μL of different media under 940 nm laser irradiation. Group 1: PBS of pH = 5.0; group 2: PBS of pH = 7.0; group 3: PBS with 0.015% Triton X-100 of pH = 5.0; group 4: PBS with 0.015% Triton X-100 of pH = 7.0; group 5: simulated tissue fluid of pH = 5.0; group 6: simulated tissue fluid of pH = 7.0; group 7: FBS of pH = 5.0; group 8: FBS of pH = 7.0; group 9: blood of pH = 5.0; group 10: blood of pH = 7.0. (e) The signal intensities of all samples in (d). (f) Epifluorescence images of 4T1 cells, co-stained with CEAF-OMe nanoaggregates (red, *λ*_ex_/*λ*_em_ = 808/850–1700 nm, 10 μM) and LysoBrite™ NIR (green, *λ*_ex_/*λ*_em_ = 655/750–1000 nm, 50 nM). Co-localization results show a high Pearson coefficient of 0.86. (g). “No-wash” epifluorescence images of 4T1 cells, co-incubated with CEAF-OMe nanoaggregates for 12 h (*λ*_ex_/*λ*_em_ = 940/1200–1700 nm, 10 μM). Inset shows the ratio between the intracellular and extracellular signals. (h) Fluorescence images of the culture medium after incubation with cells, cells separated by centrifugation and culture medium before incubation with cells under 940 nm laser irradiation. (i) The determined CAC values of CEAF-OMe probes under various conditions showing that increased non-covalent interaction and acid pH accelerate disaggregation of the CEAF-OMe probes.

The non-specific binding trap of plasma proteins contributes greatly to the high background of small-molecule probes in blood and normal tissues.^17–19^ To reduce the non-specific binding effect, we synthesized the amphiphilic Lyso1005 (namely CEAF-OMe) by a click reaction between a propargyl-functionalized Lyso1005 (*prop*-Lyso1005) and two polyethylene glycol (PEG) chains of ∼1 kDa (N_3_–PEG_1000_–OCH_3_) (Fig. 2a). Maldi-TOF spectrum confirmed the bis-PEGylated structure (Fig. S59†). CEAF-OMe can form uniform spherical nanoaggregates in pH 7.4 phosphate buffer (PBS) with an average hydrodynamic diameter of ∼76 nm (Fig. 2b), which completely quenches the fluorescence due to the aggregation induced quenching (ACQ) effect. It has been demonstrated that surfactants such as Triton X-100 could be used for mimicking the non-covalent interaction between the molecules and lysosomal contents (*i.e.*, proteins, lipids and fatty acids).^35–37^ Notably, after adding 0.015 wt% Triton X-100 (Fig. 2c), significant fluorescence recovery (72-fold) can be observed, corresponding to the disaggregation-induced fluorescence activation. Similar results are also concluded with the addition of other surfactants (Fig. S20†). Notably, the fluorescence recovery cannot be achieved only by lowering the pH to 5.0, despite approaching the p*K*_a_ value (p*K*_a_ = 5.02, Fig. S5†). However, after further addition of Triton X-100, the fluorescence increased 1.5 times more than that under the condition of Triton X-100 + pH 7.4, realizing 108-fold enhancement in all compared with the quenched state. We then tested the stability of CEAF-OMe in various biological fluids, such as fetal bovine serum (FBS), blood, and simulated tissue fluid at both pH = 7.0 and pH = 5.0 (Fig. 2d and e). NIR-II fluorescence imaging showed that CEAF-OMe remained quenched in these fluids and only exhibited bright signals under the condition of Triton X-100, illustrating that CEAF-OMe has considerable stability to plasma proteins. This will allow low fluorescence background in blood vessels when using CEAF-OMe for *in vivo* imaging. However, when CEAF-OMe was co-incubated with 4T1 cells for 12 h, co-localization results showed that the signals of CEAF-OMe overlapped with that of LysoBrite™ NIR in cell lysosomes with a high Pearson coefficient of 0.86 (Fig. 2f), indicating the selective activation of CEAF in cell lysosomes. Besides, “no-wash” cell imaging showed distinct fluorescence activation inside the cells, while almost no fluorescence could be detected in the cell culture (Fig. 2g and h). The ratios of intracellular to extracellular signals reached up to 20 for 4T1 cells (Fig. 2g). These results evidenced that CEAF-OMe was mainly activated inside cells through the endocytosis mechanism.

### *In vitro* mechanism study of CEAF-OMe activation

To rationalize the above phenomena, we measured the concentration-dependent fluorescence of CEAF-OMe in the relevant media/conditions (Fig. 2i, S21 and 22†). The corresponding transition point can be recognized as the critical aggregation concentration (CAC), reflecting the disaggregation tendency of CEAF-OMe under different environmental conditions.^38^ CEAF-OMe has an undetectable ultralow critical aggregation concentration (CAC) below 1 nM in pH 7.4 PBS (Fig. S21a and b†)—an important characteristic suggesting potential tolerance toward blood dilution.^39^ The CAC increased to ∼341 nM in the presence of 0.015 wt% Triton X-100 due to enhanced noncovalent interaction, and exhibited positive correlation with Triton X-100 concentration (Fig. S22†). Remarkably, lowering the pH to 5.0 further increased the CAC value by 5 fold to ∼1700 nM, indicating the significant contribution of protonation to disaggregation. In comparison, the CAC in FBS solution (pH 7.4) was only ∼190 nM (Fig. S21c and d†), illustrating the weak influence of plasma proteins on CEAF-OMe, thus ensuring the dark background of CEAF-OMe nanoaggregates in blood circulation. Collectively, the results support a reasonable CEAF mechanism according to which the high activation of the probes relies on the synergy of disaggregation and protonation, which are controlled by the difference in noncovalent interactions and acid strength of lysosomes relative to an extracellular medium or blood.

### *In vivo* NIR-II tumor imaging and surgical guidance with CEAF-OMe

**Fig. 3:**
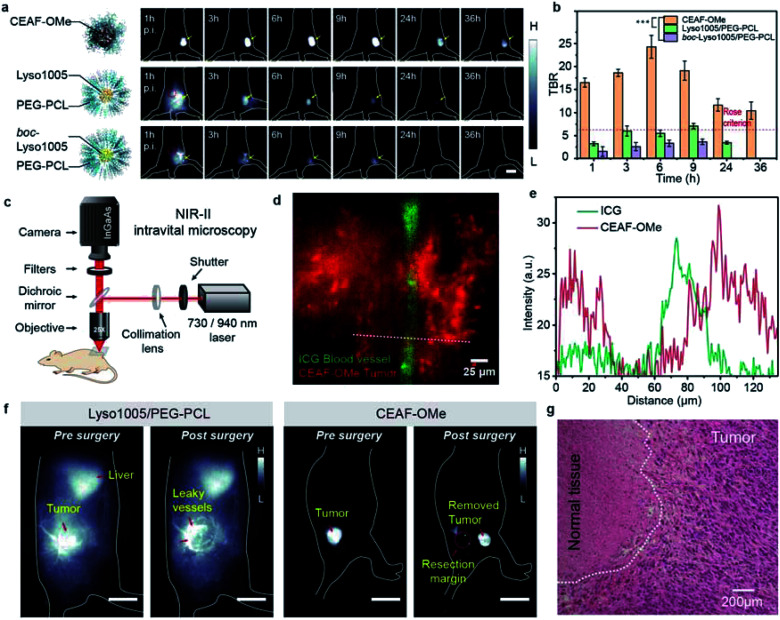
(a) *In vivo* NIR-II fluorescence images of mice bearing CT26 tumors at different time points after intravenous injection with CEAF-OMe nanoaggregates, Lyso1005/PEG–PCL micelles, and *boc*-Lyso1005/PEG–PCL micelles. Scale bar, 2.5 cm. (b) TBRs obtained for CEAF-OMe (50 μM, 200 μL), Lyso1005/PEG–PCL (50 μM, 200 μL) and *boc*-Lyso1005/PEG–PCL (50 μM, 200 μL) over the time course of 36 h (*n* = 3 mice); *p* values were analyzed between CEAF-OMe treated and Lyso1005/PEG–PCL micelles treated mice, CEAF-OMe treated and *boc*-Lyso1005/PEG–PCL micelles treated mice at all time points starting from 1 h by Student's two-sided *t* test (***P < 0.001). Red circles in (a) denote the background. (c) Schematic diagram of the home-built *in vivo* NIR-II epifluorescence microscope. (d) Two-color NIR-II intravital epifluorescence image of CT26 tumor stroma and vessel. Red: CEAF-OMe (50 μM, 200 μL), *λ*_ex_/*λ*_em_ = 940/1200–1700 nm; green: ICG (50 μM, 50 μL), *λ*_ex_/*λ*_em_ = 730/1000–1700 nm. (e) Cross-sectional intensity profile along the white dashed bar in (d). (f) Pre- and post-surgery fluorescence images of mouse bearing CT26 tumor using Lyso1005/PEG–PCL micelles and CEAF-OMe as contrast agents (50 μM, 200 μL), respectively. Scale bar, 2 cm. (g) H&E staining image of resected tumor samples from mouse injected with CEAF-OMe nanoaggregates after surgery. Scale bar: 200 μm. Repeated 3 times in independent experiments.

Encouraged by the above results, we applied CEAF-OMe for *in vivo* tumor imaging. We reasoned that tumor angiogenesis may induce CEAF-OMe nanoaggregates to exploit passive targeting for specific endocytosis activation by tumor cells. As a comparison, two “always-on” nanoprobes (Lyso1005/PEG–PCL and *boc*-Lyso1005/PEG–PCL) with similar hydrodynamic sizes to CEAF-OMe (Fig. S23†) were chosen as control groups, which were polyethylene glycol-*b*-polycaprolactone (PEG–PCL) micelles encapsulating pH sensitive Lyso1005 and non-sensitive *boc*-Lyso1005, respectively (Fig. S24†). *In vivo* whole-body NIR-II fluorescence imaging was performed in nude mice bearing subcutaneous CT26 tumors (*n* = 3 for each group, Fig. 3a and S25†) immediately after tail-vein injection of these probes. As shown in Fig. 3a, CEAF-OMe could clearly resolve the tumor margin after 1 h post-injection (p.i.), while the two control groups were unable to distinguish tumors due to the strong background signals from skin and blood vessels. Notably, the TBRs of the CEAF-OMe group maintained high values above 10 within 36 h p.i. (Fig. 3b), significantly higher than the Rose criterion (which states that a TBR of 5 is needed to distinguish image features with 100% certainty).^40^ However, in the control groups, only Lyso1005/PEG–PCL showed a signal window that met the Rose criterion and lasted for 6 h. Therefore, the results demonstrate that the CEAF strategy combined with lysosomal tracking property endows probes with long retention time and high TBR in tumor, favorable for stable image-guided surgery.

To further confirm the specificity of CEAF-OMe, NIR-II intravital microscopic imaging was performed after 3 h p.i. of probes (Fig. 3c). A two-color epifluorescence image was obtained by using indocyanine green (ICG) as a vascular contrast agent simultaneously (Fig. 3d). It could be seen that the fluorescence signals of CEAF-OMe were specifically activated in tumor cells, and had little overlap with the ICG signals in the blood vessel (Fig. 3e), which further confirmed the low background of CEAF-OMe in blood. This property is particularly useful for accurate tumor-removal surgery as it can avoid the serious fluorescence contamination caused by intraoperative bleeding.^41,42^ To demonstrate this expectation, we conducted the fluorescence-guided surgery, where CEAF-OMe allowed a fast-acting and complete resection of the tumor within 3 h p.i., as confirmed by the tissue hematoxylin and eosin (H&E) staining results (Fig. 3f, g and Video S1†). However, the control group injected with Lyso1005/PEG–PCL showed strong fluorescence contamination from leaky blood, hindering the subsequent operations (Fig. 3f and Video S2†).

### Preparation and characterization of the NIR-II functional CEAF-NHS and α_v_β_3_-specific CEAF-RGD

**Fig. 4:**
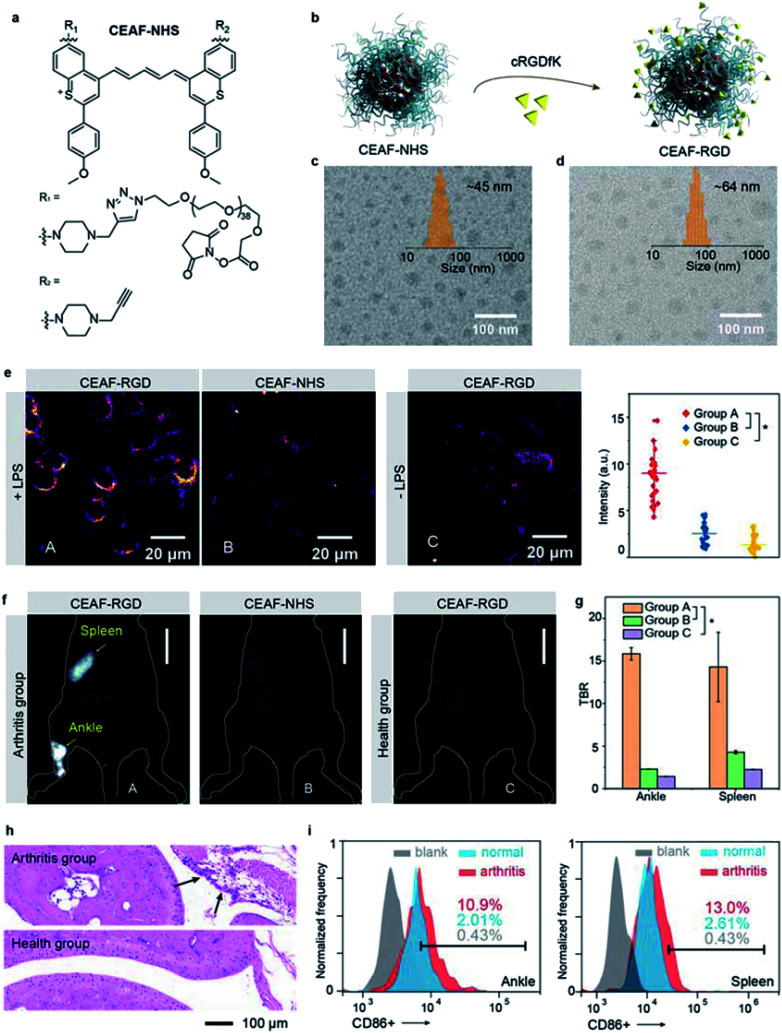
(a) Chemical structure of CEAF-NHS. (b) Schematic showing the preparation of CEAF-RGD. The TEM images of CEAF-NHS (c) and CEAF-RGD (d) nanoaggregates. Inset shows their size distributions determined by dynamic light scattering. (e) “No-wash” epifluorescence images of RAW264.7 cells and the corresponding cell signal statistics after co-incubating with probes (10 μM) for 2 h (*λ*_ex_/*λ*_em_ = 940/1200–1700 nm). Group A: cells induced by LPS and co-incubated with CEAF-RGD; group B: cells induced by LPS and co-incubated with CEAF-NHS blocking with the glycine; group C: cells co-incubated with CEAF-RGD. *p* values were analyzed between group A and group B, group A and group C by Student's two-sided *t* test (*P < 0.05). (f) *In vivo* NIR-II fluorescence images of arthritis mice injected with CEAF-RGD (group A, 50 μM, 200 μL) and CEAF-NHS blocking with glycine (group B, 50 μM, 200 μL), and normal mice injected with CEAF-RGD (group C, 50 μM, 200 μL), scale bar: 10 mm. (g) TBRs of mice whose ankle joints and spleens are injured (*n* = 3 mice for each group), red circles in (f) denote the background. *p* values were analyzed between group A and group B, group A and group C at ankle and spleen regions by Student's two-sided *t* test (*P < 0.05). (h) H&E staining of arthritis and normal articular cavity. (i) Quantification of M1 microphages in injured articular cavity (left) and spleen (right) of arthritis and normal mouse, respectively.

Besides tumor imaging based on passive targeting, equipping CEAF probes with affinity ligands will enable cell-specific diagnostics.^43,44^ Traumatic arthritis (TA) caused by intense external force is an acute inflammatory disease and its early recognition and treatment is essential for achieving effective therapeutic outcome.^45^ It was reported that the inflammatory response after TA involves the recruitment of M1 macrophages with highly overexpressed integrin α_v_β_3_ to the articular cavity, which can be a potential biomarker for early diagnosis of TA.^46^ To demonstrate the versatility of the CEAF strategy, we synthesized a functionalized CEAF probe with terminal *N*-hydroxysuccinimide (NHS) active ester group (namely CEAF-NHS, Fig. 4a). Similarly, CEAF-NHS forms uniform spherical nanoaggregates in pH 7.4 phosphate buffer with quenched fluorescence, which can be facilely modified with active-targeting ligands through an amidation reaction (Fig. 4b). In this case for TA diagnosis, the selective ligands for α_v_β_3_ integrin, cRGDfK (cyclo Arg–Gly–Asp–D–Tyr–Lys), were conjugated to the surface of CEAF-NHS nanoaggregates, which created a targetable CEAF probe CEAF-RGD for specifically recognizing M1 macrophages.^47–49^ Successful modification was confirmed by the increased hydrodynamic diameter of nanoaggregates from ∼45 to ∼64 nm (Fig. 4c and d).

To investigate the specificity of probes for M1 macrophages, CEAF-RGD were incubated with RAW264.7 (+LPS) and RAW264.7 (−LPS) cells for 2 h, where lipopolysaccharide (LPS) serves as an inducer to polarize the RAW264.7 cells toward the M1 phenotype.^50^ As a control, CEAF-NHS with the active end-group blocked by glycine were further incubated with RAW264.7 (+LPS) for 2 h as well (Fig. 4e). “No-wash” cell imaging results showed that RAW264.7 (+LPS) cells incubated with CEAF-RGD exhibited 3.4- to 5.6-fold higher fluorescence than the cells of the other two groups, indicating that CEAF-RGD is capable of specific fluorescence activation to M1 macrophages through the receptor-mediated endocytosis.

### *In vivo* NIR-II fluorescence diagnosis of arthritis with CEAF-RGD

Next, CEAF-RGD and glycine-blocked CEAF-NHS were intravenously injected into mouse models with TA on one leg, respectively, followed by *in vivo* NIR-II fluorescence imaging (Fig. 4f and S26†). In the TA mice using CEAF-RGD, the injured joint could be specifically resolved with a high TBR of ∼15.8 (Fig. 4f and g). Almost no signal was observed with CEAF-NHS, while a similar result was obtained in normal mice injected with CEAF-RGD (Fig. 4f). These results demonstrate the superior specificity of CEAF-RGD for the TA diagnosis. Interestingly, spleen could also be clearly distinguished with a high TBR of ∼14.3 in the TA mice using CEAF-RGD (Fig. 4f and g). This is consistent with the general understanding that massive expansion of mononuclear cells occurs in TA spleen and articular cavity.^46^ To further support the imaging results, we carried out the histological and flow cytometry analysis on the TA and normal mice, respectively. The results showed that macrophage infiltration (black arrow in Fig. 4h) occurred in the TA articular cavity, and increased number of M1 macrophages was observed in both the TA articular cavity and spleen (Fig. 4i and S27†), thus confirming the observation of *in vivo* NIR-II fluorescence imaging.

## Conclusions

In conclusion, we have developed CEAF probes through the creation and hydrophilic polymer modification of four NIR-II lysosome tracking dyes, and demonstrated their utilities for enhancing the effectiveness of tumor resections during fluorescence-guided surgery, as well as for cell-specific diagnosis of traumatic arthritis. The mechanism of action for CEAF probes is based on the disaggregation and protonation of probe aggregates in targeting cell lysosomes, which eliminates the fluorescence background generated in blood circulation and thus significantly enhances *in vivo* imaging sensitivity and contrast. Compared with activatable probes established on single physiological biomarkers, the NIR-II CEAF probes equipped with various cell-surface affinity ligands not only will provide a general and robust solution for diverse disease diagnostics, but also enable high-contrast cell-specific imaging *in vivo*.

## Ethical statement

All animal procedures were performed in accordance with the guidelines of the Institutional Animal Care and Use Committee of Fudan University, in agreement with the institutional guidelines for animal handling. All of the animal experiments were authorized by the Shanghai Science and Technology Committee.

## Data availability

All experimental or computational data associated with this article have been provided in the supporting information and supporting video files.

## Author contributions

The manuscript was written through contributions of all authors. All authors have given approval to the final version of the manuscript.

## Conflicts of interest

The authors declare no competing financial interest.

## Supplementary Material

SC-012-D1SC02763H-s001

SC-012-D1SC02763H-s002

SC-012-D1SC02763H-s003
