# Supplementary material for: NIR-II cell endocytosis-activated fluorescent probes for in vivo high-contrast bioimaging diagnostics
Source: Chem Sci. 2021 Jun 30;12(31):10474–82. doi: 10.1039/d1sc02763h (PMC8356747; doi:10.1039/d1sc02763h)
Supplement: SC-012-D1SC02763H-s001 [file SC-012-D1SC02763H-s001.pdf]

Electronic Supplementary Material (ESI) for Chemical Science.
This journal is © The Royal Society of Chemistry 2021

Part A: Supplementary Experimental Section

Materials

Analytical grade solvents including petroleum ether, dichloromethane, and ethyl acetate were purchased from Titan Scientific. N_3-PEG_{1000}-OCH_3 and DSPE-PEG2000 were purchased from Ponsure Biotechnology. N_3-PEG_{2000}-NHS was purchased from SINOPEG. cRGDfK peptide (Arg-Gly-Asp-D-Tyr-Lys) was purchased from Chinapeptides. Dulbecco's modified eagle medium (DMEM) cell culture was purchased from Gibco. Fetal bovine serum (FBS) was purchased from ExCell. Trypsin (0.25%) and penicillin-streptomycin solution (100X) were purchased from MesGen Biotechnology. CD45 (APC, 103111), F4/80 (FITC, 123108), CD11b (PerCP, 101229), CD86 (PE, 105105) were purchased from Biolegend. All other chemicals were purchased from TCI and J&K without further purification unless otherwise noted.

Synthetic procedures

Preparation of Lyso880

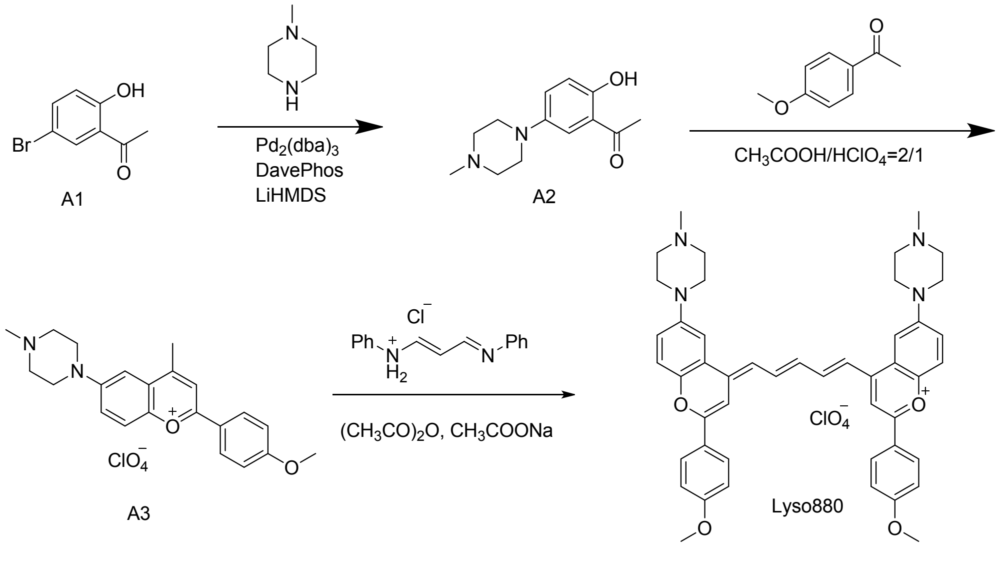

Scheme S1. Complete synthetic route of **Lyso880**.

Synthesis of compound A2. Under the protection of nitrogen, compound A1 (10 g, 0.0465 mol, 1 eq), $Pd_2(dba)_3$ (1.1 g, 0.0012 mol, 0.025 eq), DavePhos (0.91 g, 0.0023 mol, 0.05 eq), and LiHMDS (163 mL, 0.1627 mol, 3.5 eq) were mixed in dry dioxane, following the addition of methylpiperazine (26 mL, 0.233 mol, 5 eq). The reaction was stirred at 80 ℃ for 2 h. After cooling to room temperature, the mixture

was filtered, and the filtrate was evaporated. The crude product was further purified by a flash column chromatography [silica gel, PE/DCM = 1/1 to 0/1, v/v, then EA/DCM = 1/10, v/v] to obtain compound A2 (9.3 g, yield: 85%). ^{1}H NMR (400 MHz, CD_3OD) δ 8.19 (d, J = 2.1 Hz, 1H), 7.83 (s, 1H), 7.66 (dd, J = 7.1, 1.8 Hz, 3H), 7.25 (s, 1H), 7.04 – 6.99 (m, 2H), 3.88 (s, 3H), 3.84 (s, 4H), 3.46 – 3.34 (m, 4H), 1.50 (s, 9H). ^{13}C NMR (101 MHz, CD_3OD) δ 157.47, 144.12, 128.51, 120.30, 119.17, 119.01, 55.14, 50.12, 44.60, 26.60. Maldi-Tof/Tof-MS: calcd for $C_{13}H_{18}N_2O_2^+$ $[M]^+$, 234.1368; Found, 234.1327. $[M]^+$.

Synthesis of compound A3. Under the protection of nitrogen, compound A2 (1 g, 0.0043 mmol, 1 eq) and compound 4-methoxyacetophenone (641 mg, 0.0043 mmol, 1 eq) were added to the mixed solvent of acetic acid and perchloric acid (v/v=2/1). The reaction was refluxed for 16 h. After cooling to room temperature, ethyl acetate was added until there was a large amount of precipitation. The crude product was collected by filtration and dried to obtain the compound A3 (1.2 g, yield: 60%) without further purification. Maldi-Tof/Tof-MS: calcd for $C_{22}H_{25}N_2O_2^+[M]^+$, 349.1911; Found, 349.1835. $[M]^+$.

Synthesis of Lyso880. To a suspension of N-[3-(phenylamino)allylidene]aniline monohydrochloride (46.8 mg, 1.81 mmol, 1 eq) in Ac_2O (2 mL) was added NaOAc (48.7 mg, 4.89 mmol, 2.7 eq). The mixture was stirred at ambient temperature for 30 min until color fading. Compound A3 (162 mg, 3.62 mmol, 2 eq) was added to the resulting clear solution and subsequently heated at 100 ℃ under nitrogen for 6 h. After cooling to room temperature, the mixture was evaporated and purified by a flash column chromatography [C18, MeCN (0.1%TFA) / H_2O (0.1%TFA) = 20/100, v/v] to give Lyso880 (603 mg, yield: 40%). ^{1}H NMR (400 MHz, DMSO) δ 8.65 (t, J = 13.1 Hz, 2H), 8.31 (d, J = 8.9 Hz, 4H), 8.12 (s, 2H), 7.82 (d, J = 9.2 Hz, 2H), 7.77 – 7.59 (m, 4H), 7.46 (d, J = 14.2 Hz, 2H), 7.22 (d, J = 9.0 Hz, 4H), 7.12 (t, J = 12.5 Hz, 1H), 4.15 (d, J = 13.3 Hz, 4H), 3.93 (s, 6H), 3.63 (d, J = 11.6 Hz, 4H), 3.22 (dt, J = 22.4, 11.5 Hz, 8H), 2.93 (s, 6H). Maldi-Tof/Tof-MS: calcd for $C_{47}H_{49}N_4O_4^+$ $[M]^+$, 733.3748; Found, 733.3004. $[M]^+$.

Preparation of Lyso1005

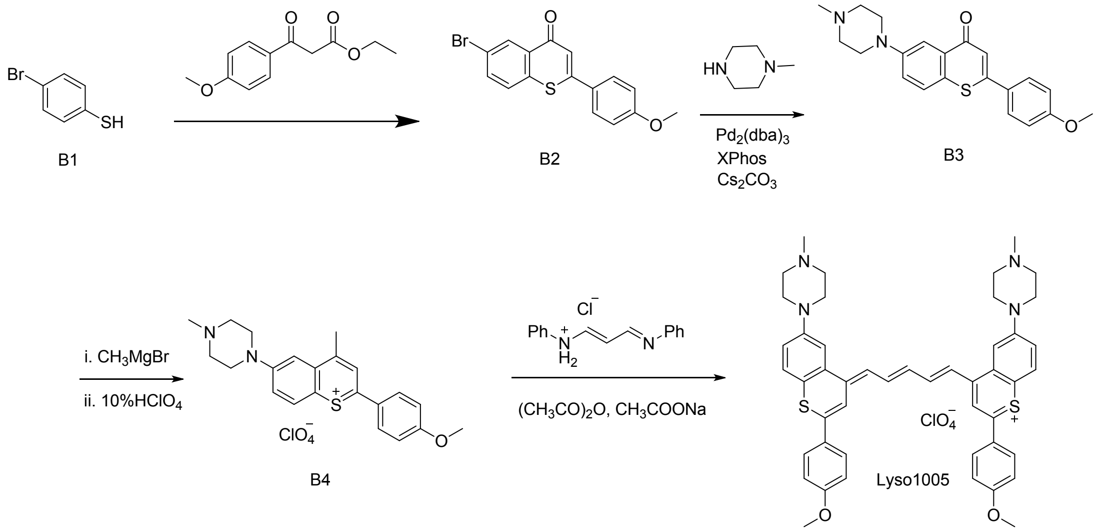

Scheme S2. Complete synthetic route of **Lyso1005**.

Synthesis of compound B2. Compound B2 was synthesized according to a reported method.[1] ^{1}H NMR (400 MHz, $CDCl_3$) δ 8.55 (d, J = 8.0 Hz, 1H), 7.66 (m, 8.3 Hz, 4H), 7.57 (t, J = 7.4 Hz, 1H), 7.34 (s, 1H), 7.02 (d, J = 8.7 Hz, 2H), 3.88 (s, 3H).

Synthesis of compound B3. A tube was charged with compound B2 (1 g, 0.00288 mmol, 1 eq), $Pd_2(dba)_3$ (0.264 g, 0.1 eq), XPhos (0.137 g, 0.1 eq), and Cs_2CO_3 (2.346 g, 0.0072 mmol, 2.5 eq). The tube was sealed and evacuated/backfilled with nitrogen (3x). Anhydrous dioxane (10 mL) was added, following the addition of methylpiperazine (1.6 mL, 0.0144 mol, 5 eq). The reaction was stirred at 100 °C for 18 h. After cooling to room temperature, the mixture was filtered, and the filtrate was evaporated. The crude product was further purified by a flash column chromatography [silica gel, PE/DCM = 1/1 to 0/1, v/v, then EA/DCM = 1/10, v/v] to give compound B3 (896 mg, yield: 85%). ^{1}H NMR (400 MHz, $CDCl_3$) δ 7.96 (d, *J* = 2.7 Hz, 1H), 7.65 (d, *J* = 8.7 Hz, 2H), 7.54 (d, *J* = 8.9 Hz, 1H), 7.29 (d, *J* = 2.7 Hz, 1H), 7.27 (s, 1H), 7.19 (s, 1H), 7.00 (d, *J* = 8.7 Hz, 2H), 3.87 (s, 3H), 3.43 – 3.32 (m, 4H), 2.69 – 2.56 (m, 5H), 2.38 (s, 3H). ^{13}C NMR (101 MHz, $CDCl_3$) δ 181.07, 161.89, 152.78, 150.92, 131.96, 129.34, 128.52, 128.47, 127.89, 127.51, 121.61, 121.52, 121.05, 114.85, 114.79, 112.50, 77.59, 77.27, 76.95, 55.77, 55.66, 55.04, 48.59, 46.34, 46.30. Maldi-Tof/Tof-MS: calcd for $C_{21}H_{22}N_2O_2S^+$ $[M]^+$, 366.1402; Found, 366.1505. $[M]^+$.

Synthesis of compound B4. Under the protection of nitrogen, compound B3 (100 mg, 0.273 mmol, 1 eq) was dissolved in 5 mL of dry tetrahydrofuran. Methylmagnesium bromide (1 mol L^{-1}, 820 μL, 0.820 mmol, 3 eq) was added, and the reaction was stirred at room temperature for 2 h. The reaction was quenched by adding 10% perchloric acid to form a precipitate, which was filtered to obtain compound B4 (123 mg, yield: 97%) without further purification. Maldi-Tof/Tof-MS: calcd for $C_{22}H_{25}N_2OS^+$ $[M]^+$, 365.1682; Found, 365.1862. $[M]^+$.

Synthesis of Lyso1005. Lyso1005 was synthesized by the similar procedure as Lyso880 (Yield: 40%). ^{1}H NMR (400 MHz, DMSO) δ 8.50 (t, J = 12.5 Hz, 2H), 8.31 (s, 2H), 7.96 (d, J = 8.8 Hz, 5H), 7.80 (d, J = 0.8 Hz, 2H), 7.65 (t, J = 12.9 Hz, 4H), 7.29 (t, J = 13.7 Hz, 2H), 7.18 (d, J = 8.8 Hz, 4H), 4.24 (d, J = 13.3 Hz, 4H), 3.88 (s, 6H), 3.59 (d, J = 10.0 Hz, 4H), 3.26 (m, 8H), 2.54 (s, 6H). Maldi-Tof/Tof-MS: calcd for $C_{47}H_{49}N_4O_2S_2^+$ $[M]^+$, 765.3291; Found, 765.3478. $[M]^+$.

Preparation of Lyso855

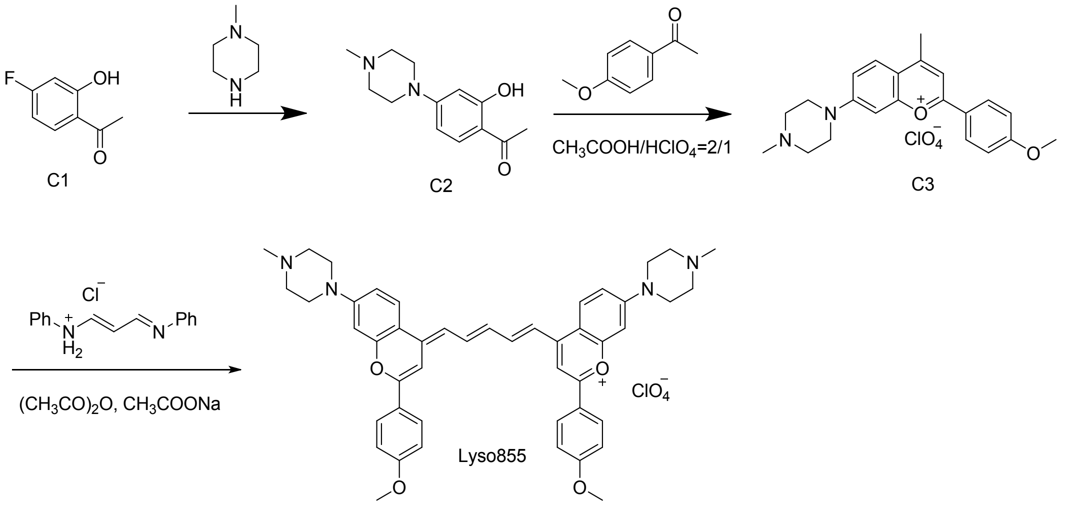

Scheme S3. Complete synthetic route of **Lyso855**.

Synthesis of C2. Compound C1 (5.3 g, 0.034 mol) was dissolved in methylpiperazine (6 mL) and the reaction was stirred at 130℃ for 5 h. After cooling to room temperature, the mixture was evaporated and the crude product was further purified by a flash column chromatography [silica gel, PE/DCM = 1/1 to 0/1, v/v] to obtain compound C2 (7.6 g, yield: 95%). ^{1}H NMR (400 MHz, $CDCl_3$) δ 8.19 (d, J = 2.1 Hz, 1H), 7.83 (s, 1H), 7.66 (dd, J = 7.1, 1.8 Hz, 3H), 7.25 (s, 1H), 7.04 – 6.99 (m, 2H), 3.88 (s, 3H), 3.84

(s, 4H), 3.46 – 3.34 (m, 4H), 1.50 (s, 9H). ^{13}C NMR (101 MHz, $CDCl_3$) δ 201.34, 165.11, 156.39, 132.54, 111.71, 105.67, 100.63, 54.41, 46.76, 46.11, 26.03. Maldi-Tof/Tof-MS: calcd for $C_{13}H_{18}N_2O_2^+$ [M]$^+$, 234.1368; Found, 234.1156. [M]$^+$.

Synthesis of C3. Compound C3 was synthesized by the similar procedure as compound A3 (Yield: 65%). Maldi-Tof/Tof-MS: calcd for $C_{22}H_{25}N_2O_2^+$[M]$^+$, 349.1911; Found, 349.1627. [M]$^+$.

Synthesis of Lyso855. Lyso855 was synthesized by the similar procedure as Lyso880 (Yield: 58%). ^{1}H NMR (400 MHz, DMSO) δ 8.43 (t, J = 12.0 Hz, 2H), 8.20 (d, J = 7.1 Hz, 5H), 7.84 (s, 2H), 7.58 (d, J = 29.3 Hz, 2H), 7.22 (dd, J = 33.8, 10.0 Hz, 8H), 6.95 (dd, J = 32.6, 20.6 Hz, 2H), 4.28 (s, 4H), 3.90 (s, 6H), 3.57 (s, 4H), 3.34 (s, 4H), 3.19 (s, 4H), 2.88 (s, 6H). Maldi-Tof/Tof-MS: calcd for $C_{47}H_{49}N_4O_4^+$ [M]$^+$, 733.3748; Found, 733.3001. [M]$^+$.

Preparation of Lyso950

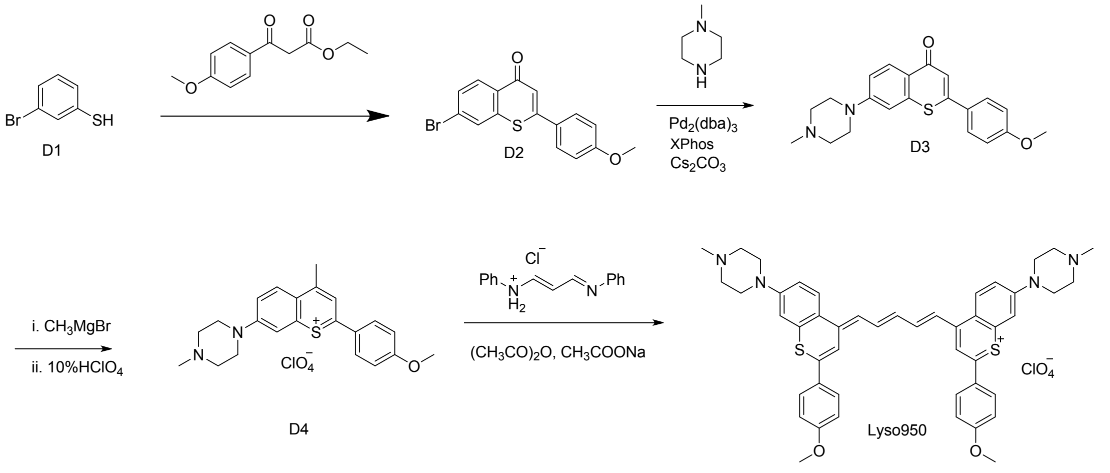

Scheme S4. Complete synthetic route of **Lyso950**.

Synthesis of D2. Compound D2 was synthesized according to a reported method.[2] ^{1}H NMR (400 MHz, CDC13) δ 8.37 (d, J = 8.6 Hz, 1H), 7.80 (s, 1H), 7.64 (d, J = 8.5 Hz, 2H), 7.26 (s, 1H), 7.20 (s, 1H), 7.01 (d, J = 8.7 Hz, 2H), 3.88 (s, 3H); ^{13}C NMR (100 MHz, CDC13) δ 180.3, 162.2, 152.9, 139.3, 135.4, 131.3, 130.3, 129.6, 128.8, 128.5, 126.8, 122.2, 114.9, 55.7; Maldi-Tof/Tof-MS: calcd for $C_{16}H_{12}BrO_2SH^+$ [M+H]$^+$, 346.9741; Found, 347.0120. [M+H]$^+$.

Synthesis of D3. Compound D3 was synthesized by the similar procedure as compound B3 (Yield: 85%). ^{1}H NMR (400 MHz, CD_3OD) δ 8.19 (d, *J* = 2.1 Hz, 1H), 7.83 (s, 1H), 7.66 (dd, *J* = 7.1, 1.8 Hz, 3H), 7.25 (s, 1H), 7.04 – 6.99 (m, 2H), 3.88 (s, 3H), 3.84 (s, 4H), 3.46 – 3.34 (m, 4H), 1.50 (s, 9H). ^{13}C NMR (101 MHz, CD_3OD) δ 192.67, 180.97, 162.31, 153.42, 152.67, 140.72, 128.93, 128.55, 128.00, 121.30, 120.76, 115.56, 114.58, 108.49, 54.77, 54.28, 46.39, 44.73. Maldi-Tof/Tof-MS: calcd for $C_{21}H_{22}N_2O_2S^+$ [M]$^+$, 366.1402; Found, 366.1304. [M]$^+$.

Synthesis of D4. Compound D4 was synthesized by the similar procedure as compound B4 (Yield: 97%). Maldi-Tof/Tof-MS: calcd for $C_{22}H_{25}N_2OS^+$ [M]$^+$, 365.1682; Found, 365.1862. [M]$^+$.

Synthesis of Lyso950. Lyso950 was synthesized by the similar procedure as Lyso1005 (Yield: 58%). ^{1}H NMR (400 MHz, DMSO) δ 8.44 (d, *J* = 9.7 Hz, 2H), 8.35 (t, *J* = 12.8 Hz, 2H), 8.07 (s, 2H), 7.87 (d, *J* = 8.8 Hz, 4H), 7.60 – 7.36 (m, 6H), 7.16 (d, *J* = 8.9 Hz, 4H), 7.06 (t, *J* = 13.1 Hz, 1H), 4.29 (d, *J* = 14.5 Hz, 4H), 3.86 (s, 6H), 3.57 (d, *J* = 11.8 Hz, 4H), 3.39 – 3.05 (m, 8H), 2.87 (s, 6H). Maldi-Tof/Tof-MS: calcd for $C_{47}H_{49}N_4O_2S_2^+$ [M]$^+$, 765.3291; Found, 765.2410. [M]$^+$.

Preparation of *prop*-Lyso1005

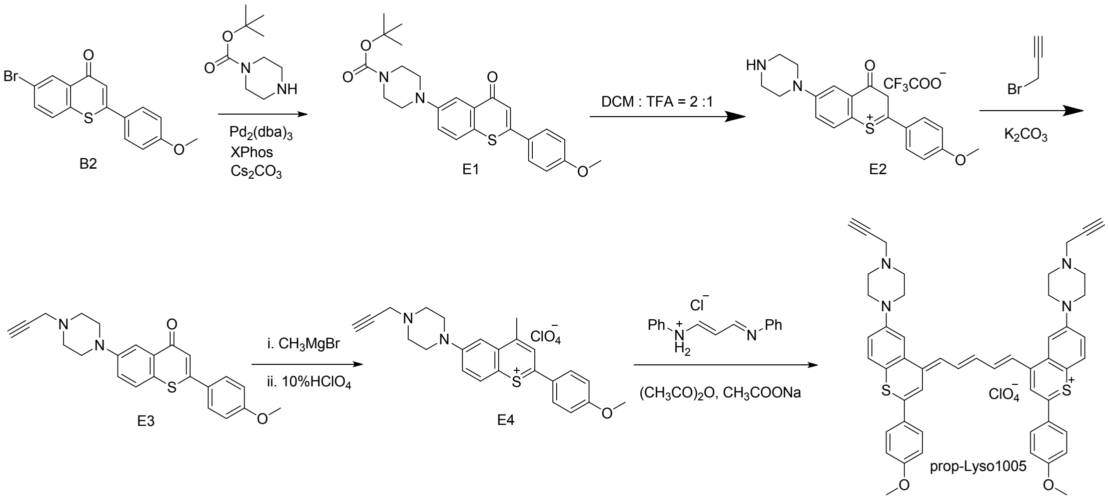

Scheme S5. Complete synthetic route of ***prop*-Lyso1005**.

Synthesis of E1. Compound E1 was synthesized by the similar procedure as compound B3 (Yield: 85%). ^{1}H NMR (400 MHz, $CDCl_3$) δ 8.19 (d, *J* = 2.1 Hz, 1H), 7.83 (s, 1H), 7.66 (dd, *J* = 7.1, 1.8 Hz, 3H), 7.25 (s, 1H), 7.04 – 6.99 (m, 2H), 3.88 (s, 3H), 3.84 (s, 4H), 3.46 – 3.34 (m, 4H), 1.50 (s, 9H). ^{13}C NMR (101

MHz, $CDCl_3$) δ 180.90, 161.97, 154.88, 152.94, 132.00, 129.23, 128.54, 127.70, 121.85, 121.65, 121.57, 114.83, 113.32, 80.39, 77.58, 77.26, 76.94, 55.78, 55.68, 49.19, 28.67, 28.63. Maldi-Tof/Tof-MS: calcd for $C_{25}H_{29}N_2O_4S^+$ $[M+H]^+$, 453.1770; Found, 453.1832. $[M+H]^+$.

Synthesis of E2. Compound E1 (1.13 g, 0.0025 mol) was dissolved in the mixed solvent of dichloromethane and trifluoroacetic acid (V/V=2/1), and the reaction was stirred under ice bath for 4 h. After the reaction is completed, the mixture was evaporated to obtain the compound E2 without further purification (1.1g, yield: 99%).

Synthesis of E3. Compound E2 (1.3 g, 0.0028 mol, 1 eq), propargyl bromide (1.25 g, 0.0084 mol, 3 eq) and potassium carbonate (1.13 g, 0.0084 mol, 3 eq) were dissolved in acetonitrile. The mixture was refluxed for 16 h. After cooling to room temperature, the mixture was filtered, and the filtrate was evaporated. The crude product was further purified by a flash column chromatography [silica gel, PE/EA = 1/0 to 1/1, v/v] to obtain compound E3 (366 mg, yield: 34%). ^1H NMR (400 MHz, $CDCl_3$) δ 7.97 (d, J = 2.7 Hz, 1H), 7.65 (d, J = 8.8 Hz, 2H), 7.54 (d, J = 8.9 Hz, 1H), 7.28 (dd, J = 8.0, 3.7 Hz, 2H), 7.19 (s, 1H), 7.00 (d, J = 8.8 Hz, 2H), 3.87 (s, 3H), 3.46 – 3.30 (m, 6H), 2.83 – 2.72 (m, 4H), 2.32 (t, J = 2.2 Hz, 1H). ^{13}C NMR (101 MHz, $CDCl_3$) δ 181.00, 161.90, 152.74, 150.83, 131.96, 129.29, 128.49, 127.99, 127.53, 121.58, 121.50, 121.18, 114.84, 112.56, 78.47, 77.62, 77.30, 76.98, 73.93, 55.75, 55.64, 51.74, 48.54, 47.07. Maldi-Tof/Tof-MS: calcd for $C_{23}H_{23}N_2O_2S^+$ $[M+H]^+$, 391.1402; Found, 391.1518. $[M+H]^+$.

Synthesis of E4. Compound E4 was synthesized by the similar procedure as compound B4 without further purification (Yield: 97%). Maldi-Tof/Tof-MS: calcd for $C_{24}H_{25}N_2OS^+$ $[M]^+$, 389.1682; Found, 389.1615. $[M]^+$.

Synthesis of prop-Lyso1005. *prop*-Lyso1005 was synthesized by the similar procedure as Lyso1005 (Yield: 40%). ^1H NMR (400 MHz, DMSO) δ 8.53 (dd, J = 22.2, 9.4 Hz, 2H), 8.34 (s, 2H), 7.99 (dd, J = 9.0, 3.0 Hz, 5H), 7.84 (s, 2H), 7.67 (t, J = 10.9 Hz, 4H), 7.30 (t, J = 12.5 Hz, 2H), 7.21 (d, J = 8.9 Hz, 4H), 7.13 (d, J = 8.9 Hz, 1H), 4.31 (d, J = 2.2 Hz, 4H), 3.95 (t, J = 2.4 Hz, 2H), 3.91 (s, 6H), 3.53 (s, 16H). Maldi-Tof/Tof-MS: calcd for $C_{51}H_{49}N_4O_2S_2^+$ $[M]^+$, 813.3291; Found, 813.3884. $[M]^+$.

Preparation of ***boc*-Lyso1005**

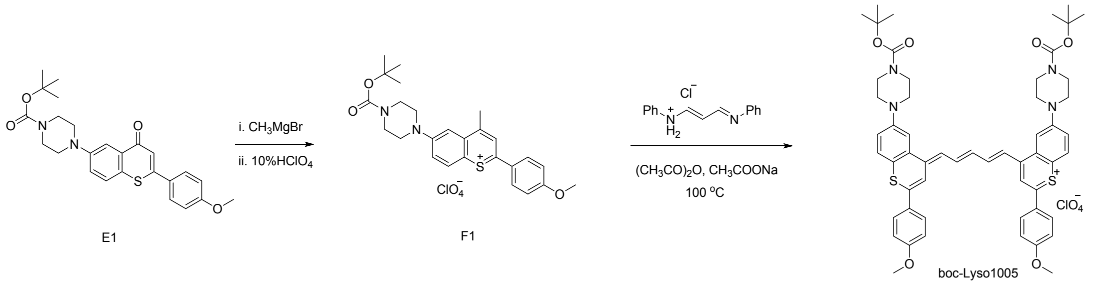

Scheme S6. Complete synthetic route of ***boc*-Lyso1005**.

Synthesis of F1. Compound F1 was synthesized by the similar procedure as compound B4 without further purification (Yield: 97%). Maldi-Tof/Tof-MS: calcd for $C_{26}H_{31}N_2O_3S^+$ [M]$^+$, 451.2050; Found, 451.1700. [M]$^+$.

Synthesis of boc-Lyso1005. *boc*-Lyso1005 was synthesized by the similar procedure as Lyso1005 (Yield: 40%). ^{1}H NMR (400 MHz, DMSO) δ 8.34 (s, 2H), 8.14 (s, 2H), 7.80 (dd, *J* = 14.6, 9.6 Hz, 7H), 7.51 (d, *J* = 28.0 Hz, 6H), 7.16 (d, *J* = 4.6 Hz, 1H), 7.10 (d, *J* = 8.5 Hz, 4H), 3.86 (s, 6H), 3.51 (s, 8H), 3.36 (s, 9H), 1.44 (s, 18H). Maldi-Tof/Tof-MS: calcd for $C_{55}H_{61}N_4O_6S_2^+$ [M]$^+$, 937.4027; Found, 937.2924. [M]$^+$.

Synthesis of CEAF-OMe. *prop*-Lyso1005 (50 mg, 0.055 mmol, 1 eq) and N_3-PEG$_{1000}$-OCH_3 (274 mg, 0.274 mmol, 5 eq) were dissolved in 10 mL of dichloromethane. Cuprous iodide (10.45 mg, 0.055 mmol, 1 eq), diisopropylethylamine (7.1 mg, 0.055 mmol, 1 eq) and acetic acid (3.3 mg, 0.055 mmol, 1 eq) were added as catalysts, and the reaction was stirred at room temperature for 6 h in the dark. The mixture was centrifuged at a high speed (12000 rpm min^{-1}) to remove the precipitate. The organic phase was concentrated and separated by reverse column chromatography [C18, MeOH (0.1%TFA) / H_2O (0.1%TFA) = 50/100, v/v] to obtain the product CEAF-OMe (75 mg, yield: 48%). The product was characterized by Maldi-Tof/Tof-MS spectrum (Figure S59).

Synthesis of CEAF-NHS. *prop*-Lyso1005 (50 mg, 0.055 mmol, 1 eq) and N_3-PEG$_{2000}$-NHS (150 mg, 0.11 mmol, 1.2 eq) were dissolved in 10 mL of dichloromethane. Cuprous iodide (10.45 mg, 0.055 mmol,

1 eq), diisopropylethylamine (7.1 mg, 0.055 mmol, 1 eq) and acetic acid (3.3 mg, 0.055 mmol, 1 eq) were added as catalysts, and the reaction was stirred at room temperature for 6 h in the dark. The mixture was centrifuged at a high speed (12000 rpm min^{-1}) to remove the precipitate. The organic phase was concentrated and separated by reverse column chromatography [C18, MeOH (0.1%TFA) / H_2O (0.1%TFA) = 60/100, v/v] to obtain the product CEAF-NHS (60 mg, yield: 40%). The product was characterized by Maldi-Tof/Tof-MS spectrum (Figure S60).

Preparation of CEAF-OMe Nanoaggregates. CEAF-OMe (5 mg) was dissolved in 1 mL of dimethyl sulfoxide. The solution was dispersed evenly under ultrasonication and then filtered with an organic filter membrane of 0.22 microns. 9 mL of deionized water was prepared in a clean glass bottle. The above CEAF-OMe solution was quickly added under rapid magnetic stirring. The reaction was stirred for five more minutes. The solution was collected and dialyzed three times (2 h per time) with dialysis tube (Mw=3500 Da) to obtain the CEAF-OMe nanoaggregates (0.5 mg mL^{-1}).

Preparation of CEAF-NHS Nanoaggregates. CEAF-NHS nanoaggregates were prepared by the similar procedure as CEAF-OMe nanoaggregates (0.5 mg mL^{-1}).

Preparation of CEAF-RGD Nanoaggregates. To the above CEAF-NHS nanoaggregates solution (1 eq), adding cRGDfK peptide (5 eq). The reaction was stirred for 12 h in the dark at room temperature. The solution was collected and dialyzed three times (2 h per time) with dialysis tube (Mw=3500 Da) to obtain the CEAF-RGD nanoaggregates (0.5 mg mL^{-1}).

Preparation of Lyso1005 micelles/*boc*-Lyso1005 micelles. The nanomicelles were prepared using modified film hydration technique. In a typical procedure, Lyso1005/boc-Lyso1005 were firstly dissolved in DMSO to obtain stock solution with concentration of 5 mM. 100 μL (0.5 μmol) stock solution of Lyso1005/boc-Lyso1005 was mixed with 1.6 mL PEG-PCL (25 mg mL^{-1} in THF) at a mass ratio of ~1:100. The solvent was removed by vacuum rotary evaporation to form a dry dye-containing lipid film. The dried film was hydrated with 10 mL deionized water at 80 °C and sonication for 30 s to make the clear nanomicelles solution with dye concentration of 50 μM. The nanomicelle solution was

further concentrated if necessary by using a 30 K Amicon Ultra filter (Millipore Corporation) under centrifugation at 2000 g min-1 for 5 min.

Characterization

All ^{1}H-NMR and ^{13}C-NMR spectra were acquired on a Bruker AV-400 spectrometer. Chemicals shifts are referenced to the residue solvent peaks and given in ppm. MALDI-TOF MS analyses were performed in positive reflection mode on a 5800 proteomic analyzer (Applied Biosystems, Framingham, MA, USA) with a Nd: YAG laser. UV-Vis-NIR absorption spectra were recorded on Lamda750S PerkinElmer. NIR-II fluorescence spectra were recorded on Edinburgh Fluorescence Spectrometer FLS980 instrument or Horiba Fluorescence Spectrometer instrument (Fluorolog^{TM} HORIBA Scientific) with external 808 nm (MDL-III-2W) and 940 nm (MDL-H-5W) semiconductor lasers (Changchun New Industries Optoelectronics Tech. Co., Ltd.) as excitation sources. In vivo NIR-II images were taken using NIRvana CCD camera (Princeton Instruments Inc.). Dynamic light scattering measurements were carried out on a Malvern Zetasizer 3600 (Malvern Instruments). Transmission electron microscopy (TEM) tests were performed using a High Contrast Transmission Electron Microscope (HT7800). NIR-II intravital imaging was carried out with an epifluorescence microscopy system with 640×512 pixel 2D InGaAs NIRvana camera. Flow cytometry analyses were performed using Beckman Coulter Gallios flow cytometer.

General procedure for absorbance and fluorescence determination

A series of standard pH buffer solutions ($MeCN/H_2O$=1/1, v/v) were prepared by mixing 0.2 M Na_2HPO_4, 0.2 M KH_2PO_4 and 0.2 M H_3PO_4 at varied volume ratios, and the accurate pH values were measured by Delta 320 pH-meter. Then, 2 mL of the phosphate buffer and 5 μL of the stock solution of dyes (2 mM) in dimethyl sulfoxide solution were mixed. The resulting solution was mixed well, and an appropriate portion of the solution was transferred to a quartz cell of 1-cm optical length to measure absorbance against the corresponding reagent blank or fluorescence with λ_{ex} = 808 nm.

Determination of p*K*a values

The calculation of pKa values was performed with Origin 2017 software (OriginLab, Northhampton, MA), using the Bolzmman fitting function:

$$y = \frac{A_1 - A_2}{1 + e^{x - x_0 / dx}} + A_2$$

On the basis of the calculated equation of pKa, x_0 corresponds to the pKa value.

In vitro mimicking of aggregate/disaggregate property

100 μL CEAF-OMe nanoaggregates (0.5 mg mL^{-1}) were dissolved in 1.9 mL phosphate buffer (pH=7.0 or pH=5.0). 100 μL CEAF-OMe nanoaggregates (0.5 mg mL^{-1}) and 0.015% surfactants Triton X100, Tween-80 or SDS were dissolved in 1.9 mL phosphate buffer (pH=7.0 or pH=5.0). 100 μL CEAF-OMe nanoaggregates (0.5 mg mL-1) and 0.15% DSPE-PEG2000 were dissolved in 1.9 mL phosphate buffer (pH=7.0 or pH=5.0). The above samples were incubated under ultrasound for 5 min. The prepared samples were transferred to a quartz cell of 1-cm optical length to measure fluorescence with λ_{ex} = 808 nm.

Measurement of the stability of CEAF-OMe nanoaggregates

PBS, PBS with 0.015% Triton X100, simulated tissue fluid, FBS and blood of both pH=7.0 and pH=5.0 were prepared. 10 μL CEAF-OMe nanoaggregates (0.5 mg mL^{-1}) was dissolved in the above solvents of 500 μL, respectively. The prepared samples were observed under 940 nm laser radiation ((laser output power density = 0.23 W cm^{-2}, working distance = 30 cm), 1000 nm, 1100nm and 1200nm long pass filters were used. Images were processed with the LightField imaging software, ImageJ and MATLAB.

Determination of Critical Aggregation Concentration values

All required solvents (phosphate buffer (PBS, pH=7.0), fetal bovine serum (FBS), phosphate buffer with 0.015% Triton X100 (pH=7.0 or pH=5.0), and phosphate buffer with 0.3% Triton X100 (pH=7.0 or pH=5.0)) were prepared in advance. A certain amount of CEAF-OMe solids were dissolved in dimethyl sulfoxide to gain a stock solution of 1.6 μM. Then a series of solutions of different concentrations in different solvents were prepared and were dispersed evenly under ultrasound for 5 min. The prepared

samples were transferred to a quartz cell of 1-cm optical length to measure fluorescence with λ_{ex} = 940 nm. The emission spectrums in the 960-1400 nm region were acquired. Then the concentration-dependent changes of fluorescence intensities of CEAF-OMe in every solvent was obtained and the calculation of CAC values (Critical Aggregation Concentration) was performed with Origin 2017 software (OriginLab, Northhampton, MA), respectively.

Measurement of fluorescence quantum yield (QY)

Quantum yields (Φ_{fl}) were determined in various solvents relative to IR26 (Φ_{fl} = 0.05% in DCE),[3] from plots of integrated fluorescence intensity vs. absorbance, according to the following relationship:

$$\Phi_{fl.s} = \Phi_{fl.r} \times \frac{n_s^2}{n_r^2} \times \frac{K_s}{K_r}$$

where subscripts r and s denote standard and test sample, respectively, Φ_{fl} is the fluorescence quantum yield, K is the slop of the integrated fluorescence intensity vs. absorbance plot, and n is the refractive index of the solvent. Measurements were performed with the absorbance at 808 nm of all dye solutions < 0.1 in order to maximize illumination homogeneity and optical transparency. The 808 nm laser was used as the excitation source and the emission spectrum in the 850-1500 nm region was acquired in fluorescence spectrometer.

Quantum Calculations

All the quantum chemical calculations were done with the Gaussian 09 suite.[4] The geometry optimizations of the fluorophores were performed using density functional theory (DFT) with Becke's three-parameter hybrid exchange function with Lee−Yang−Parr gradient-corrected correlation functional (B3-LYP functional) and 6-31G(d) basis set. No constraints to bonds/angles/dihedral angles were applied in the calculations, and all atoms were free to optimize. The electronic transition energies and corresponding oscillator strengths were calculated with time-dependent density functional theory (TDDFT) at the B3LYP/6-311G (d, p) level.

Measurement of photo-stability

Lyso880, Lyso1005, Lyso855 and Lyso950 were dissolved in DMSO to obtain stock solutions of 5 mM. ICG was dissolved in deionized water to obtain the stock solution of 5 mM. A certain amount of the above stock solutions were dissolved in the mixed solvent of acetonitrile and water (1:1, v/v) of both pH=7.0 and pH=5.0, respectively, to fixed their absorption at 808 nm to a same absorbance value (0.27). All prepared samples were transferred to a quartz cell of 1-cm optical length to measure fluorescence with λ_{ex} = 808 nm for 1000 s.

Measurement of chemical stability

Preparation of various bioactive reagents solutions:

(1) H_2O_2: Commercial H_2O_2 solution (30 % in H_2O) was used directly. The concentration of the H_2O_2 stock solution was determined by measuring the absorbance at 240 nm with a molar extinction coefficient of 43.6 $M^{-1}cm^{-1}$;

(2) ClO^-: Hypochlorite stock (ClO^-, 50 mM in H_2O, pH = 12) was prepared by dilution of commercial NaClO solution in water (pH = 12) and assayed using a spectrophotometer using ε 293nm = 350 $cm^{-1}M^{-1}$;

(3) GSH: A 20 mM stock solution of GSH was prepared in MeCN/PBS (V/V = 1:1, pH=5.0 or pH=7.0);

(4) $ONOO^-$: Peroxynitrite stock (30 mM in 0.1 M NaOH) was prepared following literature procedure [5] and assayed using a spectrophotometer using ε 302nm = 1670 $cm^{-1}M^{-1}$.

Measurement of chemical stability:

2 μL stock solutions (5 mM) of Lyso880, Lyso1005, Lyso855 or Lyso950 were added into the mixed solvent of acetonitrile and water (1:1, v/v, pH=7.0 or pH=5.0), respectively. Absorption spectra were recorded at 5 min after mixing with different amount of bioactive reagents solutions.

Optical setup for wield-field whole-body NIR-II imaging

NIR-II fluorescence images were acquired using a home-built imaging setup, in which the excitation light was provided by a 940 nm laser coupled to a 450-μm core metal-cladded multimode fiber (MDL-H-5W). The emitted light was directed from the imaging stage to the camera and passed through different filter sets (Thorlabs and Edmund Optics) as required by the experiments to ensure the images taken in

different sub-regions, and focused onto the thermo-electric cooling two-dimensional InGaAs camera (NIRvana: 640, 640 × 512 pixel; Princeton Instruments, response 900-1700 nm). The whole assembly was surrounded by a partial enclosure to eliminate excess light while enabling manipulation of the field of view during operation. Different exposure times were used to achieve sufficient signal intensities. All images were background and blemish corrected within the LightField imaging software, followed by processing with MATLAB or ImageJ software.

Optical setup for NIR-II epifluorescence microscopy

In vivo NIR-II epifluorescence microscopy. NIR-II intravital microscopic imaging was performed on a commercial Nikon LV-TV microscope equipped with 730 nm (MDL-XD-730-5W) or 940 nm (MDL-H-5W) lasers, an objective with high light transmission over the near-infrared spectral range (XLPLN25XWMP2, 25X, NA=1.05, Olympus), and imaged onto a thermo-electric cooling two-dimensional InGaAs camera (NIRvana: 640, 640 × 512 pixel; Princeton Instruments, response 900-1700 nm). 830 nm (Di02-R830-25x36, Sermrock) or 1000 nm (DMLP1000R, Thorlab) long-pass dichroic mirrors was used for imaging. A 1100 nm long-pass filter (FELH1100, Thorlab), and an exposure time of 100 ms were used to image the fluorescence signal of CEAF probes. A 850 nm long-pass filter (FELH850, Thorlab) and an exposure time of 100 ms was used to image the fluorescence signal of ICG.

NIR-II epifluorescence microscopy for subcellular organelles. NIR-II microscopic imaging for subcellular organelles was performed on a commercial OLYMPUS IX71 microscope equipped with 808 nm (MDL-XF-808-10 W) or 655 nm (MDL-XD-655-5 W) lasers and an UPlanFL N 40x (or UPlanFL N 60x) objective, and imaged onto a thermo-electric cooling two-dimensional InGaAs camera (NIRvana: 640, 640 × 512 pixel; Princeton Instruments, response 900-1700 nm). 775 nm (Di02-R775-25x36, Sermrock) or 1000 nm (DMLP1000R, Thorlab) long-pass dichroic mirrors was used for imaging. A 1000 nm long-pass filter (FELH1000, Thorlab), and an exposure time of 500~5000 ms were used to image the fluorescence signal of CEAF probes. A 850 nm long-pass filter (FELH850, Thorlab) and an exposure time of 500~5000 ms was used to image the fluorescence signal of the Lysosomal tracker.

Cell viability

Human lung cells (A549) were provided by Stem Cell Bank, Chinese Academy of Science. Cells were excluded mycoplasma contamination by mycoplasma Detection Kit. All cells were cultured in Dulbecco's Modified Eagle medium (DMEM) supplemented with 10% FBS and 1% Penicillin-Streptomycin at 37 °C in a humidified atmosphere of 5% CO_2. A549 cells were cultured in a 96-well plate (8×103 cells/well) after 24 h incubation, the medium was replaced with 100 μL of fresh DMEM containing Lyso880, Lyso1005, Lyso855, Lyso950 with concentrations of 0, 0.1, 0.25, 0.5, 1.0, 2.5 and 5.0 μM, respectively. Cells were incubated further for 24 h. To detect the cytotoxicity, 10 μL of Cell Counting Kit-8 (CCK-8) solution was added to each well of the microliter plate and the plate was incubated in the CO_2 incubator for additional 4 h. Enzyme dehydrogenase in living cells was oxidized by this kit to orange carapace. The quality was assessed calorimetrically by using a multi-reader (TECAN, Infinite M200, Germany). The measurements were based on the absorbance values at 450 nm. Following formula was used to calculate the viability of cell growth:

Cell Viability (%) = (mean absorbance value of treatment group/mean absorbance value of control group) × 100%.

Cell imaging

Cell culture. A549 cells and 4T1 cells were grown on glass-bottom culture dishes (MatTek Co.) in 1640 medium supplemented with 10% (v/v) FBS, 100 U mL^{-1} penicillin and 100 μg mL^{-1} streptomycin in a humidified 37 ℃, 5% CO_2 incubator. Before use, the cells were washed with fresh 1640 medium.

Co-localization fluorescence imaging of Lyso dyes. In this experiment, cells (A549 or 4T1) were respectively incubated with dyes (Lyso880, Lyso1005, Lyso855 or Lyso950, 10 uM) for 12 h, washed three times with PBS (pH 7.4), and then incubated with Lyso-Tracker (LysoBrite™ NIR, 50 nM) for 30 min. Before fluorescence imaging, the adherent cells were further washed for three times with PBS (pH 7.4) to remove the excess LysoBrite™ NIR and then fixed with 4% paraformaldehyde solution. Fluorescence imaging experiments were performed on an Olympus-IX71 epifluorescence microscope with excitations at either 655 nm (for LysoBrite™ NIR) or 808 nm (for our dyes) through an UPlanFL

N 40x (or UPlanFL N 60x) objective. 750-1000 nm (for LysoBrite™ NIR) and 850-1700 nm (for our dyes) corresponding fluorescence emissions were collected, respectively.

Co-localization fluorescence imaging of CEAF-OMe. In this experiment, 4T1 cells were firstly incubated with CEAF-OMe (10 μM) for 12 h, washed three times with PBS (pH 7.4), and then incubated with Lyso-Tracker (LysoBrite™ NIR, 50 nM) for 30 min. Before fluorescence imaging, the adherent cells were further washed for three times with PBS (pH 7.4) to remove the excess LysoBrite™ NIR and then fixed with 4% paraformaldehyde solution. Fluorescence imaging experiments were performed on an OLYMPUS IX71 epifluorescence microscope with excitations at either 655 nm (for LysoBrite™ NIR, MDL-XD-655-5 W) or 808 nm (for CEAF-OMe, MDL-XF-808-10 W) through an UPlanFL N 40x (or UPlanFL N 60x) objective. 750-1000 nm (for LysoBrite™ NIR) and 850-1700 nm (for CEAF-OMe) corresponding fluorescence emissions were collected, respectively.

Disaggregation and fluorescence activation with A549 and 4T1 cells. In this experiment, CEAF-OMe nanoaggregates were incubated with A549 cells (or 4T1 cells, 10 μM) for 12 h. Then epifluorescence microscopy experiments were performed on the Nikon LV-TV epifluorescence microscope with excitation at 940 nm through an objective (XLPLN25XWMP2, 25X, NA=1.05, Olympus). 1200-1700 nm corresponding fluorescence emission was collected.

Disaggregation and fluorescence activation with RAW264.7. In this experiment, CEAF-RGD nanoaggregates (10 μM) were incubated with RAW264.7 (with LPS, 100 ng mL^{-1}) and RAW264.7 (without LPS) for 2 h (LPS serves as an inducer to polarize the RAW264.7 cells toward the M1 phenotype). Meanwhile, CEAF-NHS nanoaggregates (10 μM) blockig with glycine were incubated with RAW264.7 (with LPS, 100 ng mL^{-1}) for 2 h. Then epifluorescence microscopy experiments were performed on the Nikon LV-TV epifluorescence microscope with excitations at 940 nm through an objective (XLPLN25XWMP2, 25X, NA=1.05, Olympus). 1200-1700 nm corresponding fluorescence emission was collected.

Animal experiments

Animal handling

All animal procedures were performed in accordance with the guidelines of the Institutional Animal Care and Use Committee of Fudan University, in agreement with the institutional guidelines for animal handling. All of the animal experiments were authorized by the Shanghai Science and Technology Committee. 13-15 g female BALB/c nude mice were purchased from Shanghai Jiesijie Laboratory Animal Co., LTD and randomly selected from cages for all imaging experiments. No blinding was performed. Before imaging, all mice were anaesthetized using rodent ventilator with 2 L min^{-1} air mixed with 4% isoflurane. During the time course of imaging the mouse was kept anaesthetized by a nose cone delivering 2 L min^{-1} air mixed with 4% isoflurane.

Tumor model. 1×10^7 CT26 cells suspended in 50 μL FBS-free DMEM were subcutaneously injected into the nude mice. When the tumor volume reached ~200 mm^3, the tumor mass was cut into small pieces, which were then implanted into other mice subcutaneously. In vivo imaging and biodistribution studies were performed when tumor volume reached ~150 mm^3.

Traumatic ankle injury model. 13-15 g male BALB/c nude mice were deep anesthetic and positioned in a cradle to position and fix one hind limb. Then the tibia and talus connected by the ankle joint were dislocated to induce ankle injury. Swelling and abnormal movement of the injured ankle were considered as model success.

In vivo bioimaging for subcutaneous tumor. NIR-II imaging signals at different time points were collected by InGaAs CCD with 940 nm excitation (laser output power density = 0.23 W cm^{-2}, working distance = 30 cm), 1000 nm, 1100nm and 1200nm long pass filters were used. Images were processed with the LightField imaging software, ImageJ and MATLAB.

Surgical resection surgery of CT26 tumors. The CT26 tumors were removed under InGaAs CCD with 940 nm laser irradiation (laser output power density = 0.16 W cm^{-2}, working distance = 30 cm) 3 h after injection of CEAF-OMe nanoaggregates (50 μM, 200 μL) and Lyso1005/PEG-PCL micelles (50 μM, 200 μL), respectively. Then the eyevisible suspicious tumor was removed under fluorescence guidance (**Supplementary Video 1, 2**). The camera was set to continuously expose using LightField imaging

software. The exposure time for all images shown in the movies was 500 ms. Images and videos were processed with MATLAB software. All collected tissues were further analyzed by H & E staining.

Intravital Microscopy of tumor-bearing mouse. In a CT26 tumor-bearing mouse, CEAF-OMe nanoaggregates (50 μM, 200 μL) was intravenously injected. After 3 h, the tumor was lighted up, and the mouse was immobilized under the intravital epifluorescence microscopy system. ICG (a FDA-approved dye, 50 μM, 50 μL) was intravenously injected as an vascular contrast agent for two-color epifluorescence microsopy. Then double-channel fluorescence signals were recorded under 940 nm (for CEAF-OMe nanoaggregates) or 730 nm (for ICG) excitation respectively and a merged picture was obtained with the LightField imaging software, ImageJ and MATLAB.

Flow cytometry

The mice were euthanized a day after ankle injury, and healthy mice were set as control. Then spleens and ankles of mice were separated off. Leukocytes in spleen were isolated by Mouse 1X Lymphocyte Seperation Medium (Dakewe, 7211011). The cell isolation procedures of ankles were performed according to the protocol as previous reported.[6] Then, the cells were stained with CD45, CD11b, F4/80 and CD86 antibodies (1:200, diluted with 1% FBS/PBS) for 30 min at room temperature. After staining, all cells were subjected to 300 mesh cell screens before analyzing in a Beckman Coulter Gallios flow cytometer.

HE staining

The CT26 tumor-bearing mice were euthanized after surgical resection surgery. And the traumatic ankle injury mice were dissected to isolate the ankles and spleens, which were then fixed with 4% paraformaldehyde for 24 h. Next, all samples were dehydrated with ethanol and embedded in paraffin before 6 μm sectioning. Then, sections of tissues were stained with hematoxylin and eosin.

Part B: Figure Ss

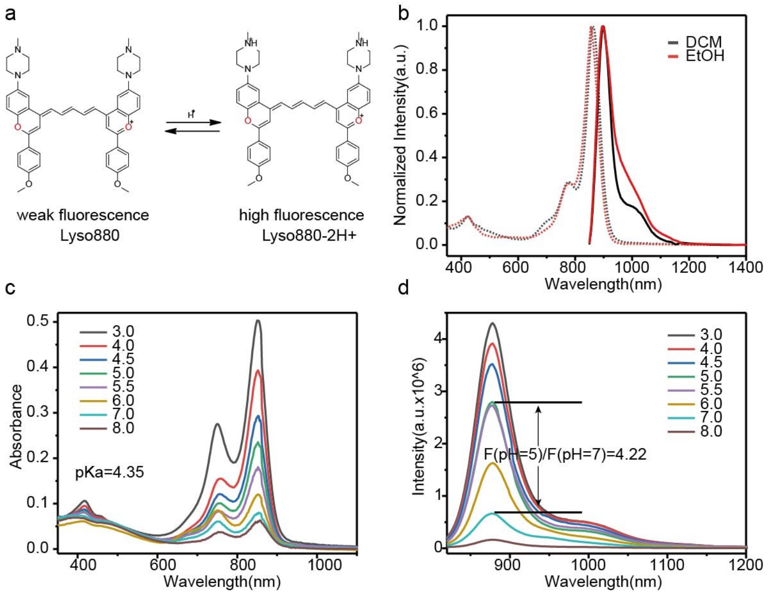

Figure S1. Fluorescence and acidic sensitivity of Lyso880. (a) The general formula for protonation of Lyso880. (b) The absorption (dash line) and emission (solid line) spectra in different organic solvents (dichloromethane and ethyl alcohol). (c) Absorption spectra of Lyso880 in a series of standard pH buffer solutions (MeCN/PBS=1/1, v/v, pH=3.0 ~ 8.0). The bimodal absorption is due to the aggregation of dyes in aqueous solution. (d) Emission spectra of Lyso880 in a series of standard pH buffer solutions (MeCN/PBS=1/1, v/v, pH=3.0 ~ 8.0). The fluorescence enhancement factor from pH=7.0 to pH=5.0 was calculated.

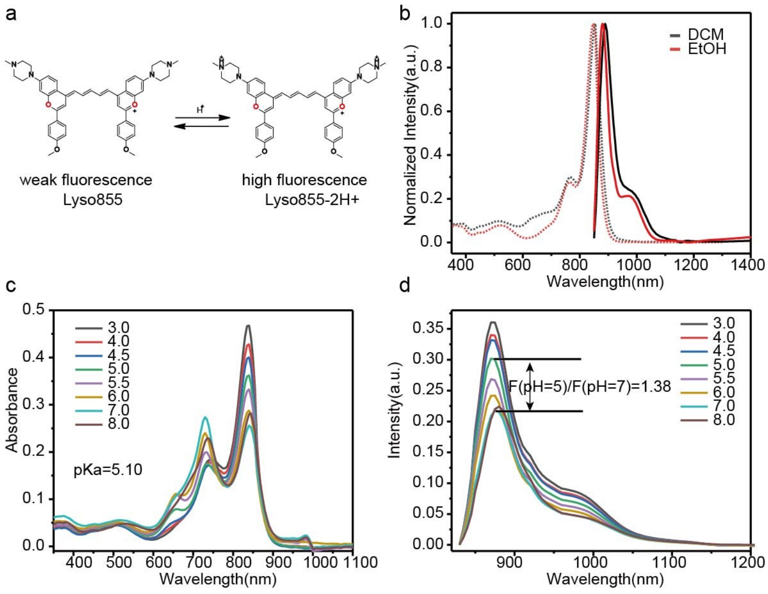

Figure S2. Fluorescence and acidic sensitivity of Lyso855. (a) The general formula for protonation of

Lyso855. (b) The absorption (dash line) and emission (solid line) spectra in different organic solvents (dichloromethane and ethyl alcohol). (c) Absorption spectra of Lyso855 in a series of standard pH buffer solutions (MeCN/PBS=1/1, v/v, pH=3.0 ~ 8.0). The bimodal absorption is due to the aggregation of dyes in aqueous solution. (d) Emission spectra of Lyso855 in a series of standard pH buffer solutions (MeCN/PBS=1/1, v/v, pH=3.0 ~ 8.0). The fluorescence enhancement factor from pH=7.0 to pH=5.0 was calculated.

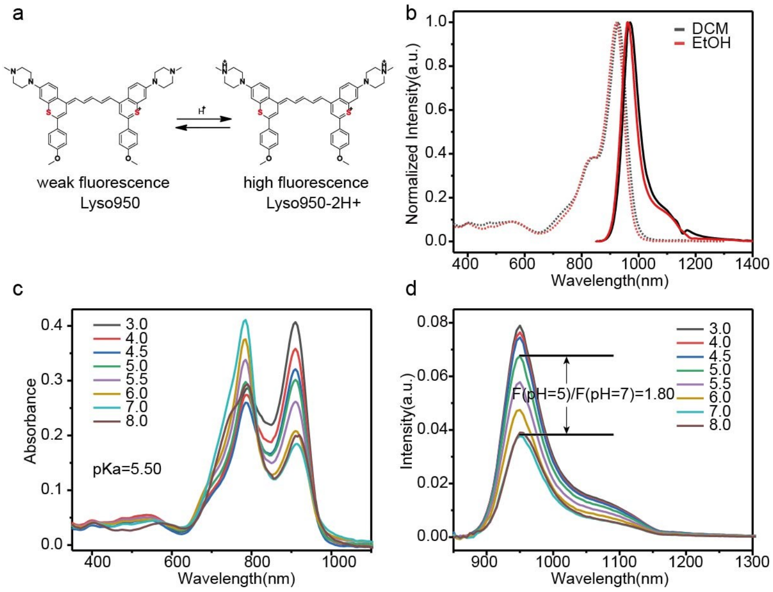

Figure S3. Fluorescence and acidic sensitivity of Lyso950. (a) The general formula for protonation of Lyso950. (b) The absorption (dash line) and emission (solid line) spectra in different organic solvents (dichloromethane and ethyl alcohol). (c) Absorption spectra of Lyso950 in a series of standard pH buffer solutions (MeCN/PBS=1/1, v/v, pH=3.0 ~ 8.0). The bimodal absorption is due to the aggregation of dyes in aqueous solution. (d) Emission spectra of Lyso950 in a series of standard pH buffer solutions (MeCN/PBS=1/1, v/v, pH=3.0 ~ 8.0). The fluorescence enhancement factor from pH=7.0 to pH=5.0 was calculated.

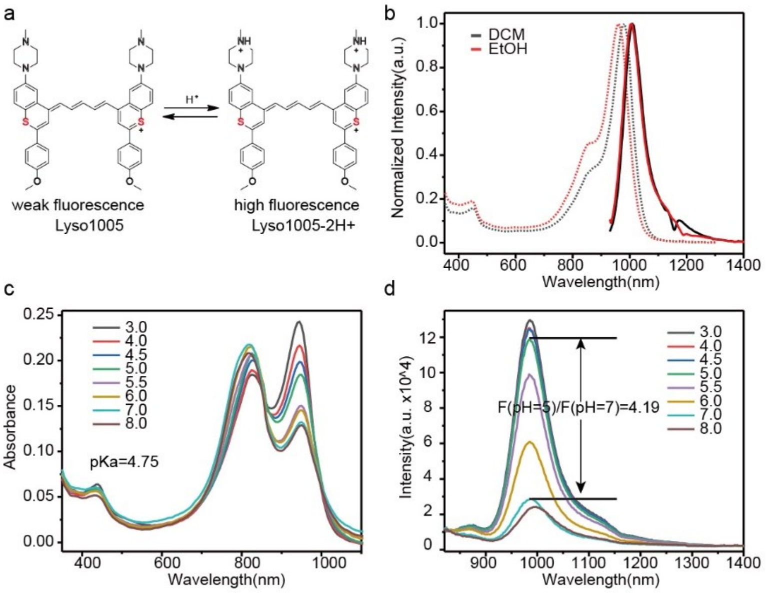

Figure S4. Fluorescence and acidic sensitivity of Lyso1005. (a) The general formula for protonation of Lyso1005. (b) The absorption (dash line) and emission (solid line) spectra in different organic solvents (dichloromethane and ethyl alcohol). (c) Absorption spectra of Lyso1005 in a series of standard pH buffer solutions (MeCN/PBS=1/1, v/v, pH=3.0 ~ 8.0). The bimodal absorption is due to the aggregation of dyes in aqueous solution. (d) Emission spectra of Lyso1005 in a series of standard pH buffer solutions (MeCN/PBS=1/1, v/v, pH=3.0 ~ 8.0). The fluorescence enhancement factor from pH=7.0 to pH=5.0 was calculated.

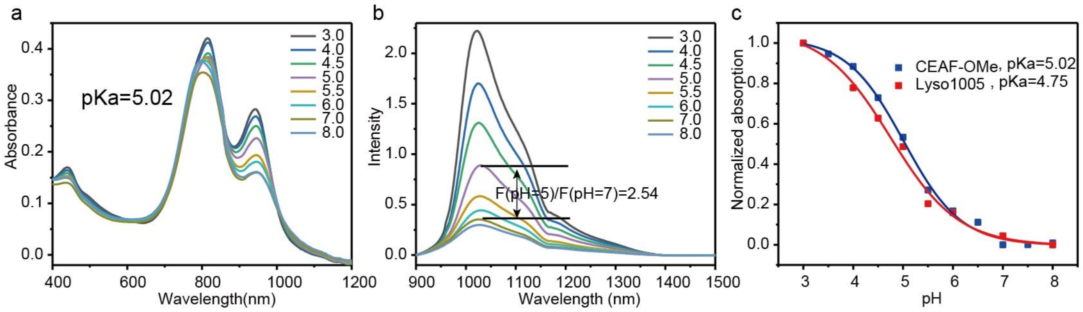

Figure S5. Fluorescence and acidic sensitivity of CEAF-OMe. (a) Absorption spectra of CEAF-OMe in a series of standard pH buffer solutions (MeCN/PBS=1/1, v/v, pH=3.0 ~ 8.0). The bimodal absorption is due to the aggregation of dyes in aqueous solution. (b) Emission spectra of CEAF-OMe in a series of standard pH buffer solutions (MeCN/PBS=1/1, v/v, pH=3.0 ~ 8.0). The fluorescence enhancement factor from pH=7.0 to pH=5.0 was calculated. (c) Plot of absorbance of Lyso1005 (red) and CEAF-OMe (blue) at 960 nm versus pH values. Curve fitting was based on a Boltzmann function in origin software.

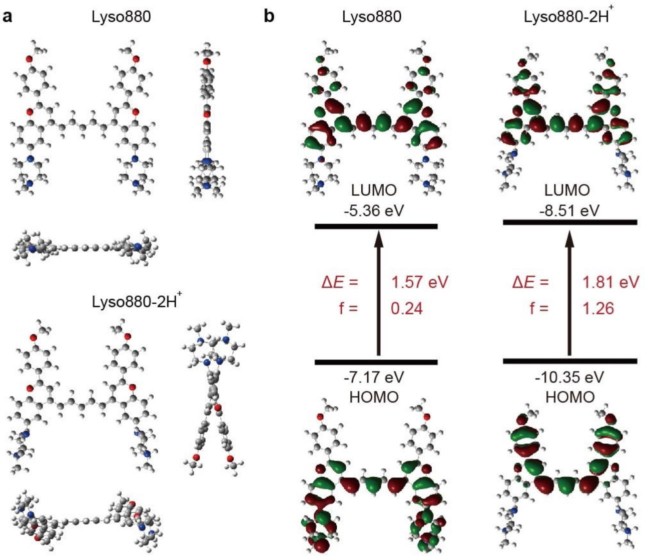

Figure S6. DFT calculations of Lyso880. (a) Optimized molecule geometries of Lyso880 and Lyso880-2H$^+$ (protonated) at different views. (b) Comparison of the HOMO and LUMO energy levels, S_0–S_1 excitation energies for Lyso880 and Lyso880-2H$^+$, where Δ E represents the energy difference between HOMO and LUMO, indicating the absorption wavelength changes before and after protonation, f is related to the molar absorption coefficient.

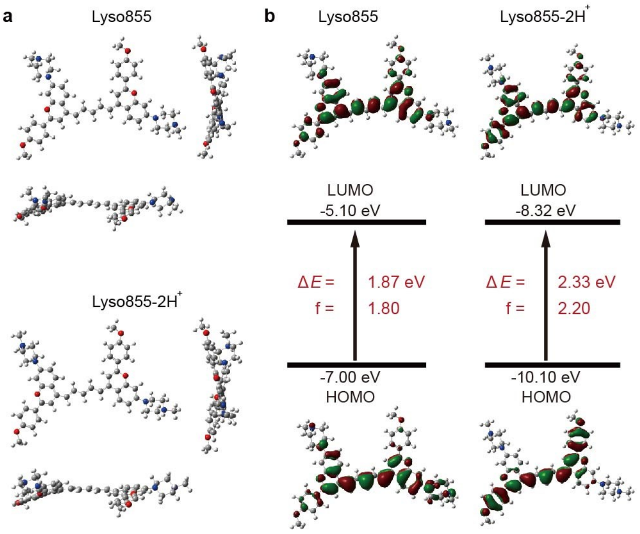

Figure S7. DFT calculations of Lyso855. (a) Optimized molecule geometries of Lyso855 and Lyso855-2H$^+$ (protonated) at different views. (b) Comparison of the HOMO and LUMO energy levels, S_0–S_1 excitation energies for Lyso855 and Lyso855-2H$^+$, where Δ E represents the energy difference between HOMO and LUMO, indicating the absorption wavelength changes before and after protonation, f is related to the molar absorption coefficient.

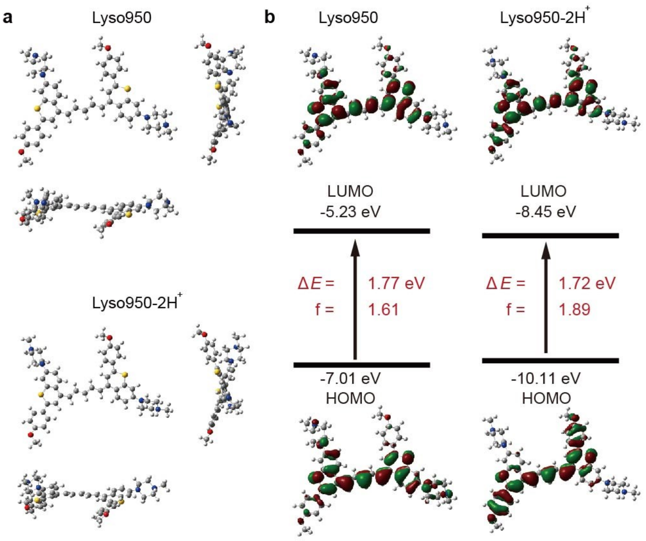

Figure S8. DFT calculations of Lyso950. (a) Optimized molecule geometries of Lyso950 and Lyso950-2H$^+$ (protonated) at different views. (b) Comparison of the HOMO and LUMO energy levels, S_0–S_1 excitation energies for Lyso950 and Lyso950-2H$^+$, where Δ E represents the energy difference between HOMO and LUMO, indicating the absorption wavelength changes before and after protonation, f is related to the molar absorption coefficient.

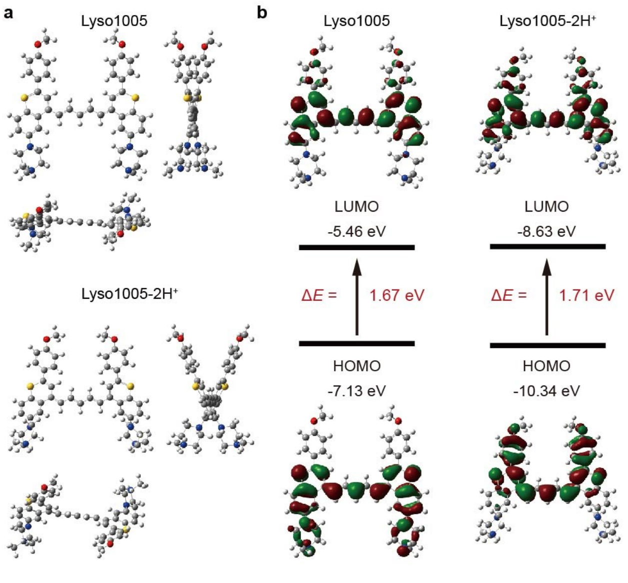

Figure S9. DFT calculations of Lyso1005. (a) Optimized molecule geometries of Lyso1005 and Lyso1005-2H$^+$ (protonated) at different views. (b) Comparison of the HOMO and LUMO energy levels, S_0–S_1 excitation energies for Lyso1005 and Lyso1005-2H$^+$, where Δ E represents the energy difference between HOMO and LUMO, indicating the absorption wavelength changes before and after protonation, f is related to the molar absorption coefficient.

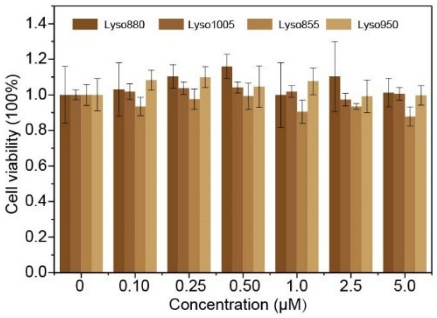

Figure S10. Cell viability measurement of dyes. 24 h incubation of A549 cells with synthesized Lyso880, Lyso1005, Lyso855 and Lyso950 under different concentrations, respectively. Values are the mean ± s.d. (n = 6).

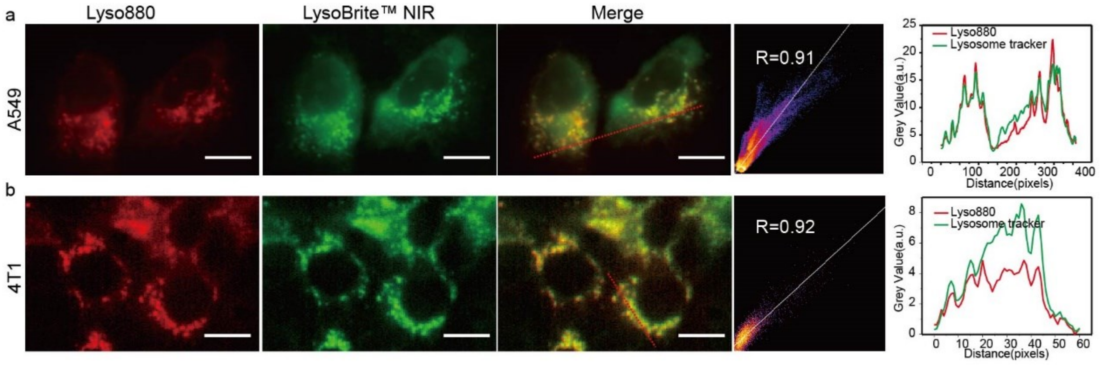

Figure S11. Lysosome-targeting properties of Lyso880 in A549 and 4T1 cells. (a) (1-3) Colocalization images of A549 cells stained with Lyso880 (red channel, $\lambda_{ex}/\lambda_{em}$ = 808/850-1700 nm) and LysoBrite™ NIR (green channel, $\lambda_{ex}/\lambda_{em}$ = 655/750-1000 nm); (4) The correlation of Lyso880 and LysoBrite™ NIR intensities. (5) Intensity profiles within the ROI (red dash line in (3)) of Lyso880 and LysoBrite™ NIR across A549 cells. (b) (1-3) Colocalization images of 4T1 cells stained with Lyso880 (red channel, $\lambda_{ex}/\lambda_{em}$ = 808/850-1700 nm) and LysoBrite™ NIR (green channel, $\lambda_{ex}/\lambda_{em}$ = 655/750-1000 nm); (4) The correlation of Lyso880 and LysoBrite™ NIR intensities. (5) Intensity profiles within the ROI (red dash line in (3)) of Lyso880 and LysoBrite™ NIR across 4T1 cells. Scale bar: 10 µm. Concentration: Lyso dye (10 µM), LysoBrite™ NIR (50 nM).

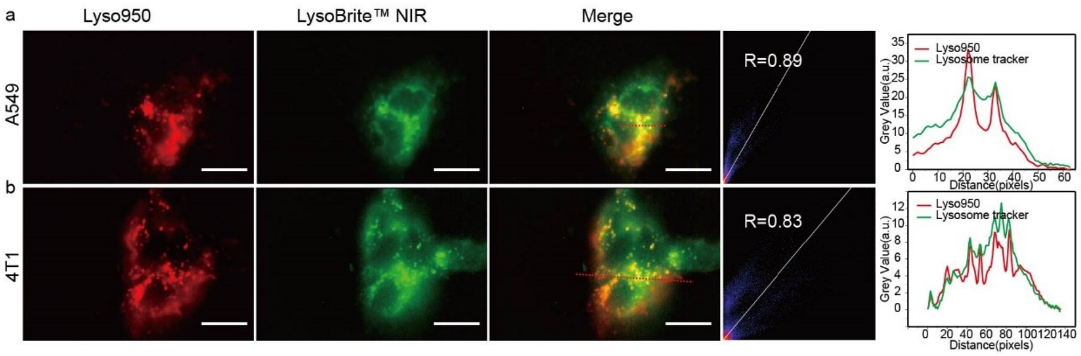

Figure S12. Lysosome-targeting properties of Lyso950 in A549 and 4T1 cells. (a) (1-3) Colocalization images of A549 cells stained with Lyso950 (red channel, $\lambda_{ex}/\lambda_{em}$ = 808/850-1700 nm) and LysoBrite™ NIR (green channel, $\lambda_{ex}/\lambda_{em}$ = 655/750-1000 nm); (4) The correlation of Lyso950 and LysoBrite™ NIR intensities. (5) Intensity profiles within the ROI (red dash line in (3)) of Lyso950 and LysoBrite™ NIR across A549 cells. (b) (1-3) Colocalization images of 4T1 cells stained with Lyso950 (red channel, $\lambda_{ex}/\lambda_{em}$ = 808/850-1700 nm) and LysoBrite™ NIR (green channel, $\lambda_{ex}/\lambda_{em}$ = 655/750-1000 nm); (4) The correlation of Lyso950 and LysoBrite™ NIR intensities. (5) Intensity profiles within the ROI (red dash line in (3)) of Lyso950 and LysoBrite™ NIR across 4T1 cells. Scale bar: 10 μm. Concentration: Lyso dye (10 μM), LysoBrite™ NIR (50 nM).

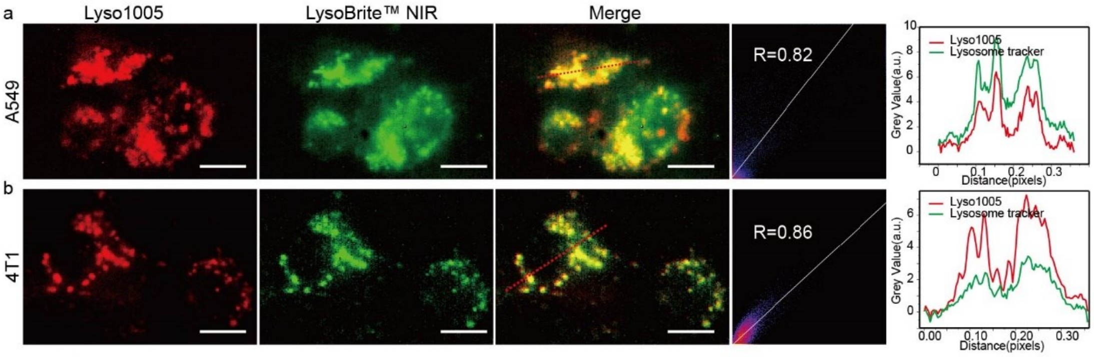

Figure S13. Lysosome-targeting properties of Lyso1005 in A549 and 4T1 cells. (a) (1-3) Colocalization images of A549 cells stained with Lyso1005 (red channel, $\lambda_{ex}/\lambda_{em}$ = 808/850-1700 nm) and LysoBrite™ NIR (green channel, $\lambda_{ex}/\lambda_{em}$ = 655/750-1000 nm); (4) The correlation of Lyso1005 and LysoBrite™ NIR intensities. (5) Intensity profiles within the ROI (red dash line in (3)) of Lyso1005 and LysoBrite™ NIR across A549 cells. (b) (1-3) Colocalization images of 4T1 cells stained with Lyso1005 (red channel, $\lambda_{ex}/\lambda_{em}$ = 808/850-1700 nm) and LysoBrite™ NIR (green channel, $\lambda_{ex}/\lambda_{em}$ = 655/750-1000 nm); (4) The correlation of Lyso1005 and LysoBrite™ NIR intensities. (5) Intensity profiles within the ROI (red dash line in (3)) of Lyso1005 and LysoBrite™ NIR across 4T1 cells. Scale bar: 10 μm. Concentration: Lyso

dye (10 μM), LysoBrite™ NIR (50 nM).

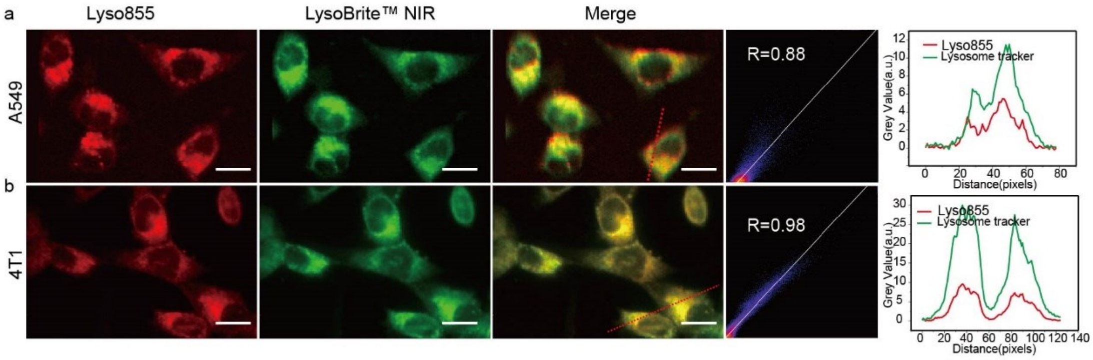

Figure S14. Lysosome-targeting properties of Lyso855 in A549 and 4T1 cells. (a) (1-3) Colocalization images of A549 cells stained with Lyso855 (red channel, $\lambda_{ex}/\lambda_{em}$ = 808/850-1700 nm) and LysoBrite™ NIR (green channel, $\lambda_{ex}/\lambda_{em}$ = 655/750-1000 nm); (4) The correlation of Lyso855 and LysoBrite™ NIR intensities. (5) Intensity profiles within the ROI (red dash line in (3)) of Lyso855 and LysoBrite™ NIR across A549 cells. (b) (1-3) Colocalization images of 4T1 cells stained with Lyso855 (red channel, $\lambda_{ex}/\lambda_{em}$ = 808/850-1700 nm) and LysoBrite™ NIR (green channel, $\lambda_{ex}/\lambda_{em}$ = 655/750-1000 nm); (4) The correlation of Lyso855 and LysoBrite™ NIR intensities. (5) Intensity profiles within the ROI (red dash line in (3)) of Lyso855 and LysoBrite™ NIR across 4T1 cells. Scale bar: 20μm. Concentration: Lyso dye (10 μM), LysoBrite™ NIR (50 nM).

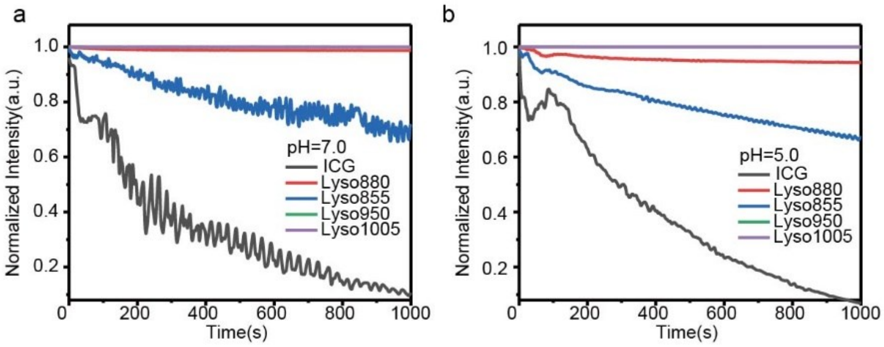

Figure S15. Photo-stability of Lyso dyes with the same absorbance value (0.27) at 808 nm. Normalized fluorescence intensity of dyes solutions in the mixed solvent of acetonitrile and water (1:1, v/v, pH=7.0 or pH=5.0) illuminated at 808 nm with corresponding laser at a power density of 0.5 W cm^{-2}. All Lyso dyes show great photo-stability as compared with ICG under 808 nm laser radiation for 1000 s. In addition, we find that substituting oxygen by sulfur tends to increase the photo-stability of Lyso dyes (Lyso855 vs Lyso950, Lyso880 vs Lyso1005).

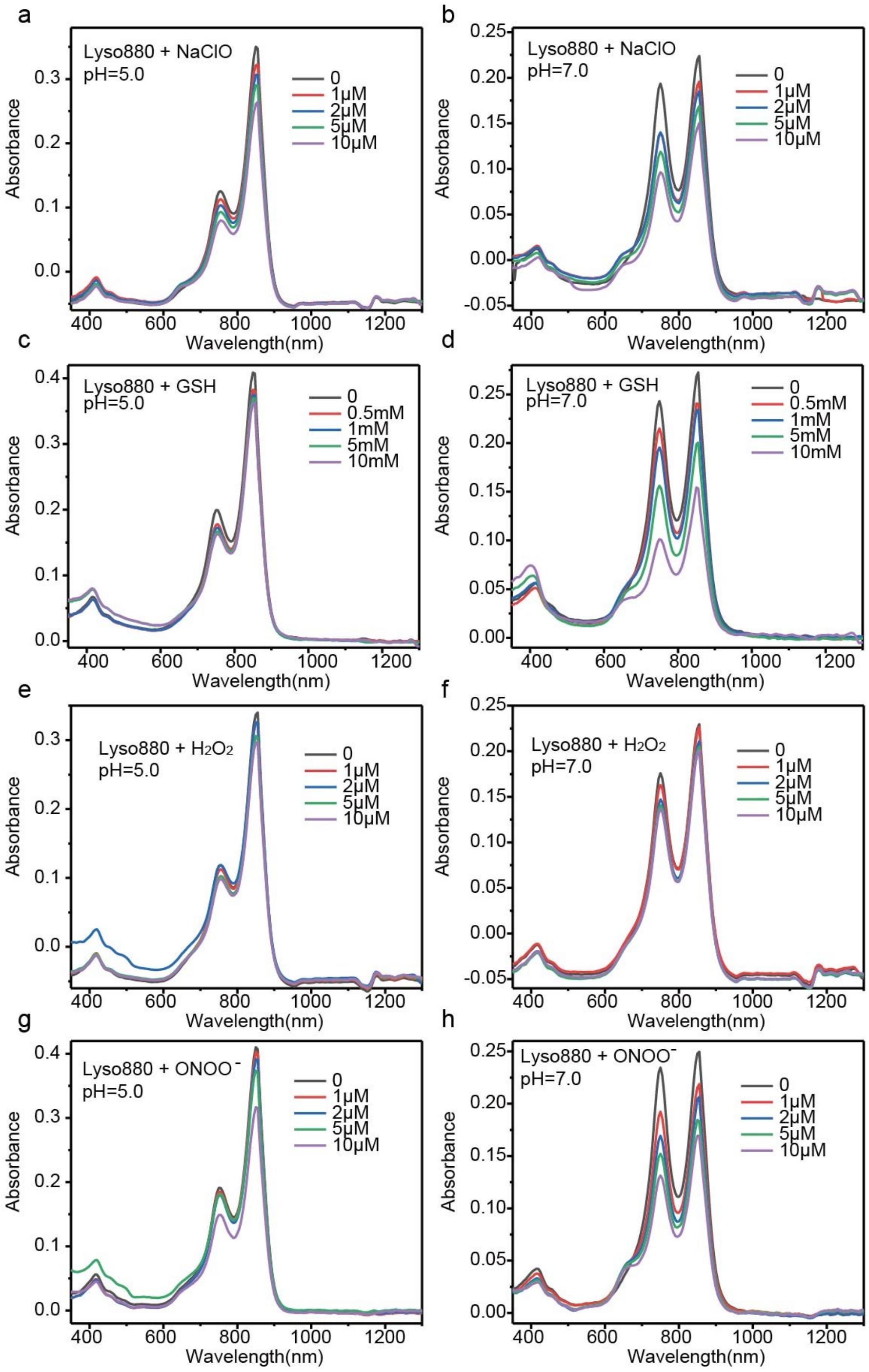

Figure S16. Chemical stability of Lyso880. Absorption spectra of Lyso880 against NaClO (a, b), GSH (c, d), H_2O_2 (e, f) and ONOO- (g, h) in the mixed solvent of acetonitrile and water (1:1, v/v, pH=7.0 or pH=5.0), spectra were recorded at 5 min after mixing with different amount of bioactive reagents solutions, the concentration of Lyso880 was 5 μM. The increase in the shoulder peak ranging from 650 ~ 800 nm is due to the aggregation of dyes in aqueous solution.

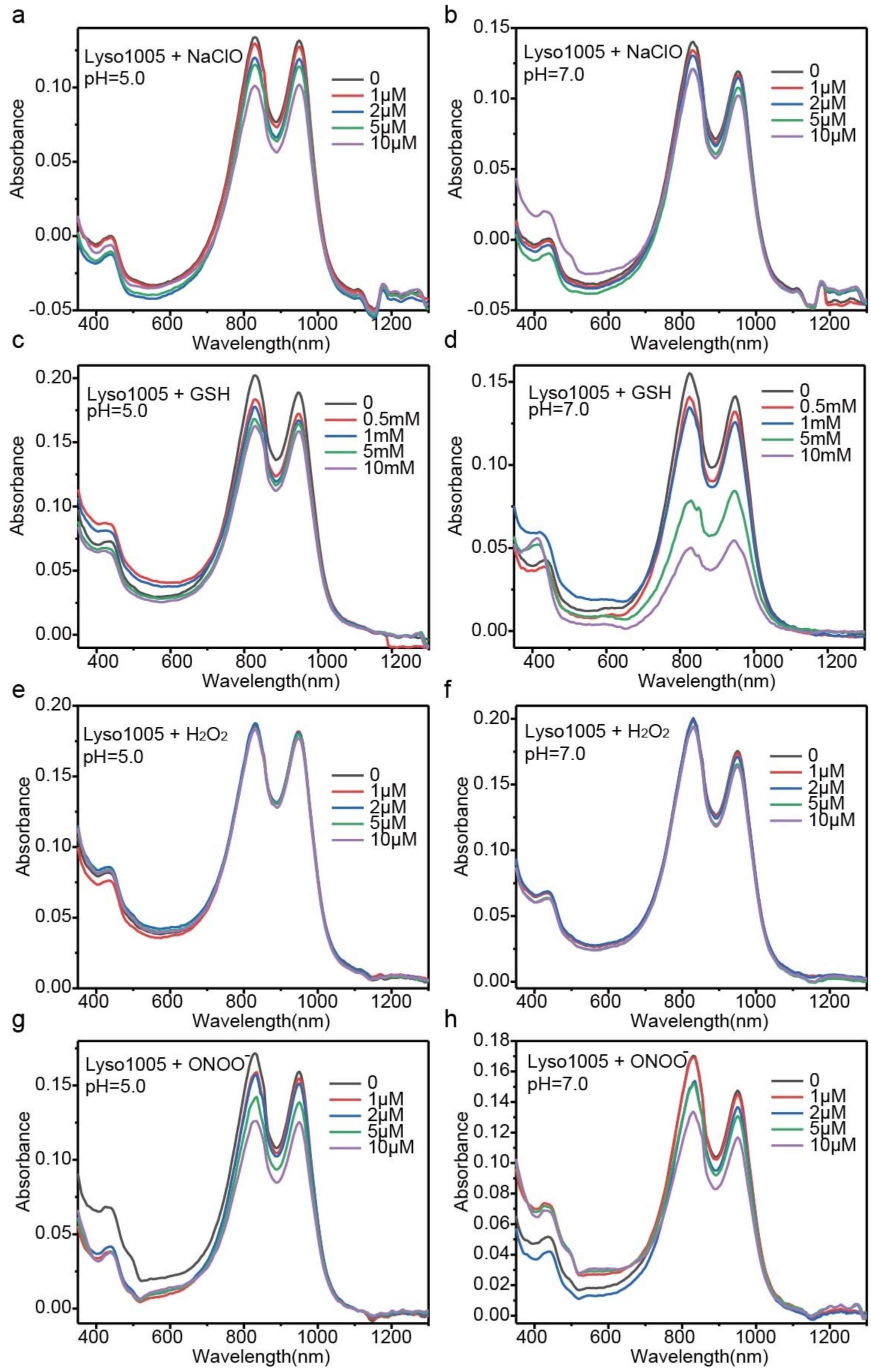

Figure S17. Chemical stability of Lyso1005. Absorption spectra of Lyso1005 against NaClO (a, b), GSH (c, d), H_2O_2 (e, f) and ONOO- (g, h) in the mixed solvent of acetonitrile and water (1:1, v/v, pH=7.0 or pH=5.0), spectra were recorded at 5 min after mixing with different amount of bioactive reagents solutions, the concentration of Lyso1005 was 10 μM. The increase in the shoulder peak ranging from 700 ~ 850 nm is due to the aggregation of dyes in aqueous solution.

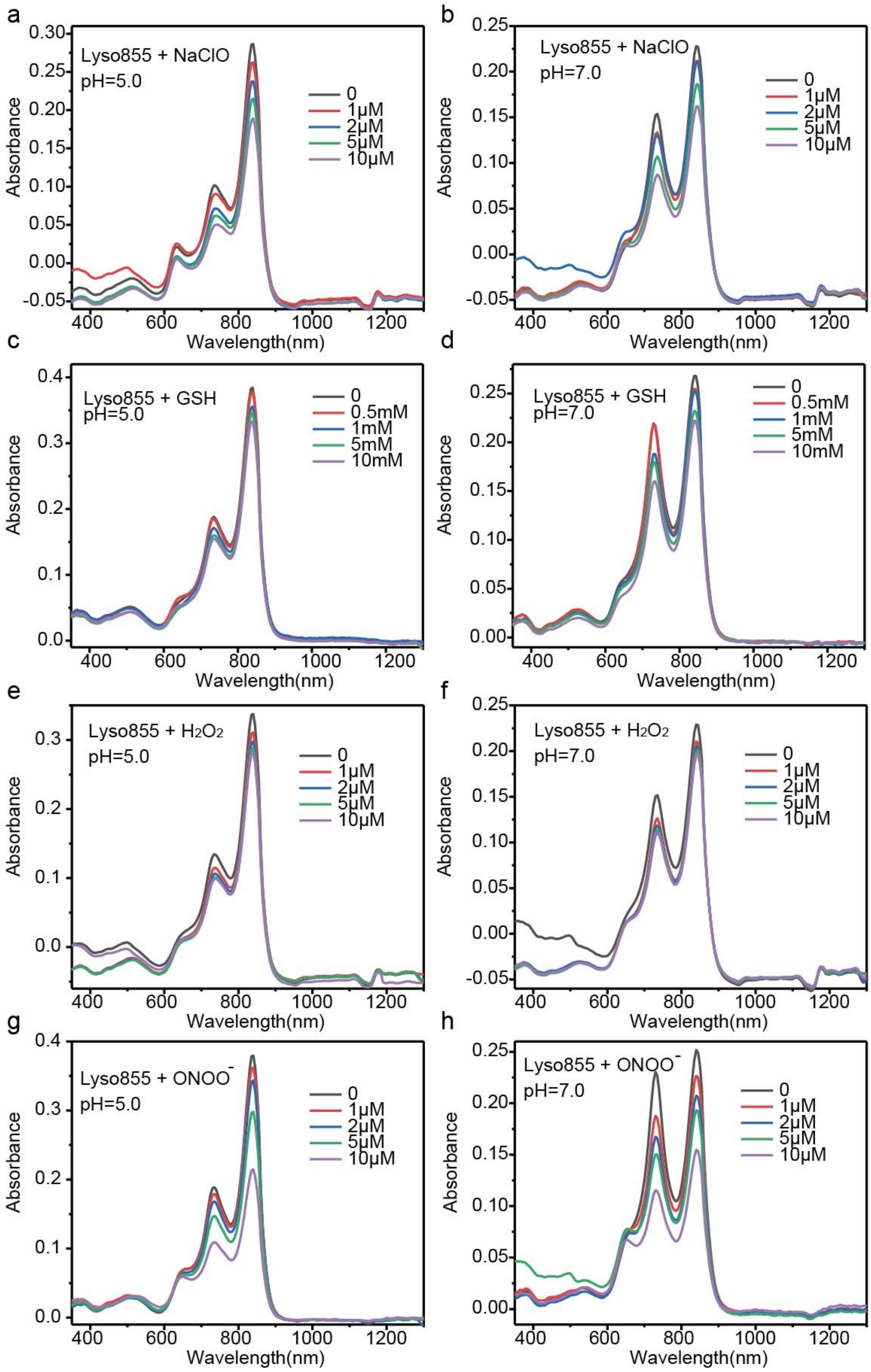

Figure S18. Chemical stability of Lyso855. Absorption spectra of Lyso855 against NaClO (a, b), GSH (c, d), H_2O_2 (e, f) and ONOO- (g, h) in the mixed solvent of acetonitrile and water (1:1, v/v, pH=7.0 or pH=5.0), spectra were recorded at 5 min after mixing with different amount of bioactive reagents solutions, the concentration of Lyso855 was 5 μM. The increase in the shoulder peak ranging from 650 ~ 800 nm is due to the aggregation of dyes in aqueous solution.

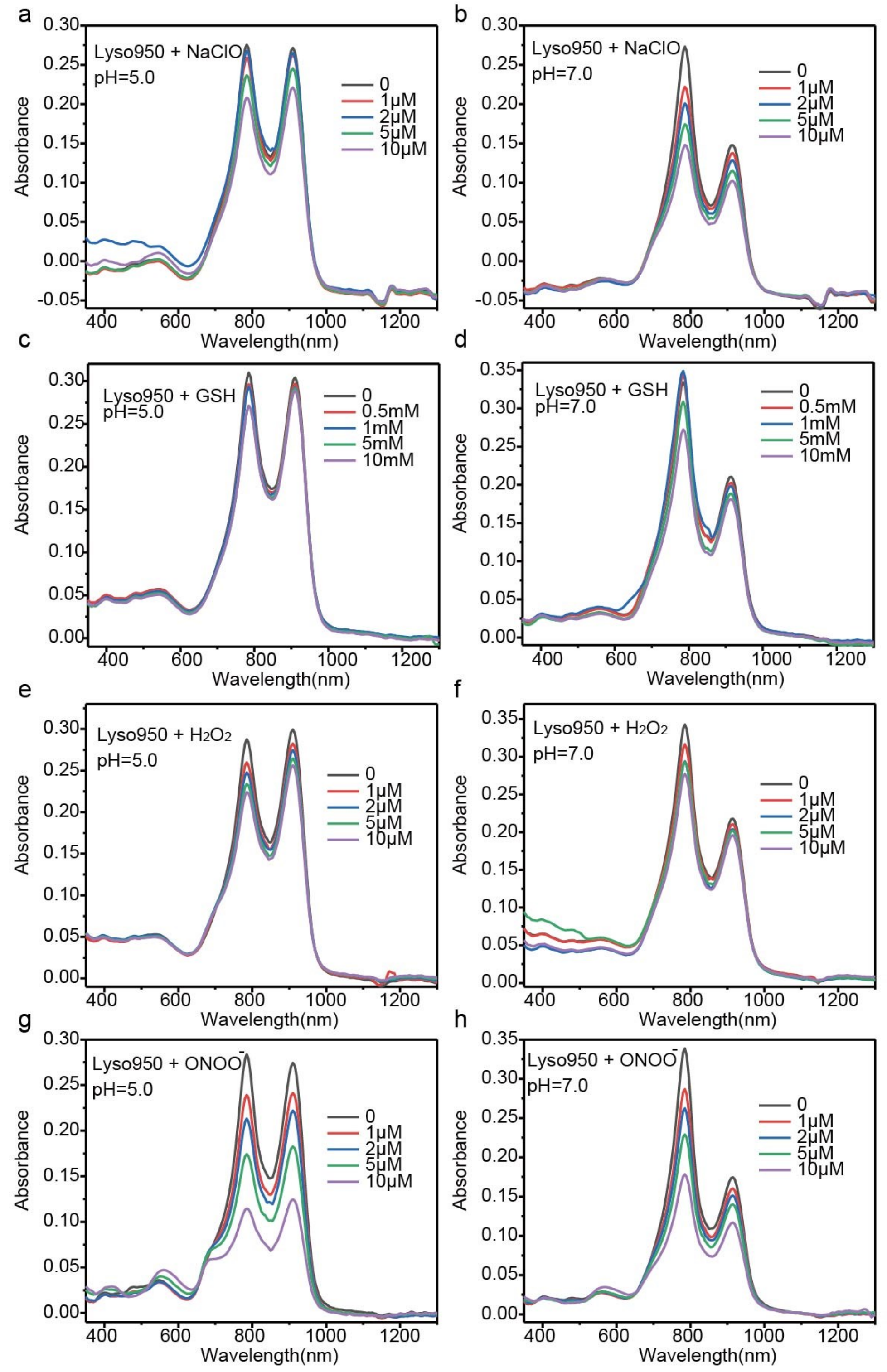

Figure S19. Chemical stability of Lyso950. Absorption spectra of Lyso950 against NaClO (a, b), GSH (c, d), H_2O_2 (e, f) and ONOO- (g, h) in the mixed solvent of acetonitrile and water (1:1, v/v, pH=7.0 or pH=5.0), spectra were at recorded 5 min after mixing with different amount of bioactive reagents solutions, the concentration of Lyso950 was 5 μM. The increase in the shoulder peak ranging from 700

~ 850 nm is due to the aggregation of dyes in aqueous solution.

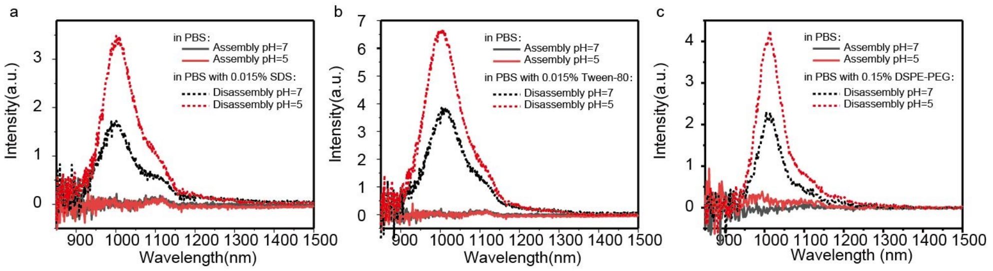

Figure S20. In vitro mimicking of the disaggregation property of CEAF-OMe. (a) The emission spectra of CEAF-OMe in pH=7.0 or pH=5.0 PBS with (dash lines) or without (solid line) 0.015% SDS. (b) The emission spectra of CEAF-OMe in pH=7.0 or pH=5.0 PBS with (dash lines) or without (solid line) 0.015% Tween-80. (c) The emission spectra of CEAF-OMe in pH=7.0 or pH=5.0 PBS with (dash lines) or without (solid line) 0.15% DSPE-PEG2000. The red lines represent pH=5 conditions, while the black lines represent the pH=7 conditions.

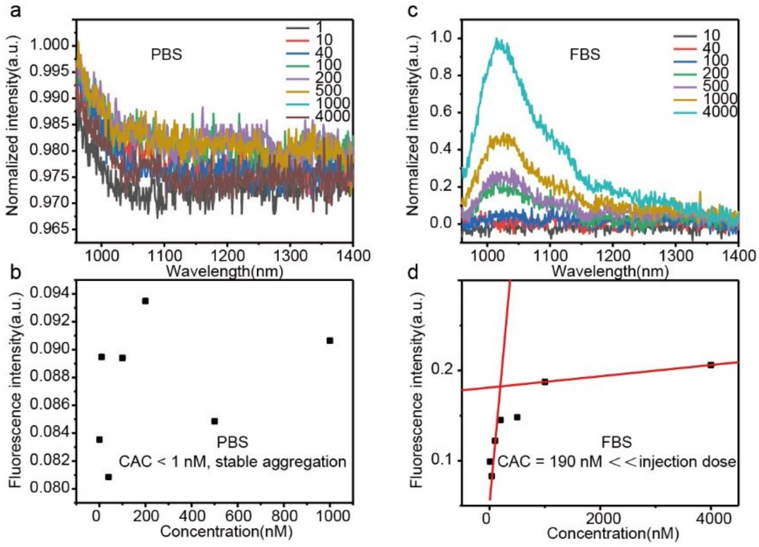

Figure S21. CAC determination of CEAF-OMe in PBS and FBS. Fluorescence spectra of CEAF-OMe of different concentrations (nM) in PBS (a) and FBS (c). Concentration-dependent changes of fluorescence intensities of CEAF-OMe in PBS (b) and FBS (d). The intersection point of the two red fitting lines determined the CAC values.

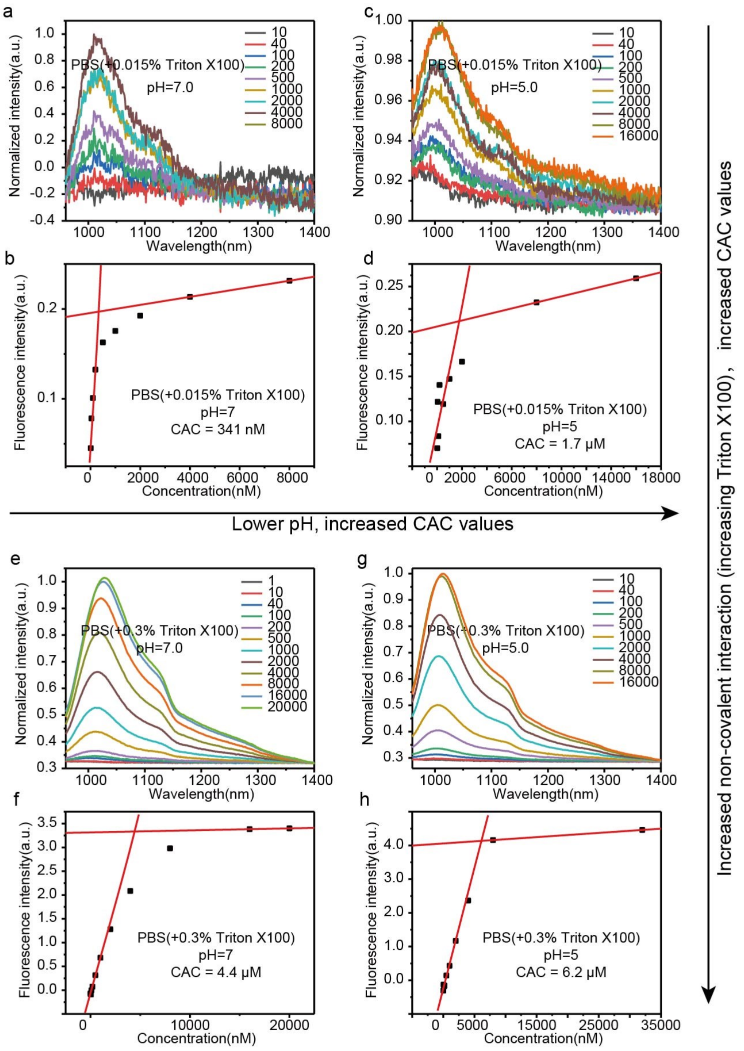

Figure S22. CAC determination of CEAF-OMe in PBS with different amount of Triton X100 at pH=7.0 and pH=5.0. Fluorescence spectra of CEAF-OMe of different concentrations (nM) in PBS with 0.015% Triton X100 (a, c) and PBS with 0.3% Triton X100 (e, g). Concentration-dependent changes of fluorescence intensities of CEAF-OMe in PBS with 0.015% Triton X100 (b, d) and PBS with 0.3% Triton X100 (f, h). The intersection point of the two red fitting lines determined the CAC values.

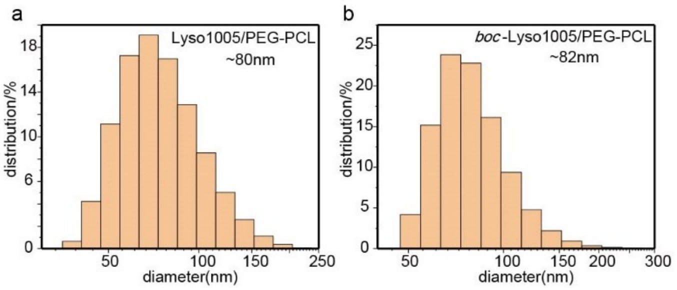

Figure S23. Hydrodynamic diameters of Lyso1005/PEG-PCL micelles (a) and *boc*-Lyso1005/PEG-PCL

micelles (b).

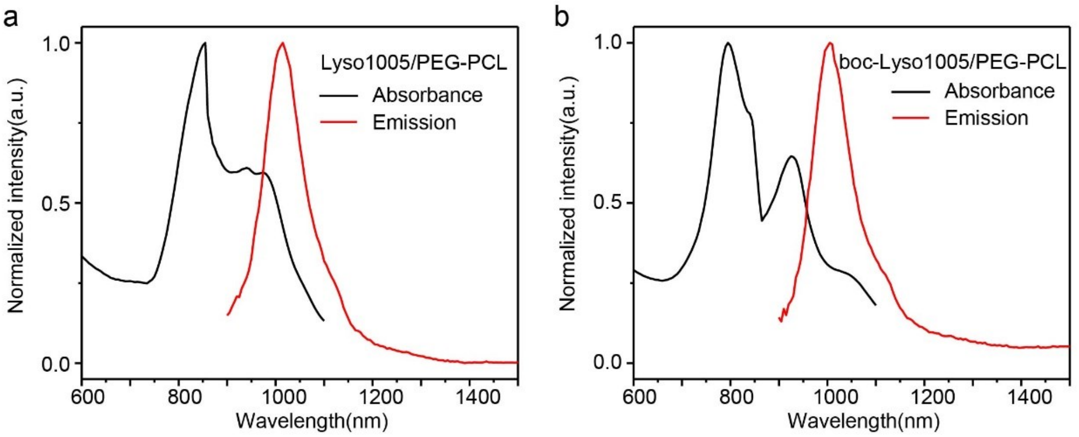

Figure S24. The absorption/emission spectra of Lyso1005/PEG-PCL (a) and boc-Lyso1005/PEG-PCL (b) in PBS. Excitation: 808 nm laser. Concentration: 10 µM.

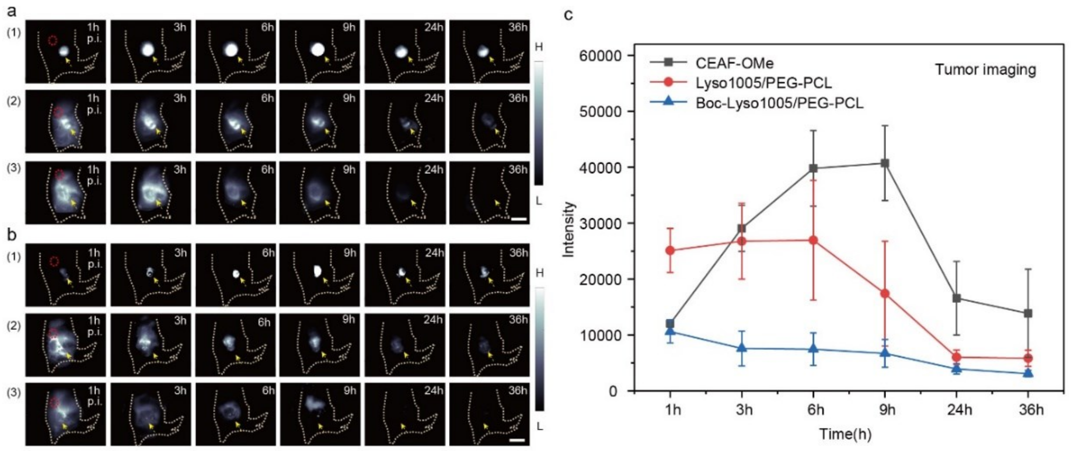

Figure S25. (a, b) In vivo NIR-II fluorescence images of mice (Mouse 2 and 3) bearing CT26 tumors at different time points after intravenous injection with CEAF-OMe nanoaggregates (1), Lyso1005/PEG-PCL micelles (2), and *boc*-Lyso1005/PEG-PCL micelles (3). Concentration: 50 µM, 200 µL. Scale bar, 2.5 cm. (c) Overall fluorescence intensity plot of tumor imaging. The mean intensity values of the tumor regions at different time points (n=3).

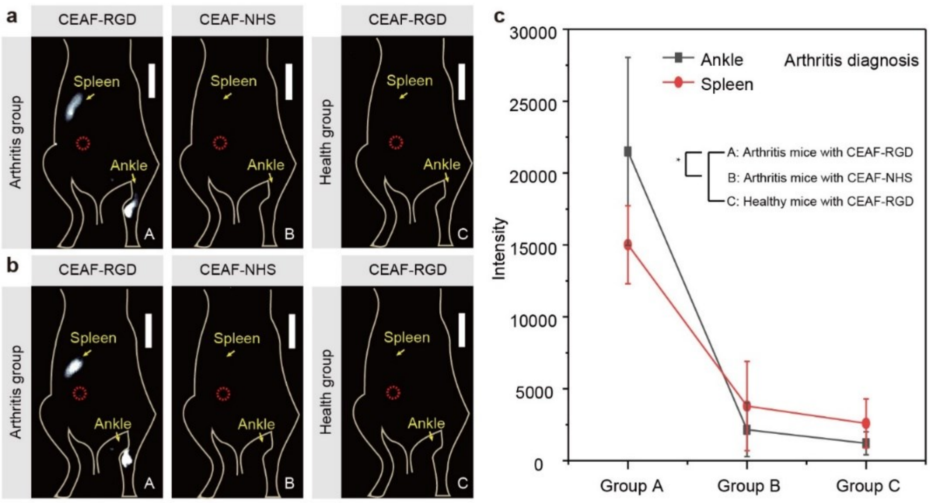

Figure S26. (a, b) In vivo NIR-II fluorescence images of arthritis mice (Mouse 2 and 3) injected with CEAF-RGD (group A) and CEAF-NHS (group B), and healthy mice injected with CEAF-RGD (group C), scale bar: 10 mm. Concentration: 50 μM, 200 μL. (c) Overall fluorescence intensity plot of arthritis diagnosis. The mean intensity values of the ankle and spleen regions at the group A, B and C (n=3); *p* values were analyzed between group A and group B, group A and group C by Student's two-sided t test (*$P < 0.05$).

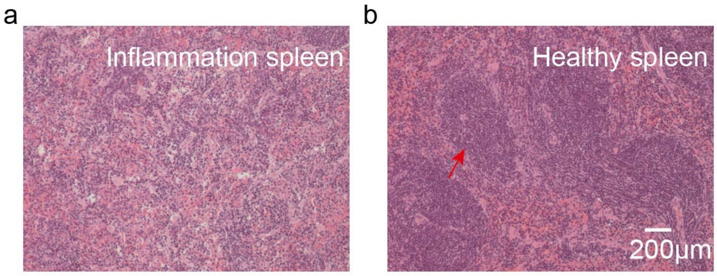

Figure S27. H&E staining images. Spleen samples of TA mouse (a) and healthy mouse (b). Reduction of the clusters of dark purple cells showing the production of inflammation. Scale bar: 200 μm. Repeated for 3 times in independent experiments.

Table S1. Photophysical Properties of dyes in different solvents.

Dye	solvent [a]	λ_{abs} [a](nm)	ε[a](M^{-1} cm^{-1})	λ_{fl}[a] (nm)	Stokes Shift (nm)	Φ_{fl}[a] (%)	$\varepsilon\Phi_{fl}$[a] (M^{-1} cm^{-1})
Lyso880	DCM	865	95631	902	37	1.91±0.009	1826.6 ±8.6
	EtOH	855	105185	900	45	0.49±0.002	515.4 ±2.1

	MeCN/PBS (pH=7.0)	852	15786	877	25	0.14±0.006	22.1 ±0.9
Lyso880 -2H^{+}	DCM	865	127372	904	39	3.04±0.014	3872.1± 17.8
	EtOH	855	109462	891	36	1.59±0.007	1740.4± 7.7
	MeCN/PBS (pH=5.0)	854	46860	880	26	0.51±0.009	239.0 ±4.2
Lyso1005	DCM	980	58129	1017	37	0.20±0.001	116.3 ±0.6
	EtOH	965	57628	995	30	0.06±0.001	34.6± 0.6
	MeCN/PBS (pH=7.0)	949	13262	987	38	0.03±0.001	4.0 ±0.1
Lyso1005 -2H^{+}	DCM	970	45777	1008	38	0.24±0.001	109.9± 0.5
	EtOH	955	47948	987	32	0.21±0.001	100.7± 0.5
	MeCN/PBS (pH=5.0)	943	18429	984	41	0.04±0.012	7.4 ±2.2
Lyso855	DCM	855	104083	892	37	3.37±0.015	3507.6±15.6
	EtOH	845	125564	884	39	2.21±0.019	2775.0± 23.9
	MeCN/PBS (pH=7.0)	844	51280	876	32	1.67±0.081	856.4± 41.5
Lyso855 -2H^{+}	DCM	855	151660	890	35	3.32±0.015	5035.1± 22.7
	EtOH	840	113051	875	35	2.28±0.063	2577.6± 71.2
	MeCN/PBS (pH=5.0)	838	72408	872	34	1.72±0.049	1245.4± 35.5
Lyso950	DCM	930	120454	976	46	1.34±0.006	1614.1± 7.2
	EtOH	925	126339	967	42	1.16±0.005	1465.5± 6.3
	MeCN/PBS (pH=7.0)	914	36870	950	36	0.40±0.021	147.5 ±7.7
Lyso950 -2H^{+}	DCM	930	116648	972	42	1.76±0.008	2053.0± 9.3
	EtOH	915	118039	959	44	1.21±0.005	1428.3± 5.9
	MeCN/PBS (pH=5.0)	910	60103	949	39	0.46±0.023	276.5± 13.8
Boc-Lyso1005	DCM	975	104880	1017	42	0.38±0.006	399.0±6.3
	EtOH	960	58563	997	37	0.13±0.001	76.0±0.6
	MeCN/PBS (pH=7.0)	956	14103	995	39	0.02±0.001	2.8± 0.1

[a]For determination of the fluorescence quantum efficiency (Φ_{fl}), IR-26 in dichloroethane ($\Phi_{fl,r}$= 0.05%) was used as a fluorescence standard. DCM: dichloromethane, EtOH: ethyl alcohol, MeCN/PBS: acetonitrile and PBS (1:1, v/v).

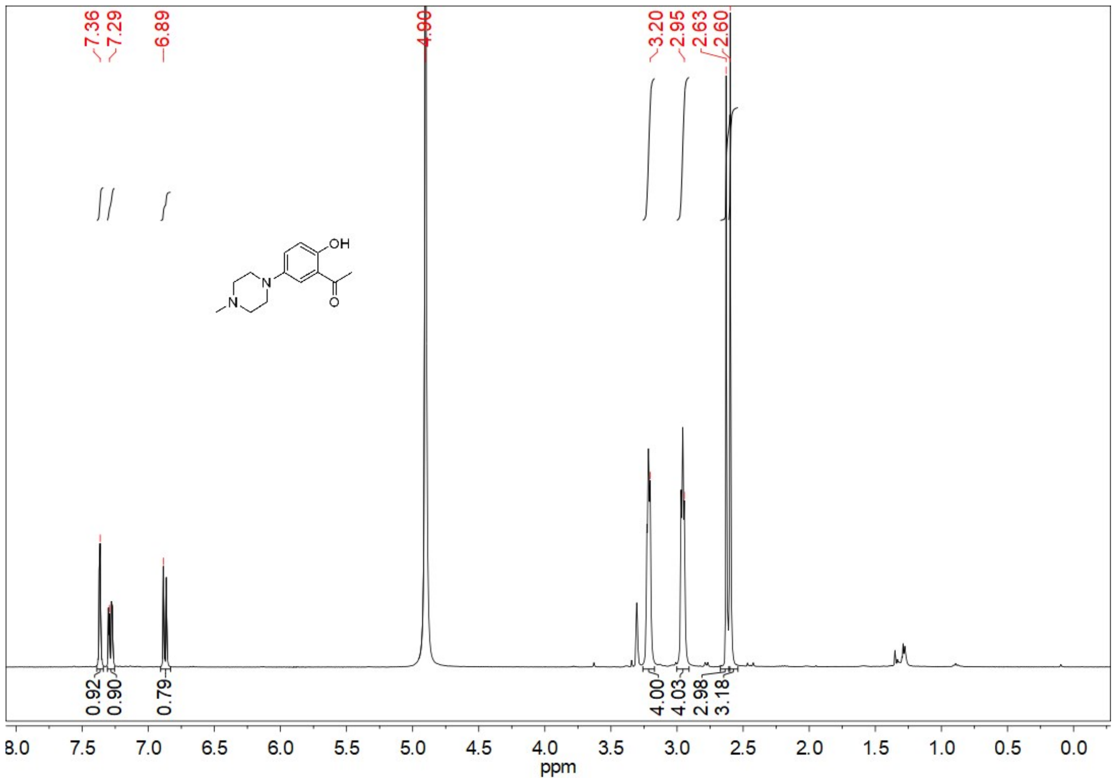

Figure S28. ^{1}H-NMR spectrum of A2 in $CDCl_3$.

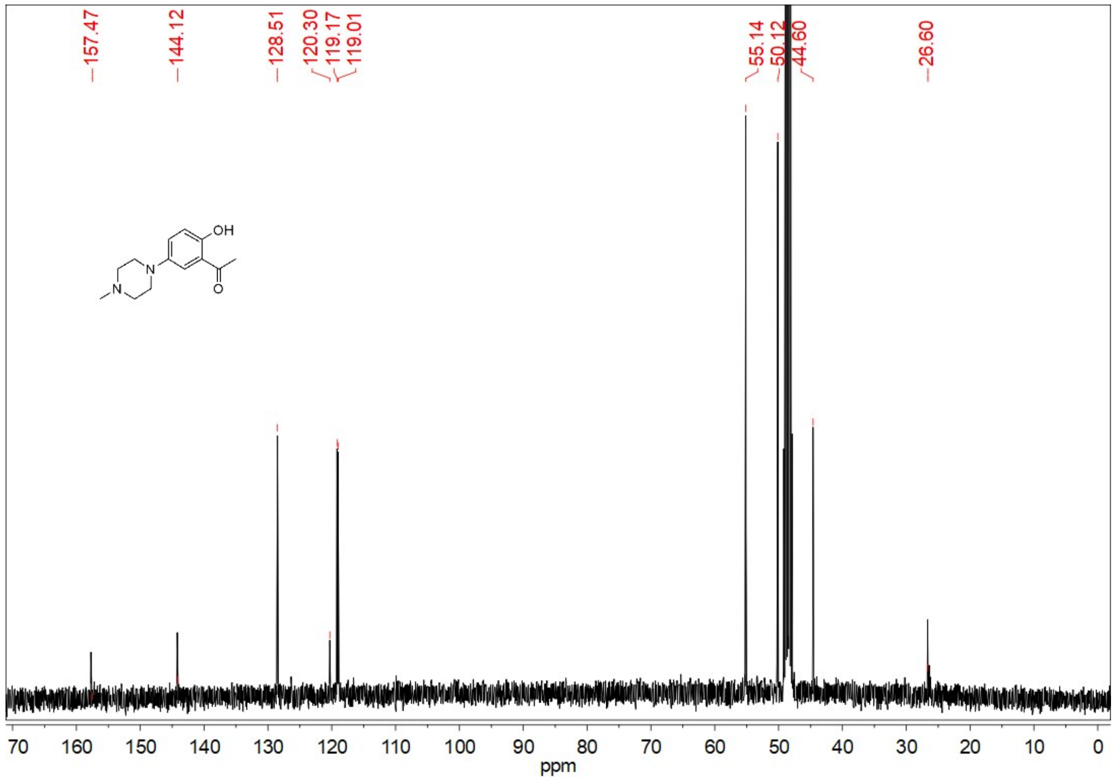

Figure S29. ^{13}C-NMR spectrum of A2 in $CDCl_3$.

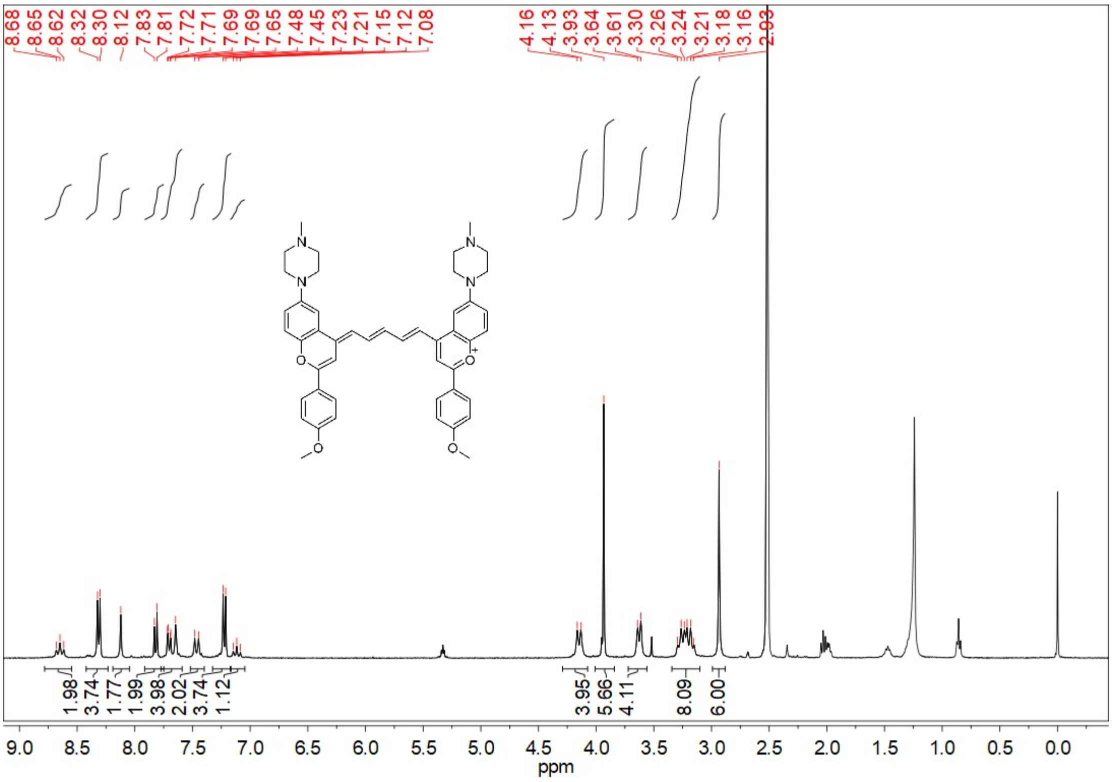

Figure S30. ^{1}H-NMR spectrum of Lyso880 in DMSO-D6.

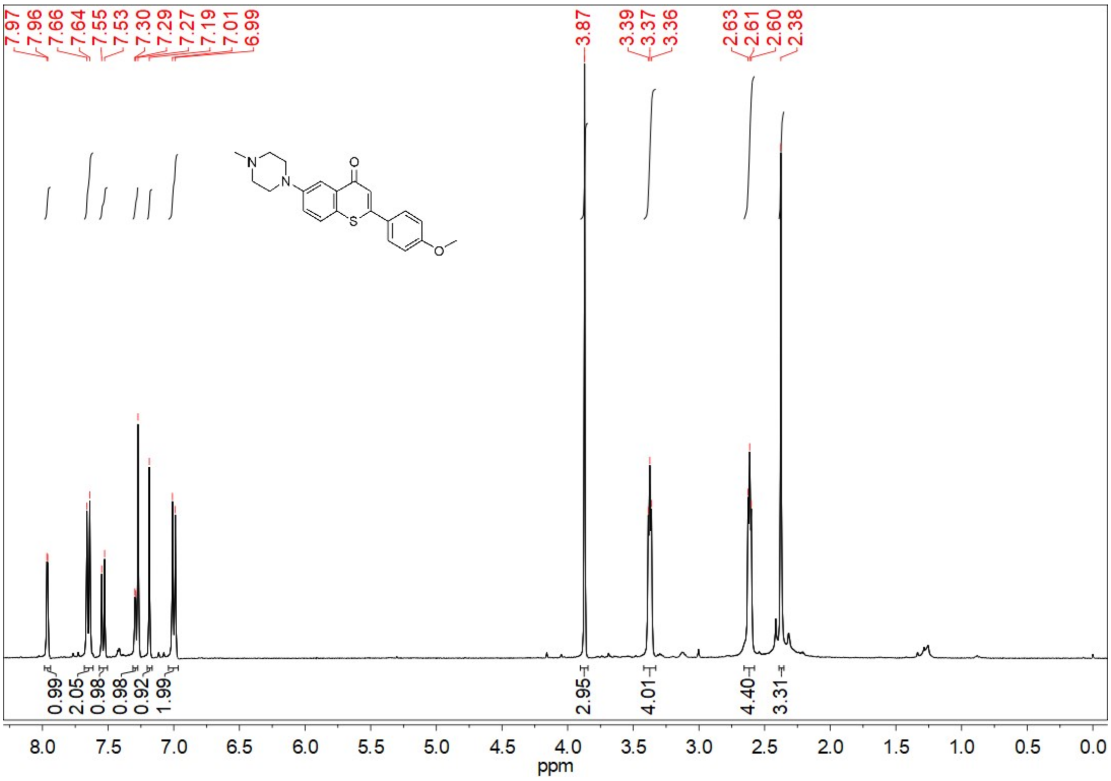

Figure S31. ^{1}H-NMR spectrum of B3 in $CDCl_3$.

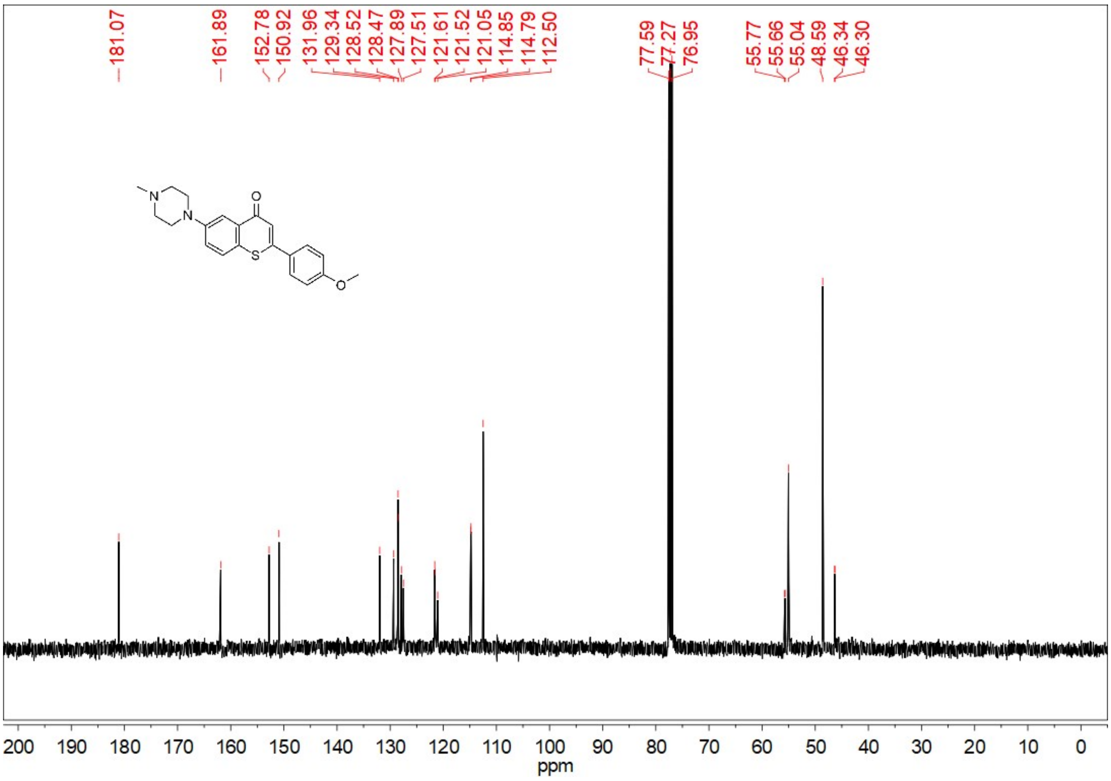

Figure S32. ^{13}C-NMR spectrum of B3 in $CDCl_3$.

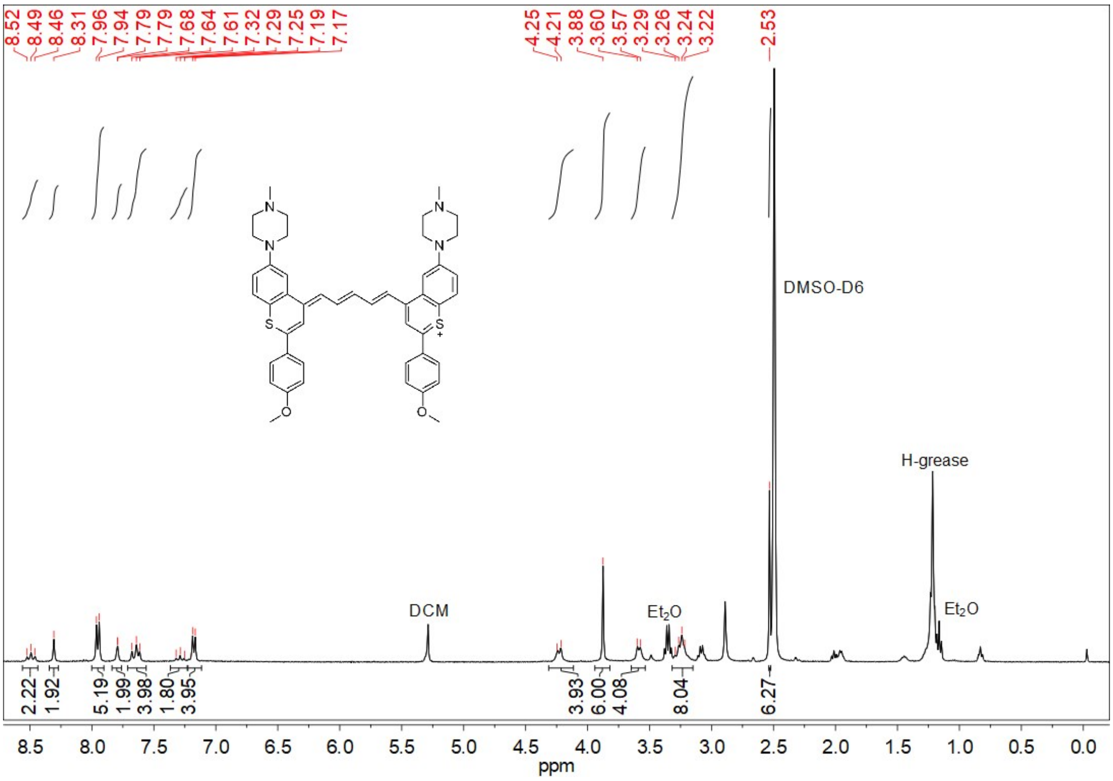

Figure S33. ^{1}H-NMR spectrum of Lyso1005 in DMSO-D6.

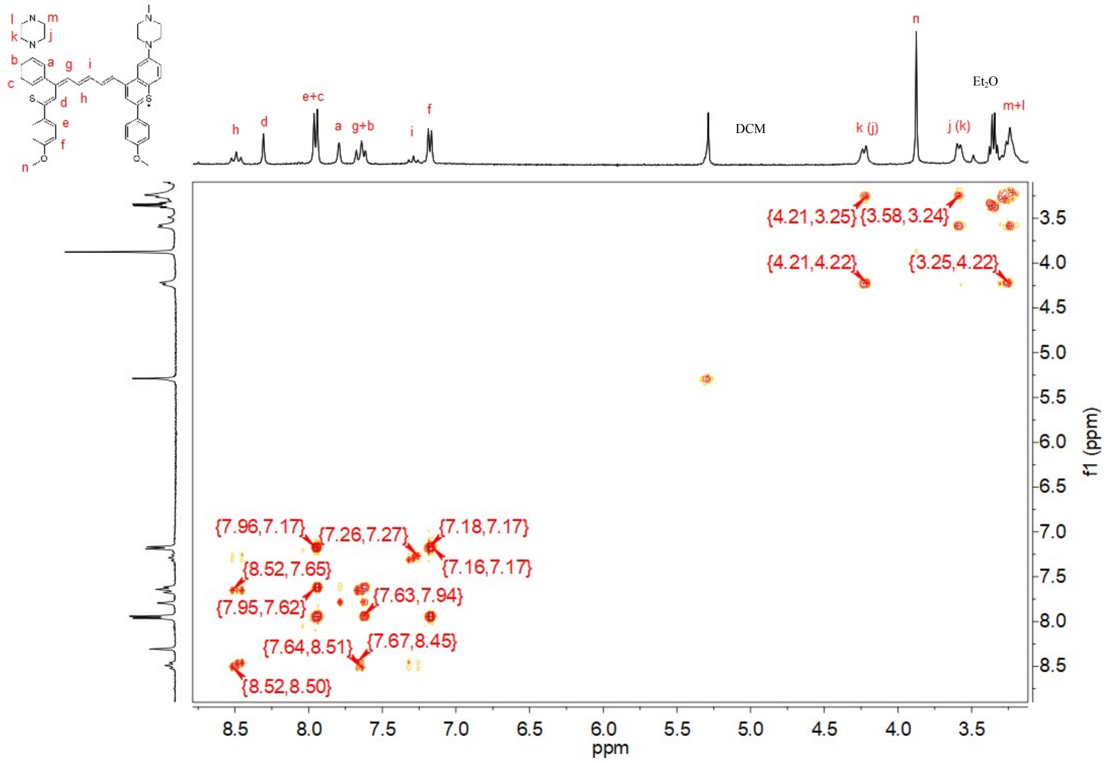

Figure S34. ^1H-^1H COSY spectrum of Lyso1005 in DMSO-D6.

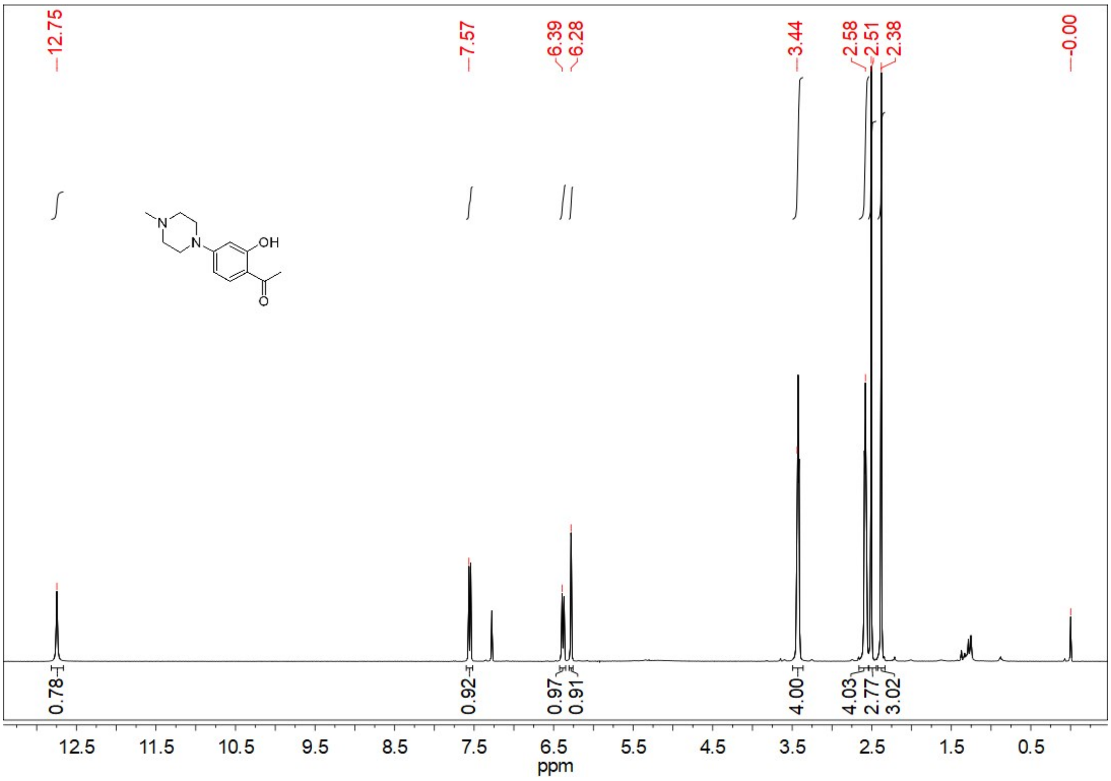

Figure S35. ^1H-NMR spectrum of C2 in $CDCl_3$.

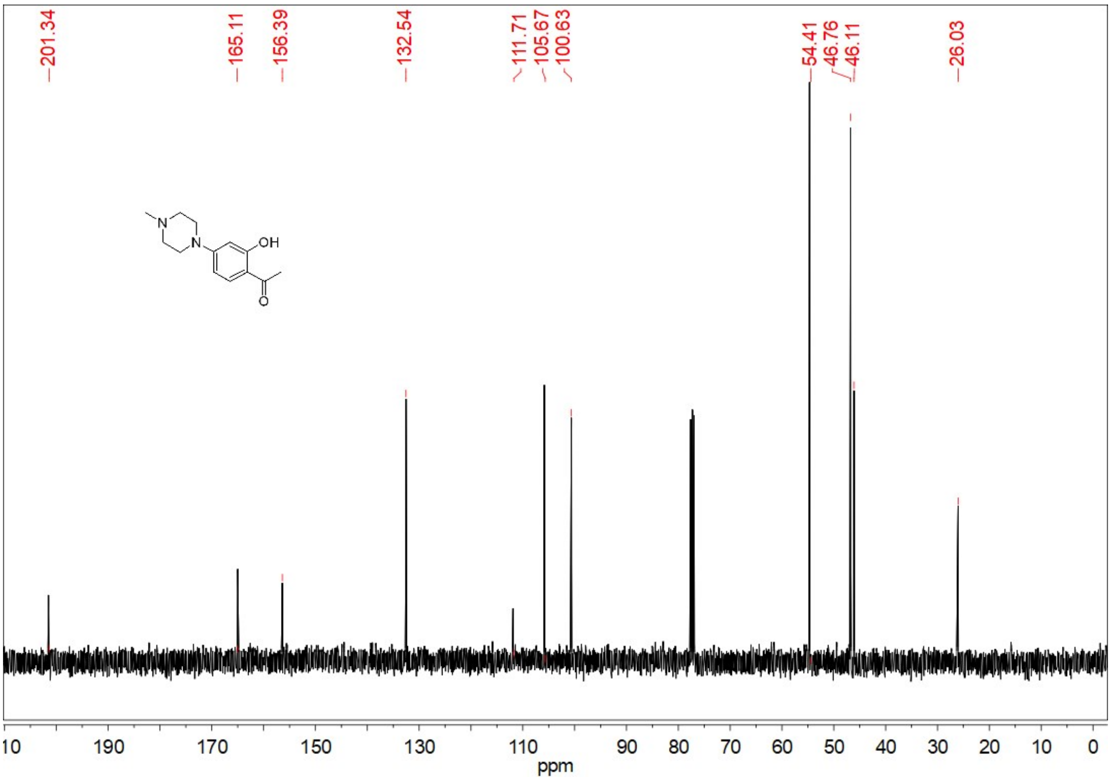

Figure S36. ^{13}C-NMR spectrum of C2 in $CDCl_3$.

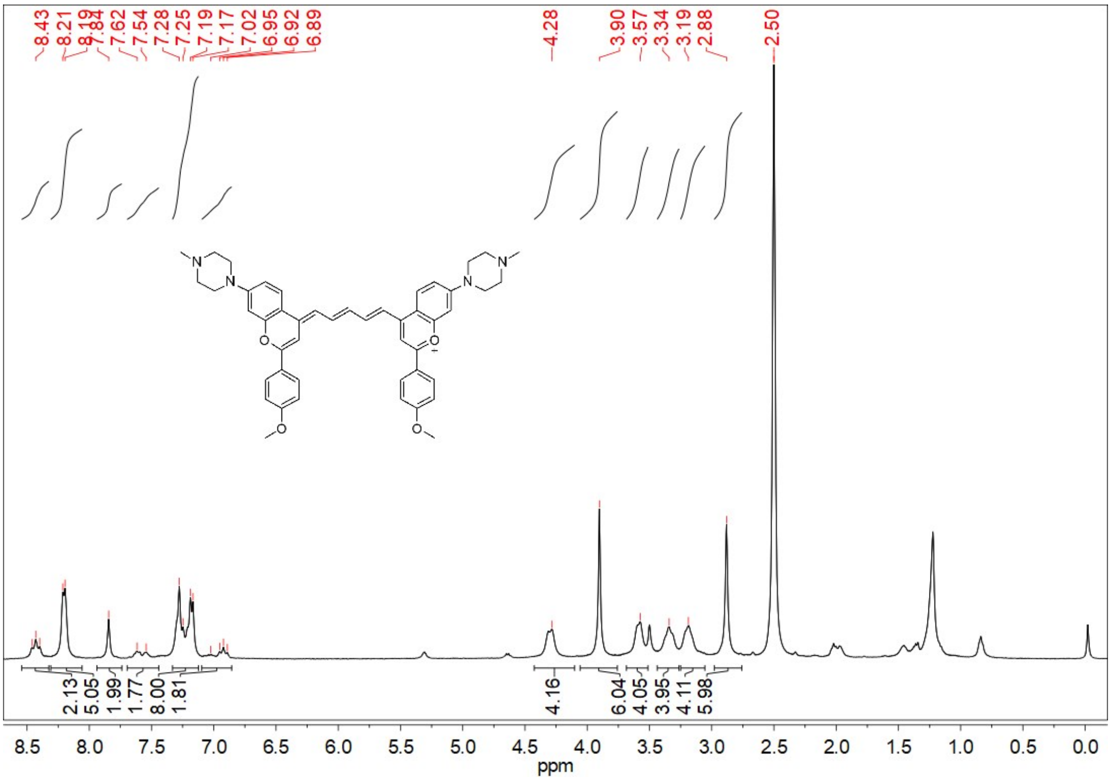

Figure S37. ^{1}H-NMR spectrum of Lyso855 in DMSO-D6.

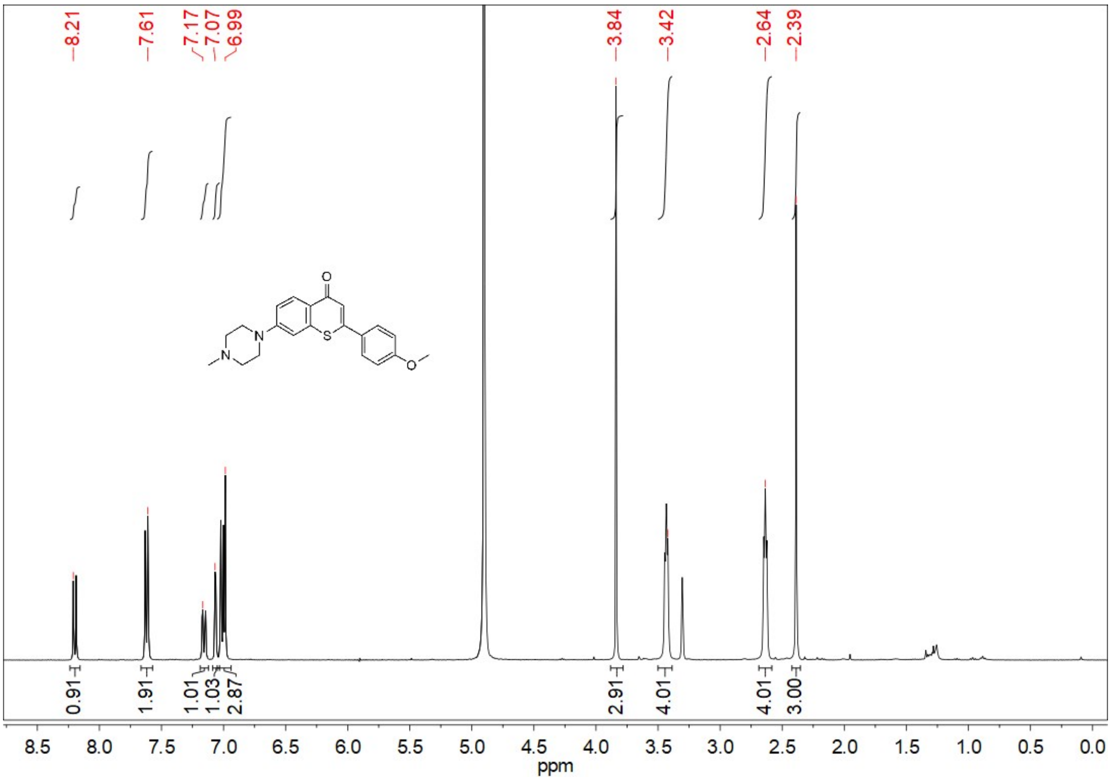

Figure S38. ^{1}H-NMR spectrum of D3 in CD_3OD.

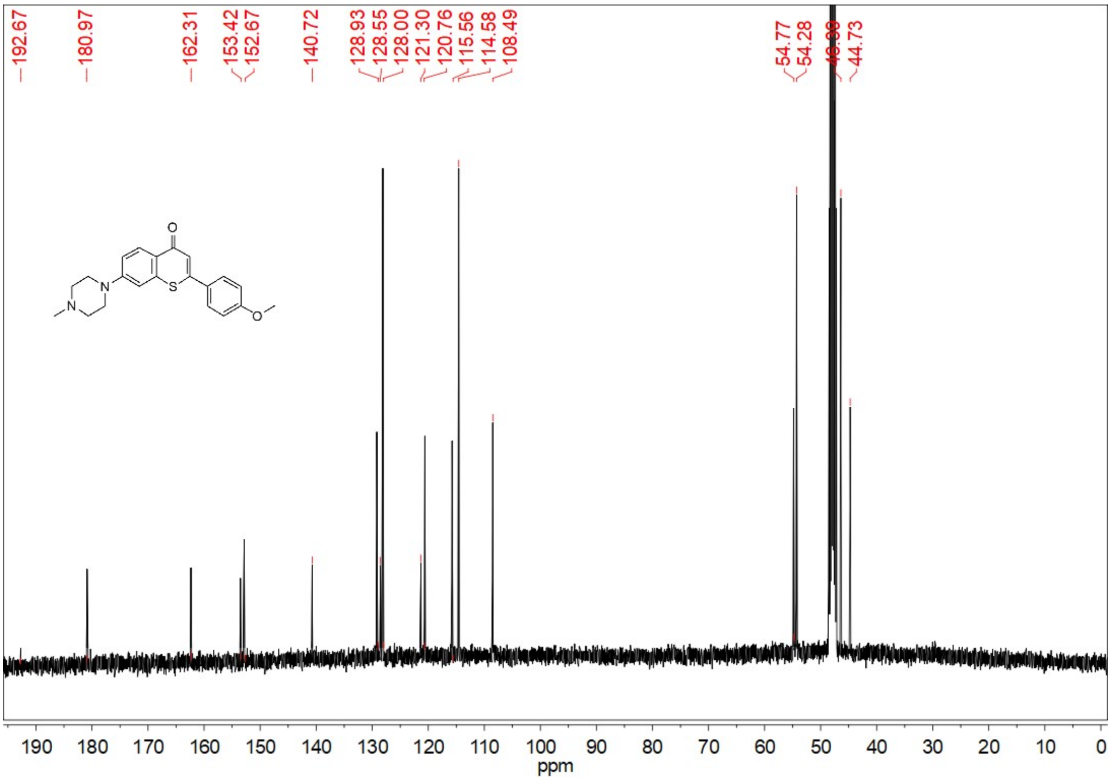

Figure S39. ^{13}C-NMR spectrum of D3 in CD_3OD.

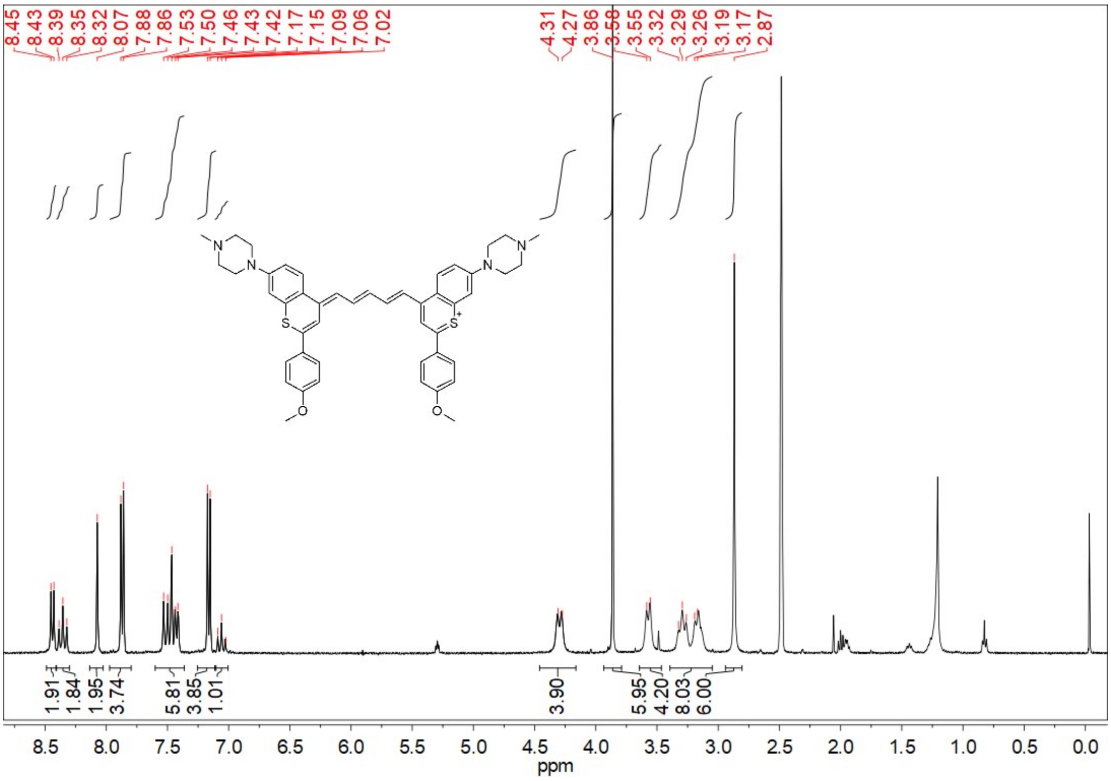

Figure S40. ^{1}H-NMR spectrum of Lyso950 in DMSO-D6.

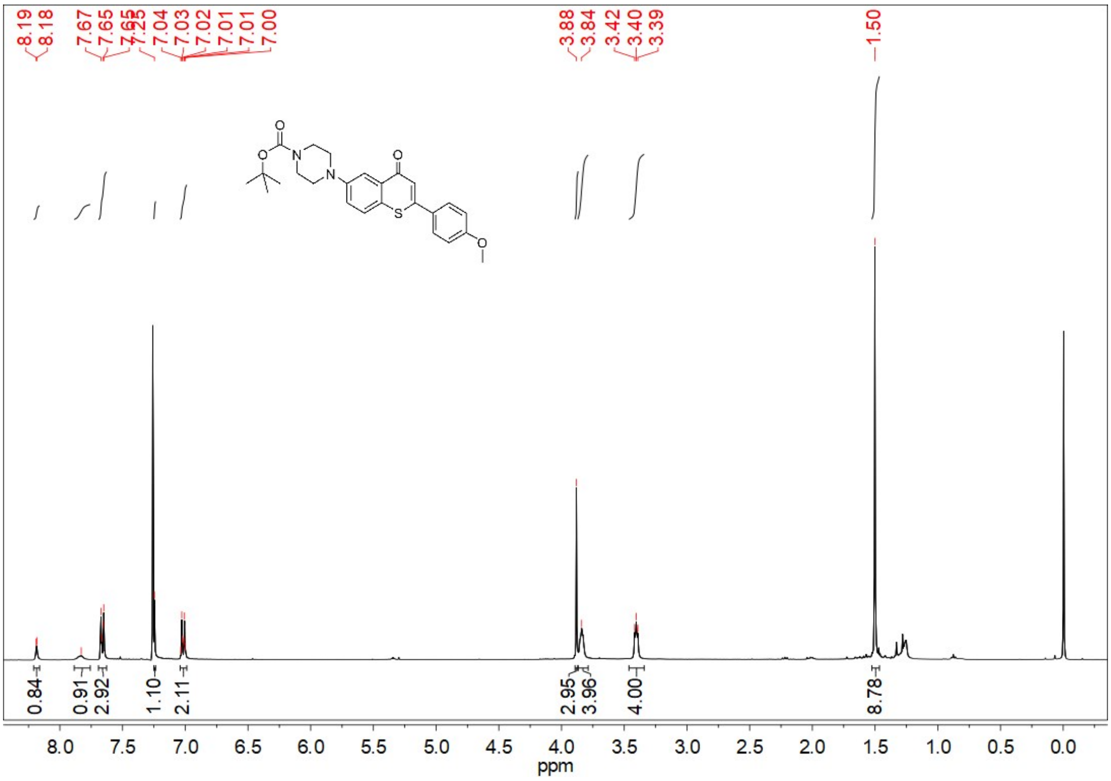

Figure S41. ^{1}H-NMR spectrum of E1 in $CDCl_3$.

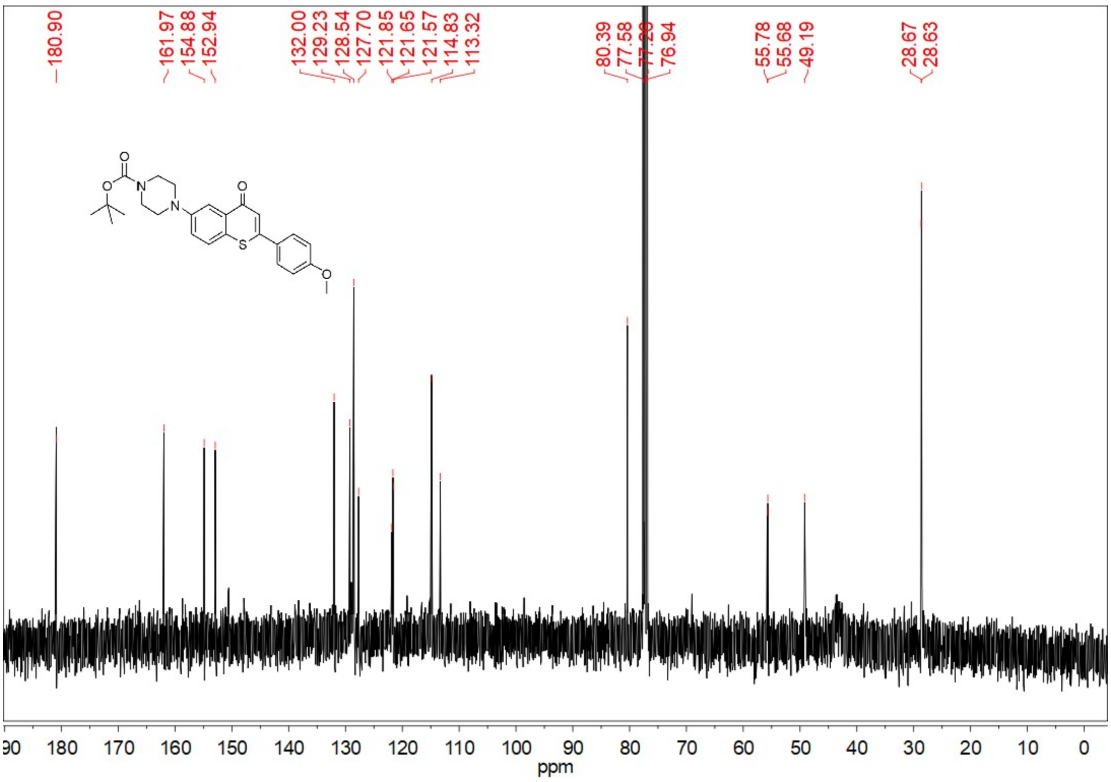

Figure S42. ^{13}C-NMR spectrum of E1 in $CDCl_3$.

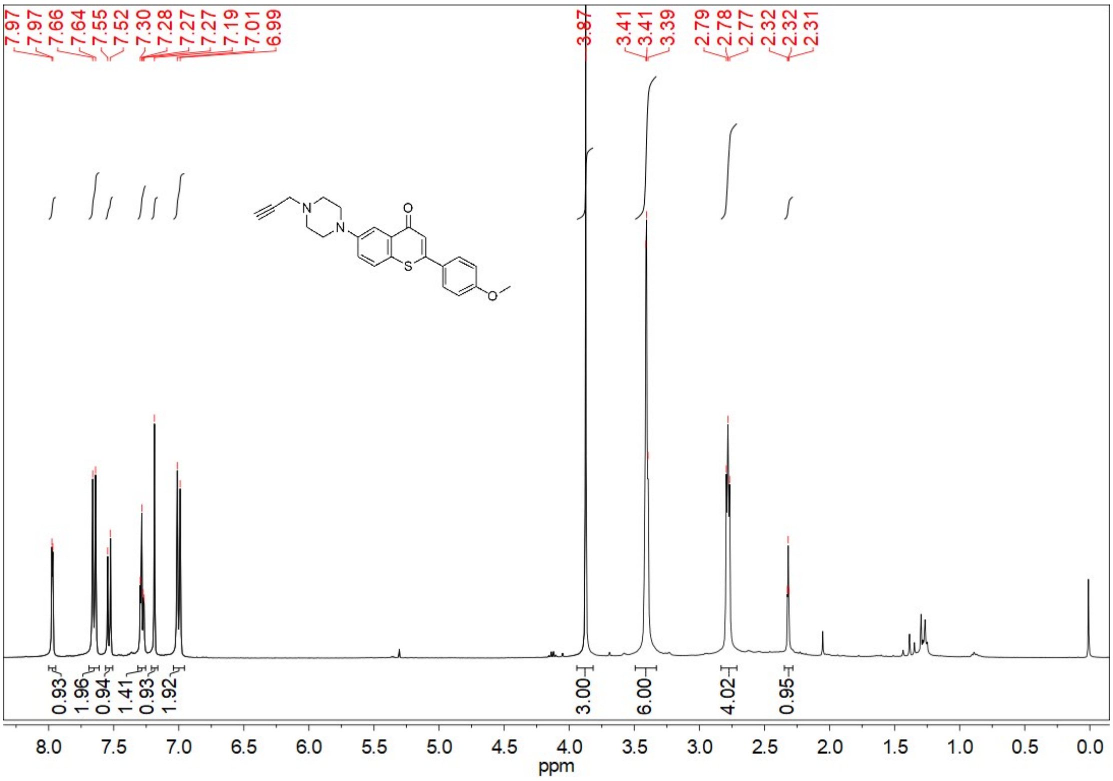

Figure S43. ^{1}H-NMR spectrum of E3 in $CDCl_3$.

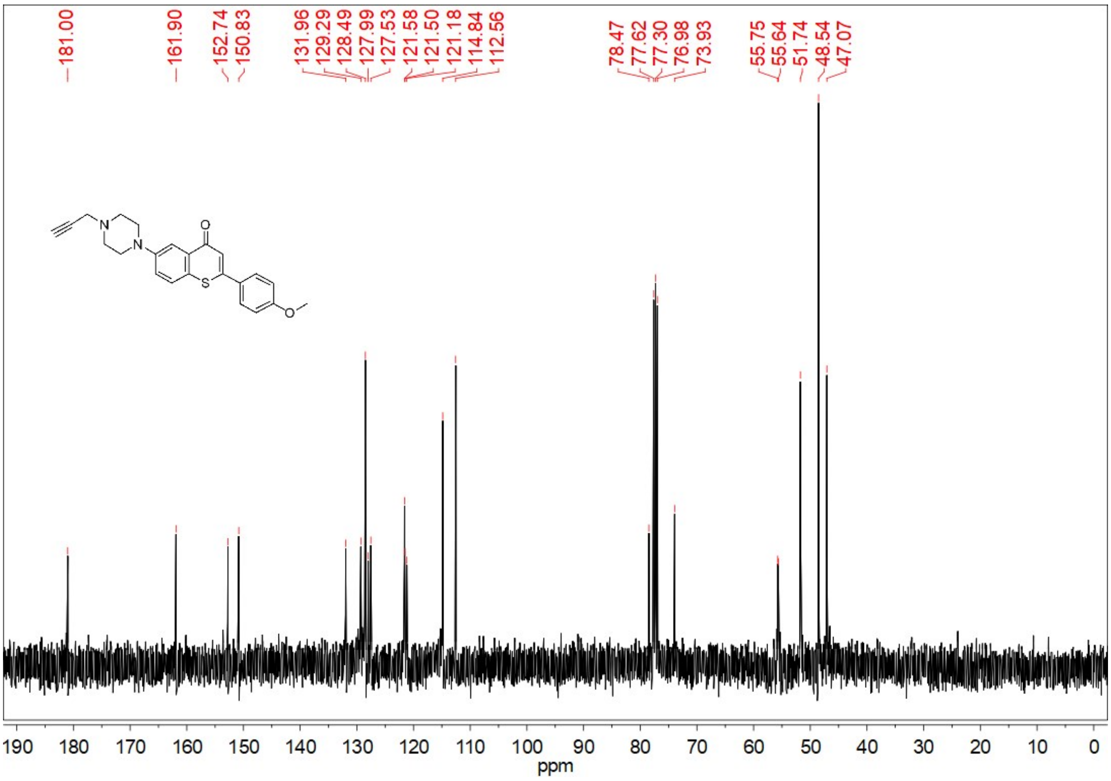

Figure S44. ^{13}C-NMR spectrum of E3 in $CDCl_3$.

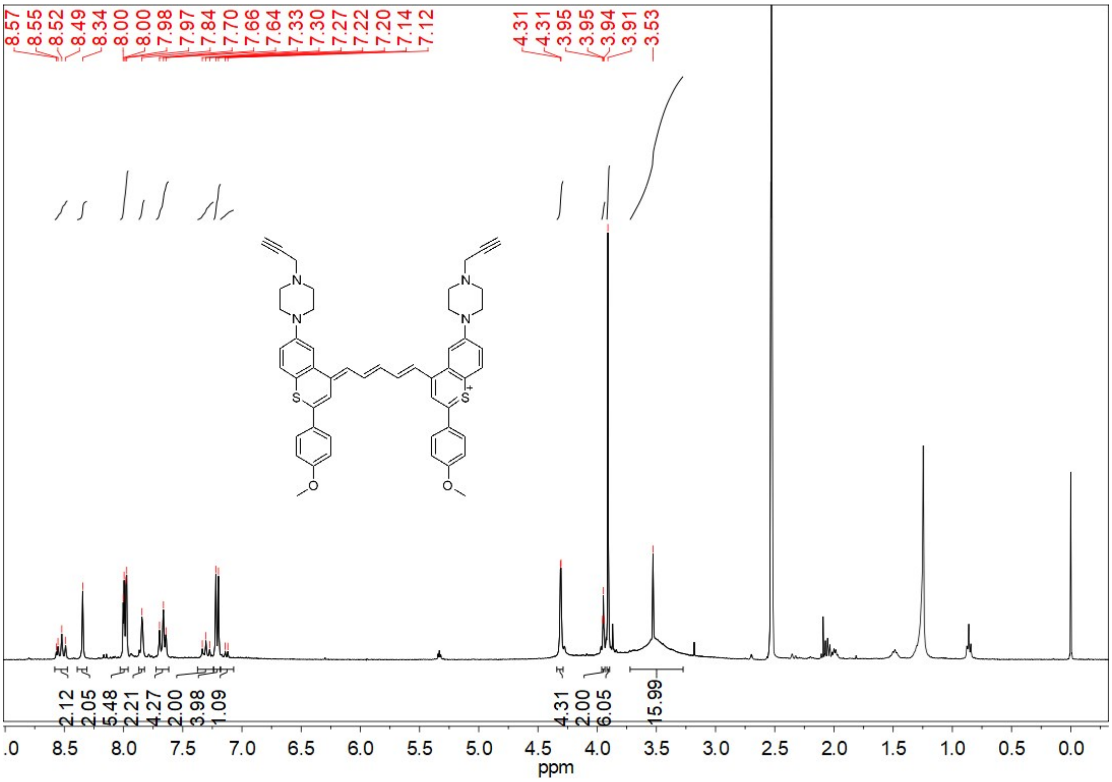

Figure S45. ^{1}H-NMR spectrum of *prop*-Lyso1005 in DMSO-D6.

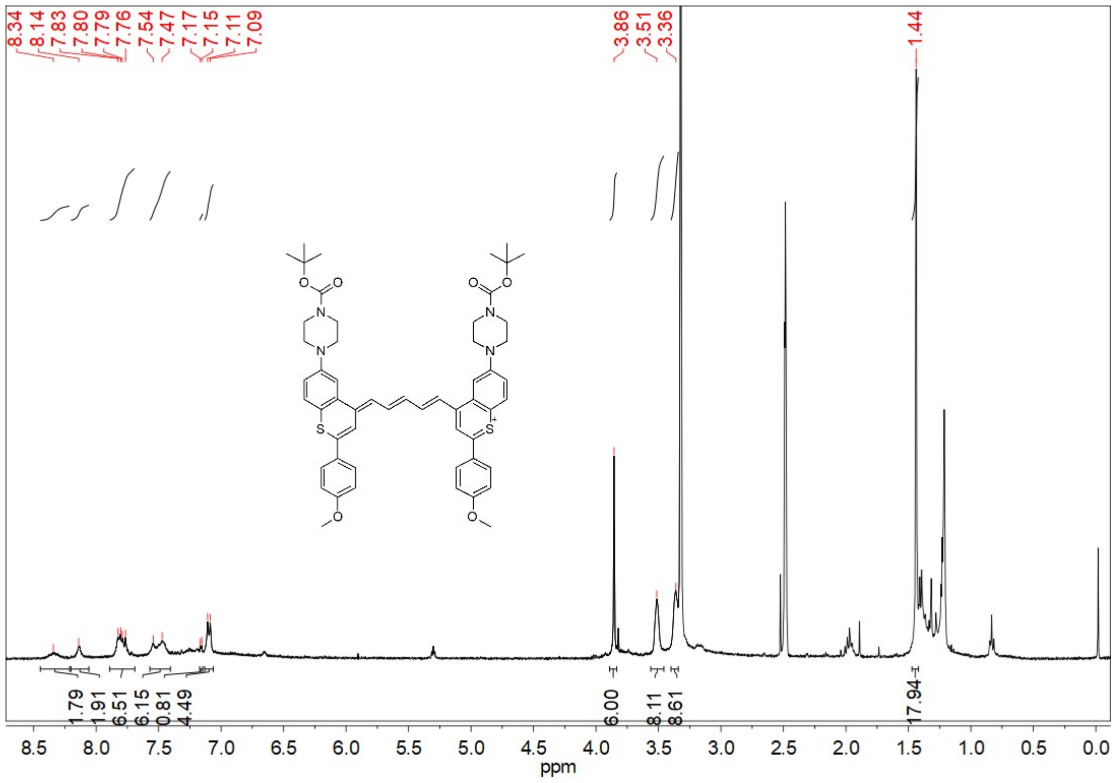

Figure S46. ^{1}H-NMR spectrum of *boc*-Lyso1005 in DMSO-D6.

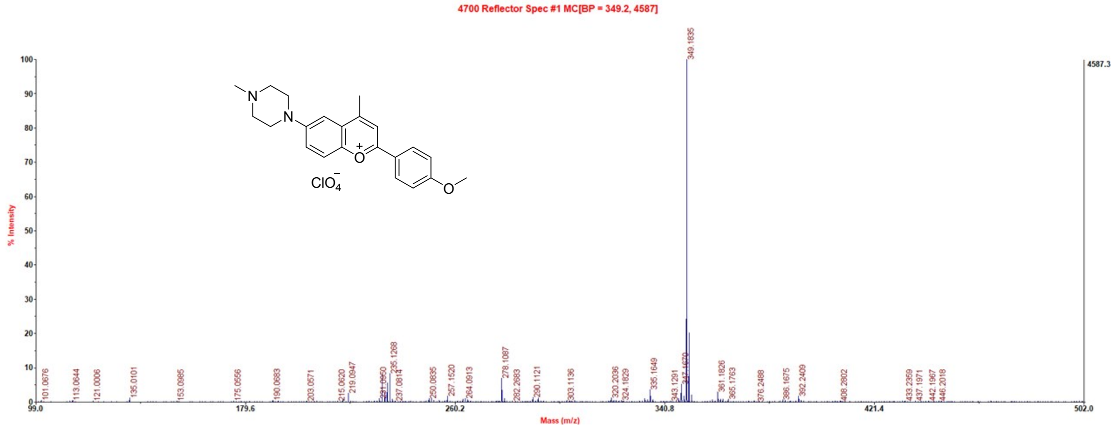

Figure S47. Maldi-Tof/Tof-MS spectrum of A3.

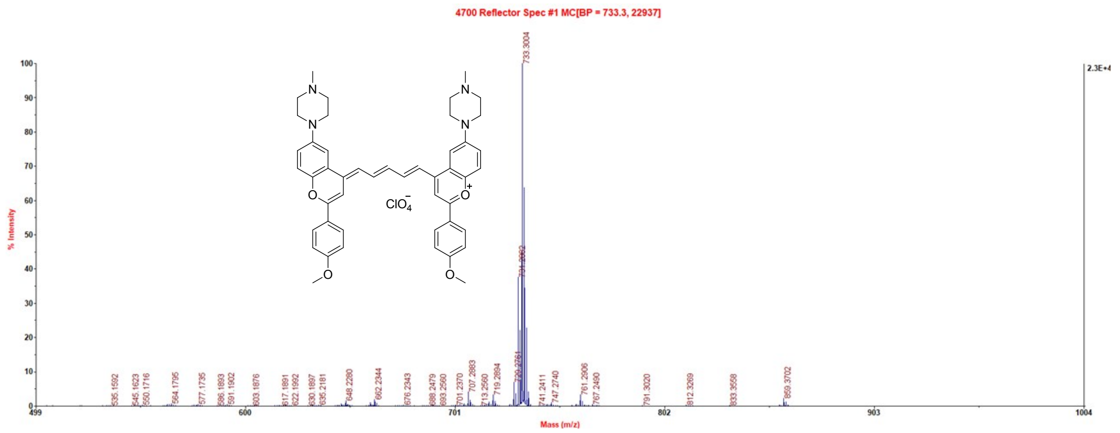

Figure S48. Maldi-Tof/Tof-MS spectrum of Lyso880.

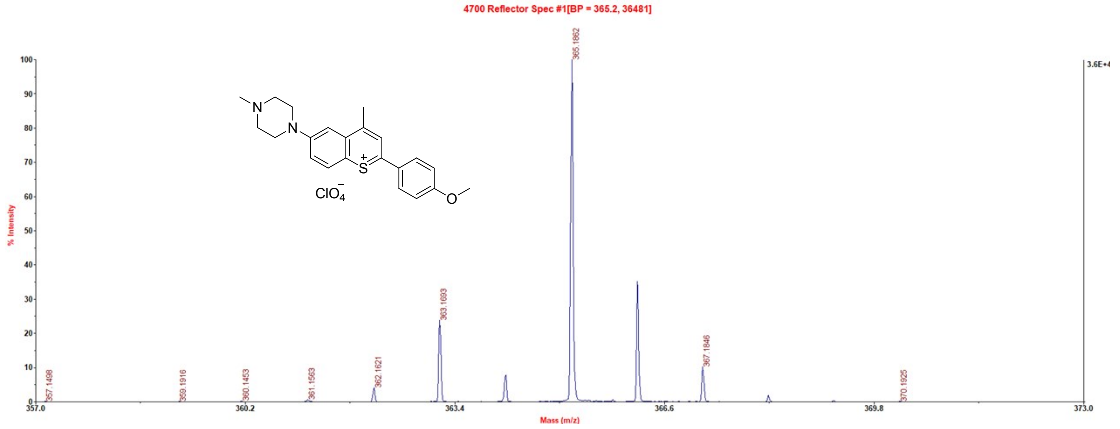

Figure S49. Maldi-Tof/Tof-MS spectrum of B4.

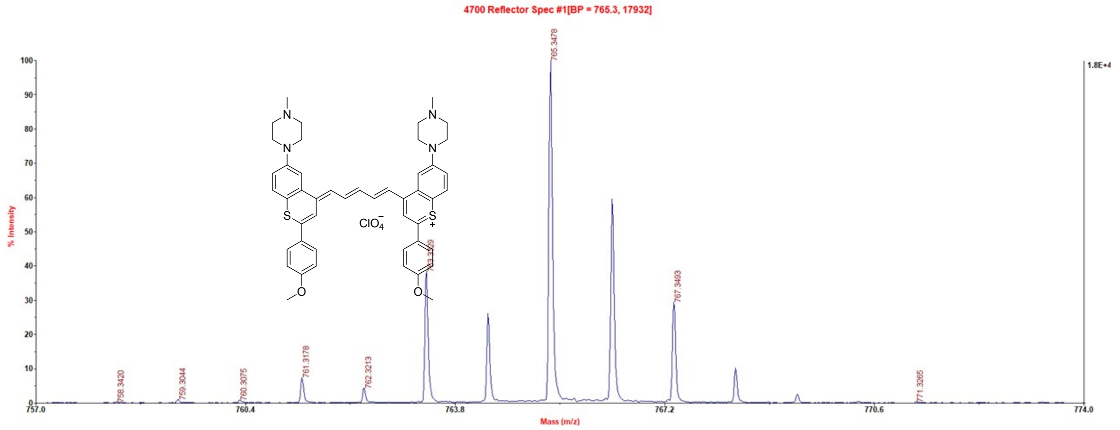

Figure S50. Maldi-Tof/Tof-MS spectrum of Lyso1005.

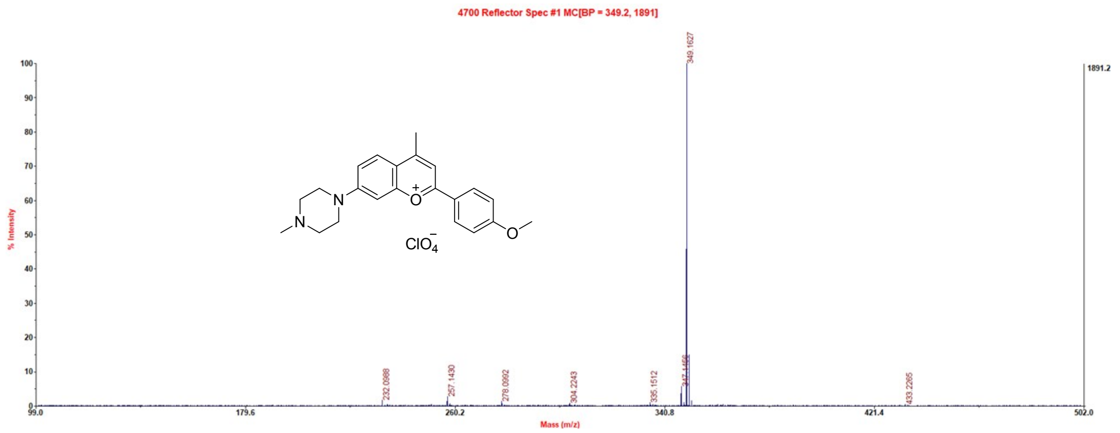

Figure S51. Maldi-Tof/Tof-MS spectrum of C3.

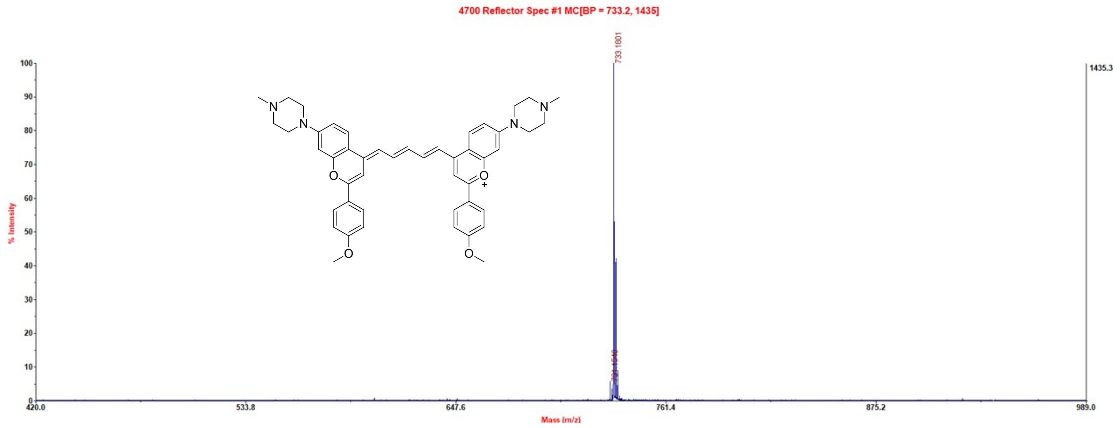

Figure S52. Maldi-Tof/Tof-MS spectrum of Lyso855.

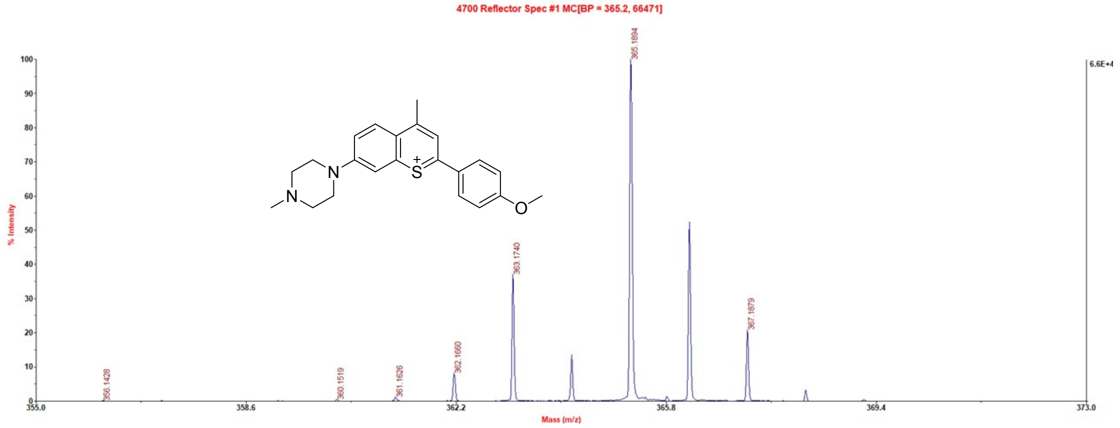

Figure S53. Maldi-Tof/Tof-MS spectrum of D4.

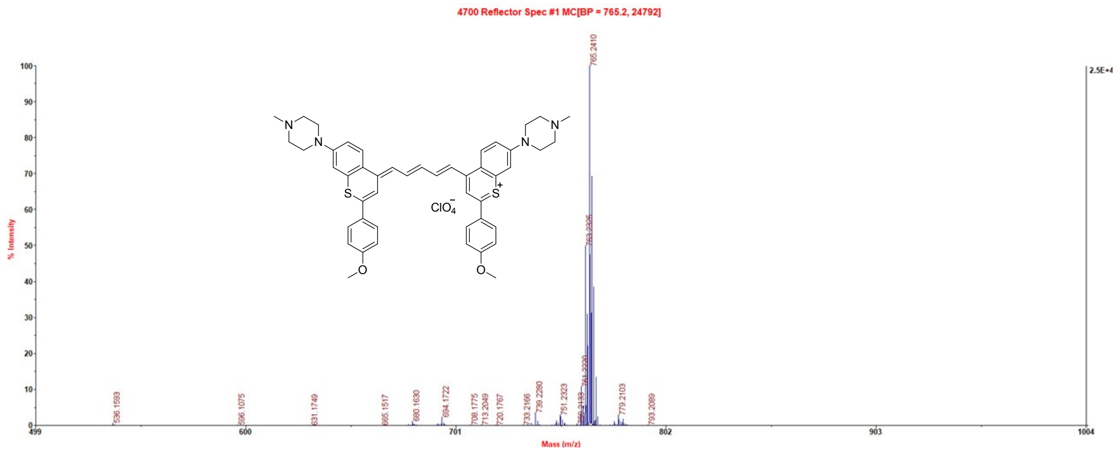

Figure S54. Maldi-Tof/Tof-MS spectrum of Lyso950.

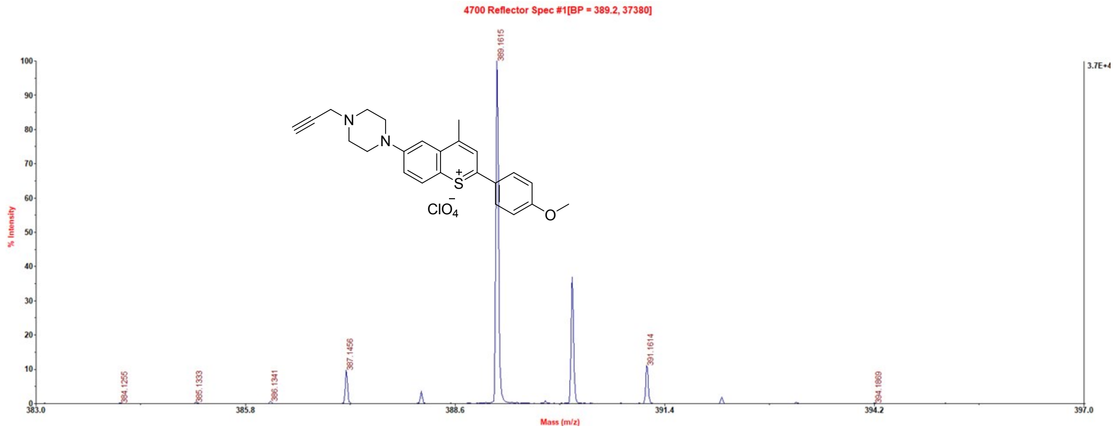

Figure S55. Maldi-Tof/Tof-MS spectrum of E4.

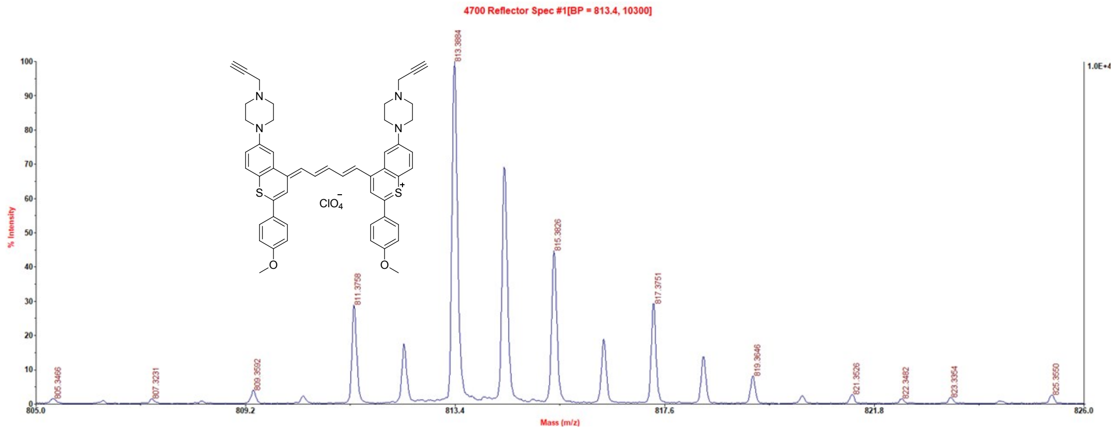

Figure S56. Maldi-Tof/Tof-MS spectrum of *prop*-Lyso1005.

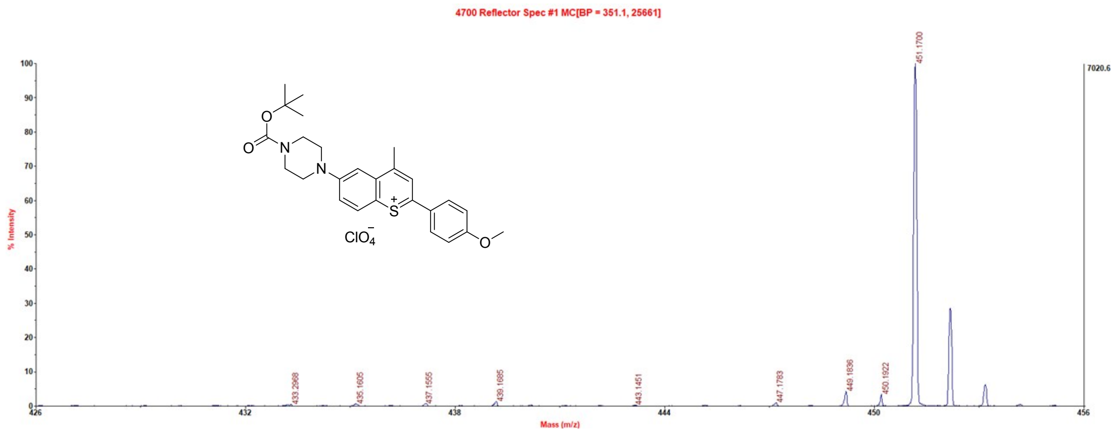

Figure S57. Maldi-Tof/Tof-MS spectrum of F1.

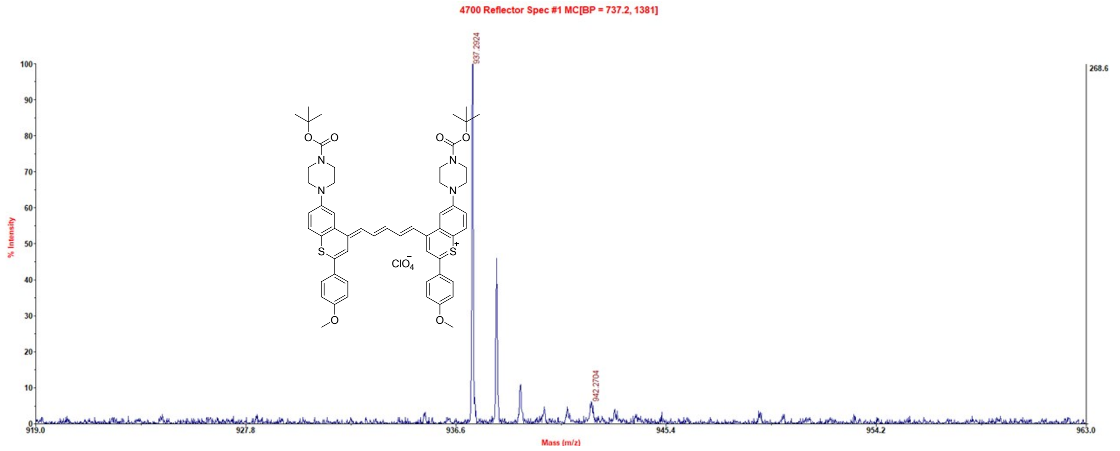

Figure S58. Maldi-Tof/Tof-MS spectrum of *boc*-Lyso1005.

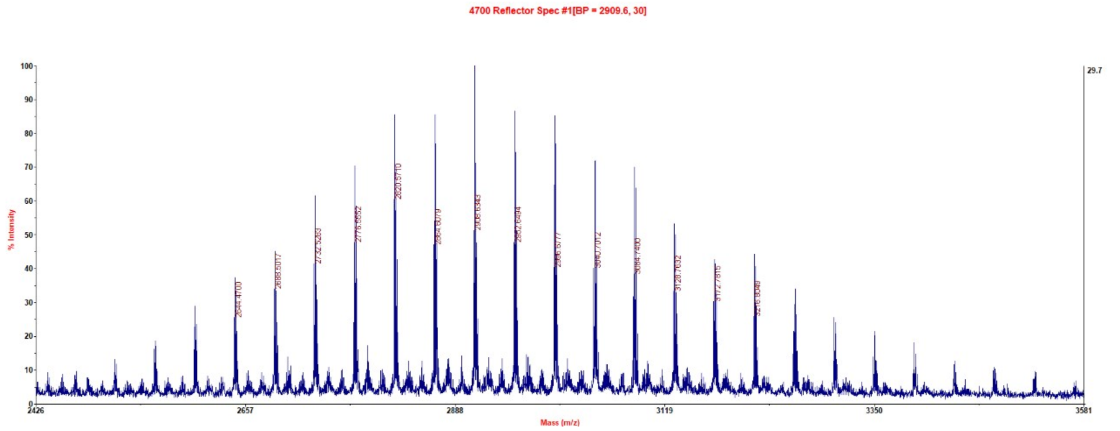

Figure S59. Maldi-Tof/Tof-MS spectrum of CEAF-OMe.

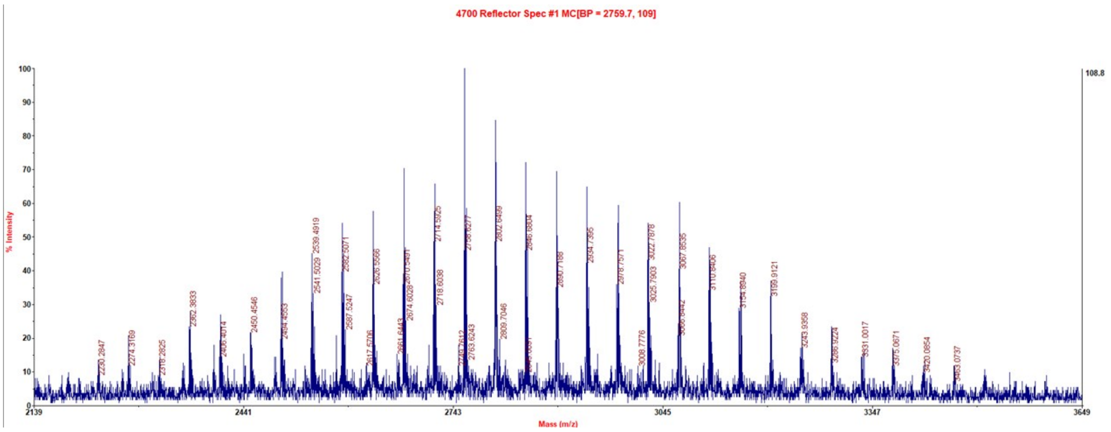

Figure S60. Maldi-Tof/Tof-MS spectrum of CEAF-NHS.

References

[1] B. Willy, W. Frank, T. J. Muller, *Org Biomol Chem* **2010**, *8*, 90-95.

[2] S. Wang, Y. Fan, D. Li, C. Sun, Z. Lei, L. Lu, T. Wang, F. Zhang, *Nat. Commun.* **2019**, *10*, 1058.

[3] O. Semonin, J. Johnson, J. Luther, A. Midgett, A. Nozik, M. Beard, *Journal of Physical Chemistry Letters - J PHYS CHEM LETT* **2010**, *1*, 2445-2450.

[4] M. J. Frisch, G. W. Trucks, H. B. Schlegel, G. E. Scuseria, M. A. Robb, J. R. Cheeseman, G. Scalmani, V. Barone, B. Mennucci, G. A. Petersson, H. Nakatsuji, M. Caricato, X. Li, H. P. Hratchian, A. F. Izmaylov, J. Bloino, G. Zheng, J. L. Sonnenberg, M. Hada, M. Ehara, K. Toyota, R. Fukuda, J. Hasegawa, M. Ishida, T. Nakajima, Y. Honda, O. Kitao, H. Nakai, T. Vreven, J. A. Montgomery, Jr., J. E. Peralta, F. Ogliaro, M. Bearpark, J. J. Heyd, E. Brothers, K. N. Kudin, V. N. Staroverov, T. Keith, R. Kobayashi, J. Normand, K. Raghavachari, A. Rendell, J. C. Burant, S. S. Iyengar, J. Tomasi, M. Cossi, N. Rega, J. M. Millam, M. Klene, J. E. Knox, J. B. Cross, V. Bakken, C. Adamo, J. Jaramillo, R. Gomperts, R. E. Stratmann, O. Yazyev, A. J. Austin, R. Cammi, C. Pomelli, J. W. Ochterski, R. L. Martin, K. Morokuma, V. G. Zakrzewski, G. A. Voth, P. Salvador, J. J. Dannenberg, S. Dapprich, A. D. Daniels, O. Farkas, J. B. Foresman, J. V. Ortiz, J. Cioslowski, and D. J. Fox, Gaussian, Inc., Wallingford CT, **2013**.

[5] R. M. Bolzan, R. Cueto, G. L. Squadrito, R. M. Uppu, W. A. Pryor, in *Methods Enzymol., Vol. 301*, Academic Press, **1999**, pp. 178-187.

[6] B. K. Davis, *Methods in molecular biology (Clifton, N.J.)* **2013**, *1031*, 27-35.
